# Mechanical constraints regulate embryonic stem cell mitosis in the developing brain

**DOI:** 10.1038/s44318-026-00848-3

**Published:** 2026-07-10

**Authors:** Véronique Marthiens, Lin Jawish, Margaux Malosse, Clara Basto, Denis Krndija, Anne-Sophie Macé, Elmar Laigna, Antonia Terrizzano, Corentin Peyret, Teng Pan, Olivier Zajac, Louisiane Perrin, Elsa Logarinho, Catherine Villard, Renata Basto

**Affiliations:** 1 Biology of Centrosomes and Genetic Instability team, Institut Curie, Centre National de la Recherche Scientifique (CNRS) Unité Mixte de Recherche 144, Université Paris Sciences et Lettres (PSL Research University), Paris, France; 2 Cell Migration and Invasion team, Institut Curie, CNRS-UMR144, PSL Research University, Paris, France; 3 Molecular, Cellular and Development Biology Unit (MCD) – Centre for Integrative Biology (CBI), University of Toulouse, CNRS, Toulouse, France; 4 Cell and Tissue Imaging Facility (PICT-IBiSA), Institut Curie, CNRS-UMR 144, PSL Research University, Paris, France; 5 Institut Curie, CNRS-UMR 168, Université Paris Sciences et Lettres, Paris, France; 6 Aging and Aneuploidy Laboratory, i3S - Instituto de Investigação e Inovação em Saúde, IBMC - Instituto de Biologia Molecular e Celular, Universidade do Porto, Porto, Portugal; 7 Université Paris Cité, CNRS-UMR 8236, Paris, France

**Keywords:** Cell Adhesion, Polarity & Cytoskeleton, Cell Cycle, Neuroscience

## Abstract

Research on mitosis has predominantly examined cell-autonomous mechanisms, yet its regulation and consequences within the broader physiological context remain largely unexplored. It remains, therefore, unknown whether and how spindle assembly and chromosome segregation are influenced by tissue properties. We have investigated this question in the mammalian embryonic brain, using high-resolution microscopy combined with pharmacological perturbations and minimal cell systems to break down the contribution of tissue environment to mitotic fidelity. We found that cell density imposes biomechanical constraints upon the apical radial glial (aRG) cell population during their highly proliferative period, which enhances spindle pole microtubule polymerization rates and potentiates chromosome mis-segregation. Mechanistically, we show that cortical tension relying on branched actin organization at the cell cortex conveys mechanical stress exerted by cell density. We identified the microtubule depolymerase MCAK/Kif2C as a critical effector downstream of cortical actin, linking actin dynamics to spindle pole activity. Altogether, our findings demonstrate that aRG are mechanosensitive during their expansion phase, and that excessive biomechanical stress, imposed by high cell density in developing tissues, can disrupt mitotic fidelity.

## Synopsis

Non-cell-autonomous effects of tissue properties on mitotic spindle assembly and chromosome segregation remain incompletely explored. This work shows that mammalian neural stem cells sense mechanical forces imposed by cell density in mitosis.

- Mouse embryonic neural stem cells respond to mechanical compression.
- High cell-density enhances microtubule polymerization in mitosis.
- Cortical actin branched via Arp2/3 conveys mechanical compression.
- Cortical actin is instructive for MCAK/Kif2C microtubule-depolymerase activity.
- Chromosome mis-segregation is increased when cell density is high.

## Abstract (web summary)

Elevated density increases branched cortical actin, maintaining low MCAK/Kif2C activity to promote spindle pole microtubule polymerization and chromosome mis-segregation in neural stem cells.

## Graphical abstract

**Figure:**
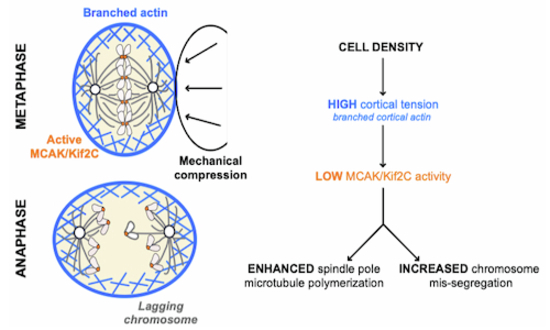

**Figure:**
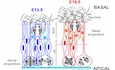

## Introduction

The mechanisms controlling chromosome behavior during mitosis have long been studied outside of native tissues in mammals. Cell division requires the orchestration of key events such as cell rounding, mitotic spindle assembly, chromosome condensation, and repartition in the two daughter cells (Lancaster and Baum, 2014; Taubenberger et al, 2020). Such a complex event relies on cell-autonomous and non-cell-autonomous factors that sense and coordinate the activity of many mitotic players to achieve a common goal, the generation of two genetically identical euploid daughter cells. Although aneuploidy, the gain or loss of whole chromosomes (Maiato and Silva, 2023; Santaguida, 2024) is known to depend on multiple factors and is often described as tissue context-dependent (Ben-David and Amon, 2020), the precise nature of the “context “remains poorly understood. Moreover, the contribution of tissue biomechanical properties to spindle assembly and chromosome segregation fidelity has remained unexplored.

The embryonic mammalian brain, and in particular the cerebral cortex, among other brain regions, is attractive to address this contribution. In the past 20 years, several studies have reported the occurrence of mutations in genes encoding mitotic proteins in human MicroCephaly Primary Hereditary (MCPH) patients, who exhibit a marked reduction in neocortex size but unperturbed size of other organs at birth (Thornton and Woods, 2009; Kaindl et al, 2010; Jayaraman et al, 2018; Naveed et al, 2018; Farcy et al, 2023). In animal models, the corresponding MCPH mutations lead to spindle assembly defects and result in chromosome segregation errors (Lizarraga et al, 2010; McIntyre et al, 2012; Marthiens et al, 2013; Martin et al, 2014; Marjanović et al, 2015; Shi et al, 2019). These defects result in reduction of the aRG cell population by multiple mechanisms, including aneuploidy-induced cell death and/or premature neuronal differentiation (Lizarraga et al, 2010; Marthiens et al, 2013; Rujano et al, 2013; Gogendeau et al, 2015; Marjanović et al, 2015; Shi et al, 2019; Viais et al, 2021), activation of the mitotic stopwatch machinery with consequent loss of proliferative potential (Phan et al, 2021) and transient lagging chromosomes, which can experience DNA damage (Janssen et al, 2011; Crasta et al, 2012).

**Figure 1:**
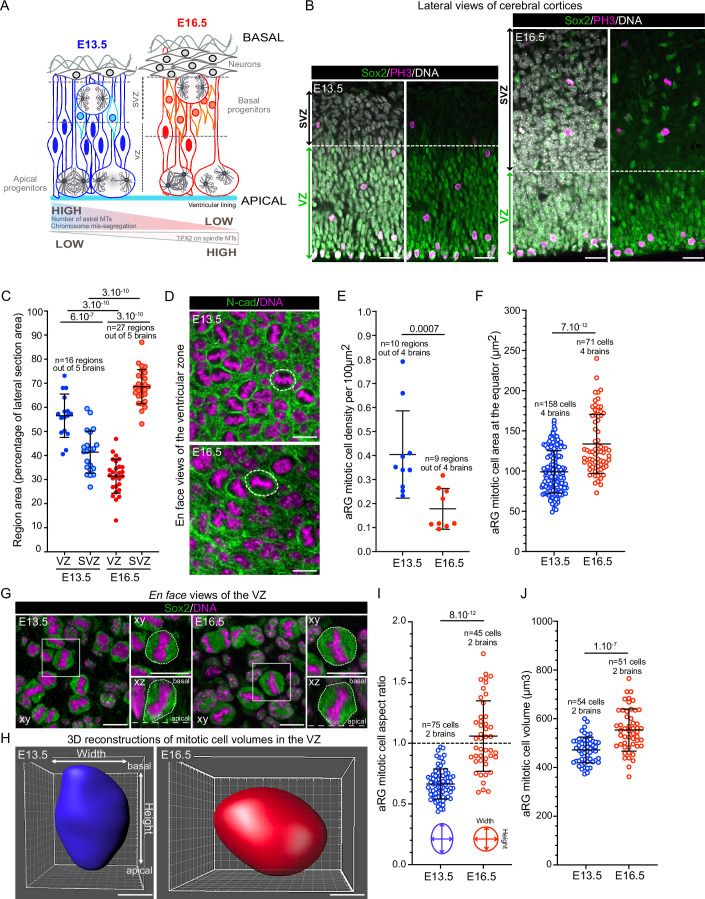
aRG mitotic cell morphometrics during neurogenesis. (**A**) Schematic diagram comparing the organization of the neuroepithelium along the apicobasal axis during neurogenesis at embryonic day 13.5 (E13.5) and 16.5 (E16.5). Apical Radial Glial cells (aRGs)—also called apical progenitors (APs)—populate the ventricular zone (VZ), lined by the ventricle, and basal progenitors the sub-ventricular zone (SVZ). Neurons (gray nuclei) reside in the upper layers. APs and BPs in blue at E13.5 and red at E16.5. (**B**) Lateral views of cerebral cortices at E13.5 and E16.5 showing the stem cell marker Sox2 in green, Phospho-Histone 3 (PH3) in magenta, with or without DNA in gray. (**C**) Scatter dot plot of the indicated region area as a fraction of the lateral area. (**D**) Pictures of whole-mount cerebral cortex explants showing nuclei in magenta and the plasma membrane labeled with N-cadherin (N-cad) antibodies (green). (**E**, **F**) Scatter dot plots of mitotic aRG cell density (**E**) and equatorial area (**F**). (**G**) Pictures of *en face* and lateral views of whole-mount cerebral cortex explants. Sox2 is in green and nuclei in magenta. (**H**) 3D-reconstruction of aRG cell volumes from the indicated developmental stages. (**I**, **J**) Scatter dot plots graphs. The black dotted line points to an aspect ratio of 1, which corresponds to a round shape mitosis. All data in (**C**, **E**, **F**, **I**, **J**) are presented as mean ± SD with a center line at the mean value and error bars indicating SD. Data were submitted to normality and lognormality tests for the choice of an appropriate statistical test. Statistical significance was assessed with ordinary one-way ANOVA (C), Mann–Whitney test (**E**, **F**), or Welch’s test (**I**, **J**). Scale bars are (**B**) 20 μm; (**D**, **G**) 10 μm; and (**H**) 4 μm. Source data are available online for this figure.

During brain development, apical Radial Glial (aRG) cells give rise to all the neuronal and glial cell populations that populate the brain at birth (Paridaen and Huttner, 2014; Villalba et al, 2021; Casas Gimeno and Paridaen, 2022). They proliferate at high rates in a restricted time window, between embryonic day 10 (E10) and birth (around E18-E20) in the mouse, to timely generate the typical six neuronal layers of the cerebral cortex (Calegari et al, 2005; Arai et al, 2011). They keep bipolar attachments during the cell cycle to maintain apicobasal polarity in the neuroepithelium (Fig. 1A) and undergo interkinetic nuclear migration coordinated with the cell cycle (Taverna and Huttner, 2010; Okamoto et al, 2013; Shimamura and Miyata, 2024), where G1 and S-phase nuclei are closer to the basal side, while G2 nuclei move towards the apical side for mitosis. This results in the formation of a pseudo-stratified neuroepithelium that accommodates the dividing aRG population in the so-called ventricular zone (VZ) (Fig. 1A). From the beginning and up until mid-neurogenesis (E14.5), progenitor cell populations expand through proliferative divisions, while the size of the VZ does not grow proportionally. As a result, aRG confinement in the VZ increases in a stepwise manner until they delaminate while giving rise to basal progenitors (Paridaen and Huttner, 2014; Villalba et al, 2021). Thus, aRGs until mid-neurogenic stages undergo cell division with biomechanical constraints driven by high cell density, nuclear movements, and progeny migration toward basal layers (Okamoto et al, 2013; Shimamura and Miyata, 2024). Altogether, the temporal and spatial constraints typical of neocortex development render this tissue ideal to study the impact of biomechanical constraints on aRG mitosis.

It has been shown that confinement imposed on cells in culture is sufficient to influence mitotic spindle shape, leading to chromosome mis-segregation (Matthews et al, 2020; Lancaster et al, 2013). Whether this is also the case in the developing mammalian brain remains to be investigated.

We have previously shown that aRGs from the mouse cerebral cortex are more prone to generate chromosome segregation errors during their expansion period (Marthiens et al, 2013; Vargas-Hurtado et al, 2019; Marthiens and Basto, 2020). Here, we have investigated the changes in biomechanical properties of aRG cells in mitosis within the VZ during neurogenesis. We found that in the first half of neurogenesis, when they undergo mitosis under high levels of constraint, aRG cells are more responsive to the mechanical confinement imposed by their neighbors. Mechanistically, we show that aRG cells submitted to compressive stress display high cortical rigidity conveyed by branched actin filaments at the plasma membrane. We further identify cortical actin as a modulator of MCAK/Kif2C microtubule (MT)-depolymerase activity, influencing MT polymerization activity at the spindle poles and chromosome segregation. Our data establish a functional connection between cortical rigidity and MCAK activity in regulating mitotic fidelity during mammalian brain development.

## Results

### Cell density correlates with morphometric changes in the developing mouse neuroepithelium

**Figure EV1:**
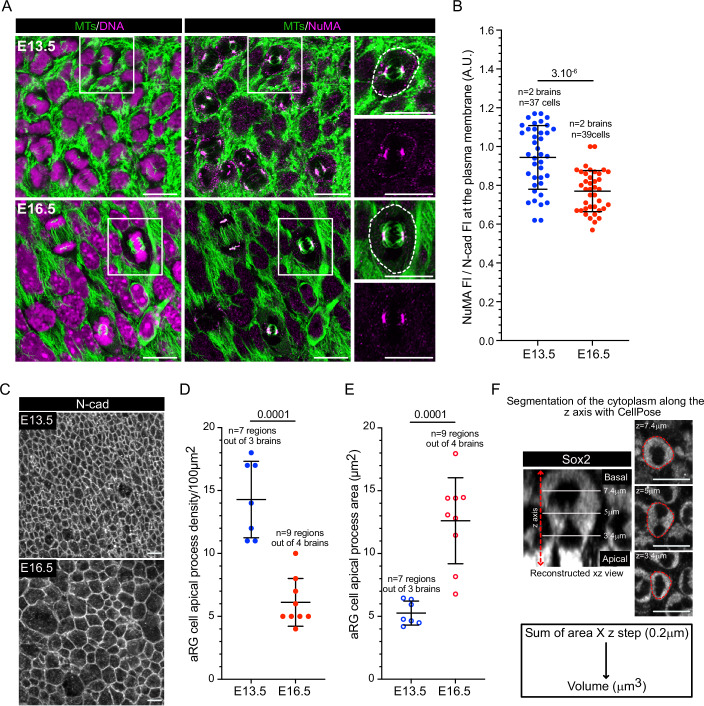
Analysis of aRG cell density as neurogenesis progresses. (**A**) Pictures of *en face* views of whole-mount cerebral cortical explants from the indicated neurogenic stages showing MTs in green and DNA in magenta on the left and MTs in green and NuMA in magenta on the right. Insets show higher magnifications of mitotic cells. Dotted white lines highlight cell boundaries. (**B**) Scatter dot plot showing the ratio between NuMA/N-Cad FI level at the plasma membrane. (**C**) Pictures of *en face* views of cortical explants from the indicated neurogenic stages showing N-Cad signal. (**D**, **E**) Scatter dot plots showing aRG cell apical process density (**D**) and area (**E**). (**F**) Images showing the methods used to quantify mitotic aRG cell volumes. Lateral (xz, left panel) and *en face* (xy, right panels) views of a Sox2-positive aRG mitosis. Dotted red lines indicate examples of measured areas at different z positions. All data in (**B**, **D**, **E**) are presented as mean ± SD with a center line at the mean value and error bars indicating SD. Data were submitted to normality and lognormality tests for the choice of an appropriate statistical test. Statistical significance was assessed with a Mann–Whitney test (**B**) and Welch’s tests (**D**, **E**). Scale bars: (**A**) 10 μm; (**C**) 5 μm; (**F**) 10 μm. Source data are available online for this figure.

aRG cells (aRGs) undergo mitosis in the ventricular zone (VZ) close to the ventricle and are therefore also referred to as apical progenitors (APs). We have previously correlated changes in mitotic spindle morphology with mitotic fidelity during neurogenesis of the mouse cerebral cortex (Vargas-Hurtado et al, 2019). Differences were obvious when comparing aRG mitosis at embryonic day 13.5 (E13.5) and 16.5 (E16.5). In particular, we observed an enrichment of the population of astral microtubules at E13.5 (Fig. EV1A,B), at the expense of the population of spindle microtubules (Vargas-Hurtado et al, 2019). At E13.5, spindles are strictly positioned along the apicobasal axis (Fig. 1A) thanks to interactions between astral MTs and the plasma membrane (Mora-Bermúdez et al, 2014), illustrated by a higher recruitment of the Nuclear Mitotic Apparatus (NuMA) protein at the plasma membrane (Fig. EV1A,B) (Machicoane et al, 2014; Kiyomitsu and Boerner, 2021).

To probe the biomechanical constraints faced by mitotic aRG cells and identify changes in morphometrics parameters during neurogenesis, we analyzed the VZ during aRG expansion at two distinct developmental stages- at E13.5 and E16.5. A twofold decrease in the area occupied by aRG cells (identified by the stem cell marker Sox2) at the VZ was evident in lateral sections of the neuroepithelium (Fig. 1B,C). Accordingly, a threefold decrease in the density of apical processes at the ventricular lining (Fig. EV1C,D, as a readout of aRG density in interphase) and mitotic aRGs (Fig. 1D,E) was observed. The aRG density decline was accompanied by an increase in the area occupied by apical processes (Fig. EV1E) and mitotic cells at the equator (Fig. 1F), which are good indicators of spatial constraint release. Moreover, lateral constraints imposed by cell density appeared to impact both mitotic cell shape, as evidenced by a change in aRG aspect ratios (ratios of cell width over height) from an elongated shape at E13.5 (mean aspect ratio at 0.65) to a circular shape at E16.5 (mean aspect ratio at 1) (Fig. 1G–I) and a concomitant increase in cell volume (Figs. 1J and EV1F).

Our findings show that E13.5 aRG cells are more constrained due to increased cell density in the VZ, as reflected by changes in their shape and volume.

### Mechanical confinement imposed by high cell density promotes mitotic errors in aRG

**Figure 2:**
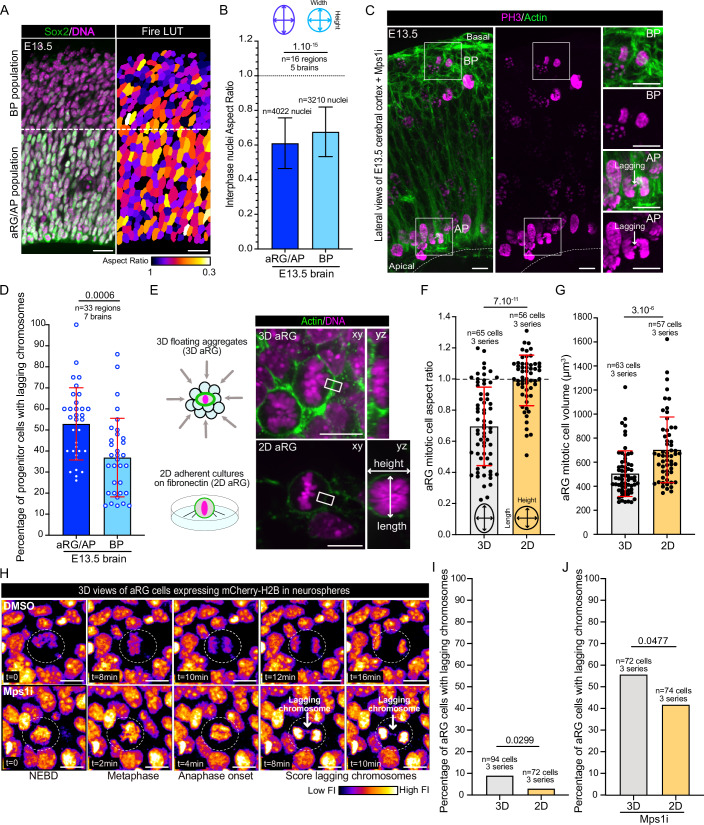
The frequency of chromosome segregation errors is influenced by cell density. (**A**) On the left, a picture of a lateral view of a cerebral cortex labeled as indicated; On the right, Fire LUT aspect ratio representation of the corresponding regions. (**B**) Bar graph depicting aspect ratio values of interphase nuclei along the apical–basal axis. (**C**) Cerebral cortex lateral view labeled with PH3 (magenta) and actin (green), insets are shown on the right to illustrate anaphases with and without lagging chromosomes. (**D**) Scatter dot bar plot showing the percentage of anaphases with lagging chromosomes. (**E**) On the left, a schematic diagram showing 3D (top) and 2D (bottom) experimental set-ups for aRG primary cell cultures. On the right, pictures showing *en face* views of aRG cell cultures with actin in green and DNA in magenta. (**F**, **G**) Scatter dot bar plots showing aRG mitotic cell aspect ratios and volumes in the indicated settings. (**H**) Stills of time-lapse movies of aRG cells from prophase to anaphase expressing mCherry-H2B to visualize DNA (in Fire LUT based on mCherry fluorescence intensity- FI) treated with Mps1i. The last two panels on the right display anaphases with (bottom) or without (top) lagging chromosomes (white arrows). (**I**, **J**) Bar graphs comparing the percentage of aRG cells with lagging chromosomes scored by live imaging. All data in (**B**, **D**, **F**, **G**) are presented as mean ± SD with a center line at the mean value and error bars indicating SD. Data were submitted to normality and lognormality tests for the choice of an appropriate statistical test. Statistical significance was assessed with Mann–Whitney tests (**B**, **F**, **G**), Welch’s test (**D**), and chi-square on normalized contingency data (**I**, **J**). Scale bars: (**A**) 15 μm; (**C**) 12 μm; (**E**, **H**) 10 μm. Source data are available online for this figure.

We next analyzed the population of progenitor cells at E13.5, which consists of two main cell types—apical progenitors (AP) and basal (BP) progenitors, which are generated through delamination of APs and positioned within the sub-ventricular zone (SVZ) (Fig. 1A) (Taverna and Huttner, 2010; Arai and Taverna, 2017). Interestingly, differences in nuclear shape, estimated by aspect ratio measurements, were noticed (Fig. 2A,B), indicating that APs are more confined in the VZ as compared to BPs in the SVZ. In order to challenge mitotic fidelity in these two populations, we incubated E13.5 cerebral cortex slices with the spindle assembly checkpoint (SAC) Mps1 inhibitor (Mps1i) (Santaguida et al, 2010; Tardif et al, 2011) for a short period of time (3 h). The number of mitotic cells identified after phospho-histone 3 (PH3) labeling showing lagging chromosomes in anaphase was higher in the AP than in the BP population (Fig. 2C,D). Altogether, these results highlight a correlation between spatial constraints and mitotic errors in vivo in the developing brain.

**Figure EV2:**
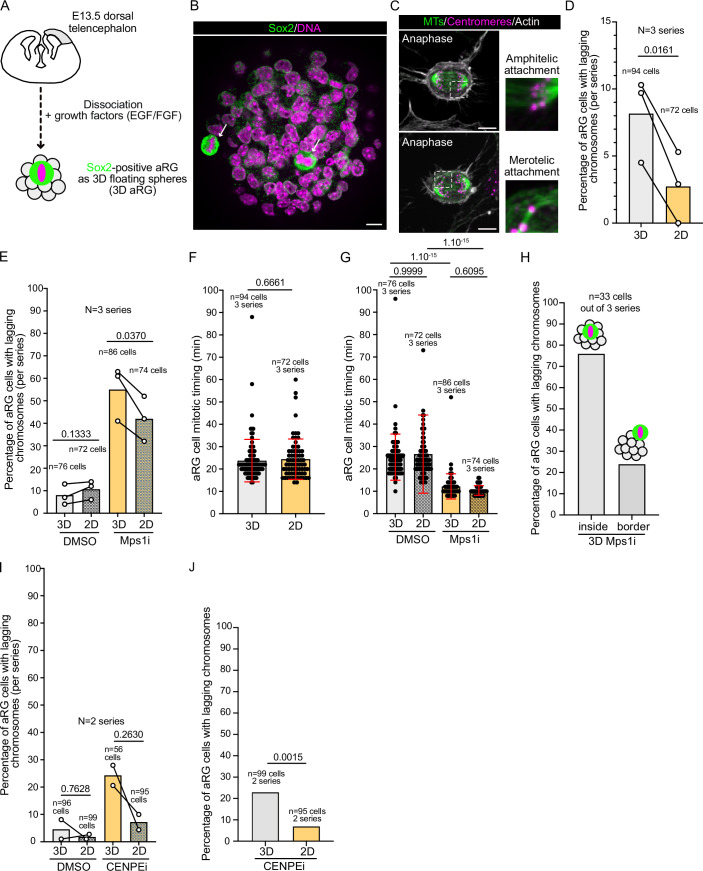
Mitotic timing does not account for differences between 3D and 2D aRG cell primary cultures. (**A**) Schematic representation of the protocol used to establish primary Sox2-positive aRG cultures from E13.5 dorsal telencephalon. (**B**) Picture of 3D aRG cells forming a floating sphere showing Sox2 (green) and DNA (magenta). (**C**) Pictures of aRG anaphase cells showing MTs (green), centromeres (magenta), and actin (gray) to illustrate amphitelic and merotelic attachments in the insets. (**D**, **E**) Bar graphs showing the mean frequency of lagging chromosomes and paired values for each series. (**F**, **G**) Scatter dot bar plots showing the mitotic timing. (**H**) Bar graph showing the frequency of cells with lagging chromosomes according to their position at the center or periphery of the neurosphere. (**I**, **J**) Bar graph showing the mean frequency of lagging chromosomes and paired values for each series (**I**) or the percentage of cells from cumulative analysis of the two series (**J**). All data in (**F**, **G**) are presented as mean ± SD with a center line at the mean value and error bars indicating SD. Data were submitted to normality and lognormality tests for the choice of appropriate statistics. Statistical significance was assessed with paired *t* test (**D**), ratio paired *t* tests (**E**, **I**), Mann–Whitney test (**F**), Kruskal–Wallis test (**G**), chi-square test on normalized contingency data in (**J**). Scale bars: (**B**) 10 μm and (**C**) 5 μm. Source data are available online for this figure.

To evaluate whether there is a causal relationship between aRG density and chromosome segregation errors, we established an ex vivo approach to experimentally manipulate the cell density of aRG cells in mitosis. We developed a protocol where aRG cells were dissociated from E13.5 mouse dorsal telencephalon to establish primary cell cultures (Fig. EV2A,B). These cultures were expanded either as 3D floating spheres—referred here as 3D aRG or plated on fibronectin substrates—called 2D aRG (Fig. 2E). Upon mitotic entry, 2D aRG like other cells in culture, de-adhere before rounding up (Taubenberger et al, 2020) and undergo mitosis without lateral cell–cell contacts (Fig. 2E). The morphometrics of aRG mitosis differed in the two experimental set-ups, with 2D aRG mitotic cells exhibiting a more circular shape (as evidenced by the measure of aspect ratios, i.e., cell height over cell length) (Fig. 2F) and increased cell volume (Fig. 2G), as compared to 3D aRG mitotic cells. The presence of neighboring cells around mitosis in culture is therefore sufficient to influence cell shape and volume, validating this simplified primary culture setting to mimic the conditions found in the developing brain.

We then compared mitotic fidelity in aRG cells with and without cell neighbors -3D and 2D conditions, respectively, by establishing cultures derived from a mCherry-Histone2B (mCherry-H2B) expressing mouse line. Using live imaging approaches, we scored the frequency of lagging chromosomes in anaphase (Fig. 2H) resulting from merotelic attachments (Fig. EV2C). The frequency of chromosome mis-segregation in unchallenged conditions was higher in 3D than in 2D settings (Fig. EV2D). The total number of cells displaying lagging chromosomes in the three sets of experiments performed decreased by fivefold when aRGs undergo mitosis without neighbors (Fig. 2I). In addition, Mps1i used at low doses (25 nM) in an acute manner—a maximum of 2.5 to 3 h time-lapse recording in the presence of the drug- also showed the same trend (Figs. 2J and EV2E). Importantly, these differences cannot be explained by differences in mitotic timing as a readout of mitotic progression (Fig. EV2F,G). Nonetheless, when we plotted the frequency of anaphases with lagging chromosomes according to their position within the neurosphere, we noticed that aRGs positioned at the border displayed less chromosome mis-segregation errors as compared to the cells inside the neurosphere (Fig. EV2H). This strengthened a causal relationship between the level of confinement and mitotic fidelity.

To determine the frequency of chromosome mis-segregation using an alternative pharmacological approach, we incubated mitotic aRGs with the Centromere Protein E (CENP-E) kinesin inhibitor (CENPEi). CENP-E is a component of the kinetochore *fibrous corona* that promotes chromosome congression and kinetochore-MT stability (Weaver et al, 2003; Gudimchuk et al, 2013; Vitre et al, 2014). Similar to Mps1i, CENPEi resulted in a higher frequency of chromosome segregation errors in aRG cells cultured in 3D than in 2D (Fig. EV2I,J).

Collectively, our data indicate that the presence of neighboring cells promotes chromosome mis-segregation in both unchallenged and challenged conditions.

### Biomechanical constraints are sufficient to increase astral microtubule density

**Figure 3:**
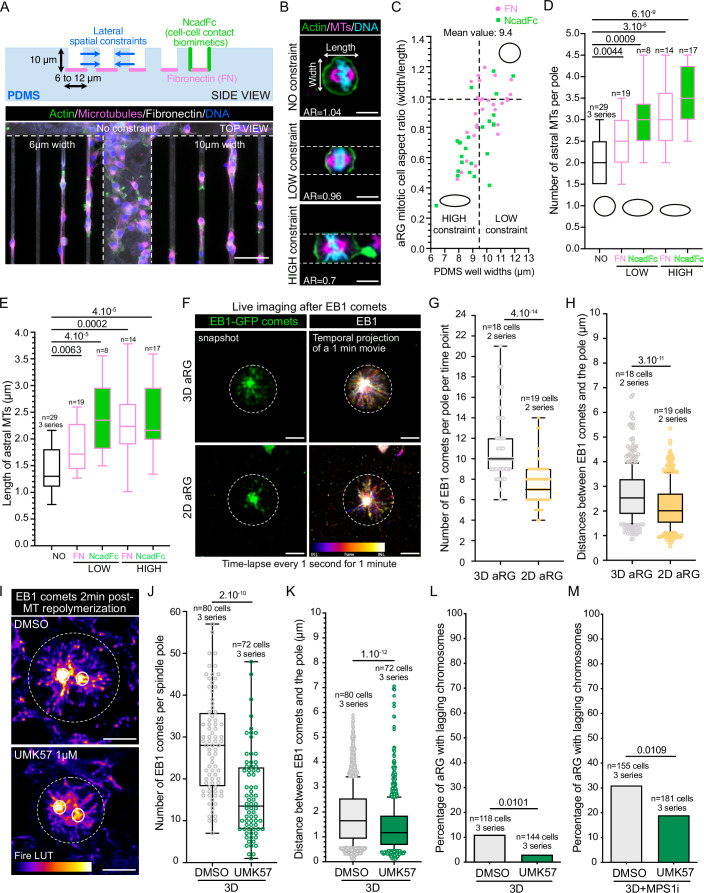
Biomechanical constraints modulate the rates of spindle pole MT polymerization. (**A**) Top, schematic diagram of the side view of the PDMS device used to spatially constrain mitotic aRG cells. Cells were plated on fibronectin (red) in trenches of different widths ranging from 6 to 12 μm. Certain trenches were coated with the N-cadherin biomimetic Ncad-Fc (green). Pictures of E13.5 aRG cells constrained in fibronectin-coated lanes (gray) and visualized with actin (green), MTs (red), and nuclei (cyan). Cells were either plated outside of the trenches (no constraint), in trenches of 6 μm (left side) or 10 μm (right side) widths. (**B**) Higher magnifications of aRG cells in metaphase outside of constraint (NO constraint- aspect ratio (AR) 1.04), under low (AR: 0.96) or high (AR: 0.7) constraints. (**C**) Scatter dot plot depicting aspect ratios of individual aRG cells in metaphase according to PDMS width. (**D**, **E**) Box and whiskers plots representing the number (**D**) and length (**E**) of astral MTs. Data are provided for cells outside of constraints (NO, empty black boxes), for cells plated on FN in the PDMS wells (empty red boxes), and for cells in wells coated with Ncad-Fc (red boxes filled in green). (**F**) Representative pictures of aRG cells in prometaphase as indicated, showing EB1-positive MT (+) tips polymerized from one pole represented at one time point (left panels, EB1 in green) or as a temporal maximum projection during a 1 min long MT repolymerization movie with acquisitions taken every second (right panels). False colors represent the time when the EB1 comets were first detected during the 1 min recording. (**G**, **H**) Box and whisker plots showing the number of EB1 comets polymerized per pole at a given time point (**G**) and the maximal distance traveled by individual EB1 comets from the spindle pole (**H**). (**I**) Representative images of aRG cells in prometaphase at *t *= 2 min after the start of MT repolymerization, showing the accumulation of EB1-positive MT (+) tips (false colors, with the fire LUT representing the levels of EB1 fluorescence intensity) around the two spindle poles. Note the asymmetry in MT polymerization rate between the two spindle poles. (**J**, **K**) Box and whiskers plots showing the cumulative number of EB1 comets polymerized per pole (**J**) and the distance traveled (**K**) under the noted conditions. (**L**, **M**) Bar graphs showing the percentage of cells displaying lagging chromosomes. Median values are indicated by the middle line, and whiskers range from minimum to maximum values (**D**, **E**, **G**–**J**) or 10 to 90 percentiles (**H**–**K**). Data were submitted to normality and lognormality tests for the choice of an appropriate statistical test. Statistical significance was assessed with Mann–Whitney tests (**D**, **G**, **H**, **J**, **K**), unpaired *t* test (**E**), and Fisher’s exact test (**L**, **M**). Scale bars: (**A**) 60 μm; (**B**, **F**, **I**) 5 μm. Source data are available online for this figure.

We have previously reported that aRG spindles are particularly enriched in astral MTs when they are prone to mitotic errors in the E13.5 cerebral cortex (Vargas-Hurtado et al, 2019). We therefore hypothesized that the biomechanical constraints faced by mitotic cells in the presence of neighbors (3D aRG cultures) may favor the nucleation or stabilization of astral MTs. To test this possibility, and in order to mimic the lateral constraints imposed to aRG mitotic cells in the brain, we seeded primary cultures of E13.5 aRG dissociated from the dorsal telencephalon (Fig. EV2A,B) on top of a polydimethylsiloxane (PDMS)-based device containing trenches of 10 µm in height and between 8 and 12 µm in width (Fig. 3A). This experimental set-up imposed passive lateral spatial constraints to isolated aRG cells as they undergo mitotic rounding. To facilitate cell seeding and survival, the trenches were coated with fibronectin at the bottom by using the technique of in-mold patterning (Yamada et al, 2016). Furthermore, the wells were coated with N-cadherin-Fc chimera (NcadFc) to mimic cell–cell adhesions. The level of constraints imposed were evaluated by the aRG cell aspect ratios as a measure of cell shape changes (Fig. 3B,C). This setup allowed us to use cells seeded outside of the wells as an internal control of cells without any constraint (Fig. 3A). A distinction between low (trench width above 9.4 µm) and high (trench width below 9.4 µm) levels of constraint was considered (Fig. 3B,C). Increasing constraint was sufficient to increase the number and length of astral MTs in a stepwise manner, which was further exacerbated by mimicking cell–cell adhesion (Fig. 3D,E). It is interesting to note that the length of astral MTs reached a plateau in conditions of high constraints, likely because of the cell size limit.

We concluded that the presence of lateral constraints is sufficient to increase the frequency and length of astral microtubules in mitotic spindles of aRG cells in culture.

### Spatial constraints, but not cell–cell adhesion per se, enhance spindle pole MT polymerization

**Figure EV3:**
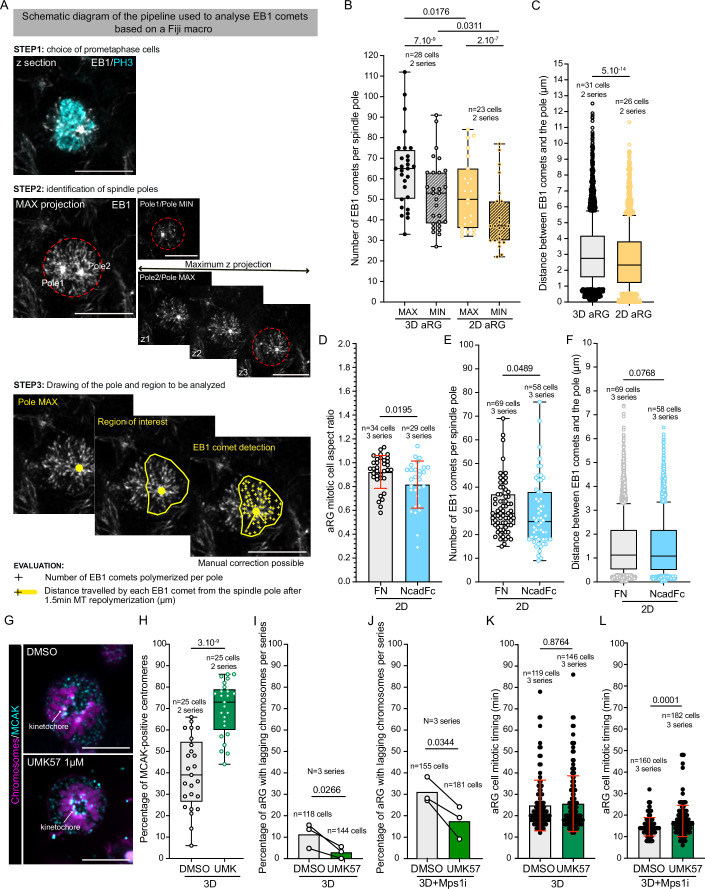
Analysis of MT depolymerization–repolymerization assays in 2D and 3D aRG cultures. (**A**) Schematic diagram of the analysis of EB1 comets after MT depolymerization–repolymerization assays. The first step consists of choosing a prometaphase cell thanks to PH3 labeling. In the second step, the pole to be quantified is chosen, taking into consideration the EB1 signal. A maximum projection containing three individual z-stacks is used. The region to be considered is manually segmented. Through the dedicated macro, the parameters extracted are the number of EB1 comets and the distance traveled from the pole. (**B**, **C**) Box and whiskers plots showing the cumulative number of EB1 comets (**B**) and distance traveled (**C**) per pole during 1.5 min MT repolymerization. (**D**) Scatter dot bar plot showing the aspect ratios of 2D aRG cells plated on different substrates. (**E**, **F**) Box and whiskers graphs showing the mean number of EB1 comets (**E**) and the distance traveled from the pole (**F**). (**G**) Pictures of aRG cells treated as indicated, labeled with MCAK antibodies (cyan). Chromosomes are shown in magenta. (**H**) Box and whiskers plot showing the percentage of kinetochores positive for MCAK per cell. (**I**, **J**) Bar graphs showing the mean frequency of lagging chromosomes and paired values for each series. (**K**, **L**) Scatter dot bar plot showing the mitotic timing. Data are mean ± SD in (**D**, **K**, **L**). Median values are indicated by the middle line, and whiskers range from minimum to maximum values (**B**, **E**, **H**) or 10–90 percentiles (**C**, **F**). Data were submitted to normality and lognormality tests for the choice of appropriate statistics. Statistical significance was assessed with the Wilcoxon matched-pairs signed rank test (**B**), unpaired *t* test (**H**), Mann–Whitney tests (**C**, **D**, **E**, **F**, **K**, **L**), ratio paired *t* test (**I**), and paired *t* test (**J**). Scale bars: (**A**) 12 μm and (**G**) 10 μm. Source data are available online for this figure.

The increase in astral MT number and length under constraint suggested increased MT polymerization capacity from the spindle poles. We investigated MT polymerization activity by tracking plus-end EB1-positive comets as a readout of actively polymerizing MT plus tips in mitosis (Muroyama and Lechler, 2017; McHugh and Welburn, 2023). Comparison between 3D and 2D cultures by live imaging approaches revealed higher MT polymerization rates in 3D aRG cells, illustrated by 1.5-fold higher number of EB1 comets polymerized per time point (Fig. 3F,G). Moreover, the distance traveled by EB1 comets from the spindle pole, which reflects MT polymerization persistence (Muroyama and Lechler, 2017; Roostalu et al, 2020), was also higher (Fig. 3H). Our observations were similar after MT depolymerization–repolymerization assays performed on a short time scale (1.5 min repolymerization), evaluating the same parameters for individual EB1 comets (Fig. EV3A–C). Importantly, MT polymerization activity was different between the two spindle poles, regardless of whether aRG cells underwent mitosis in 3D or 2D (Fig. EV3B). This suggests that confinement does not perturb the spindle pole asymmetry, which was also observed in vivo (Vargas-Hurtado et al, 2019). We therefore decided to plot the data of the pole displaying the highest activity.

We next determined whether the density of cell–cell adhesion was triggering the changes in spindle pole MT polymerization capacity. 2D aRG cultures were plated on the cell–cell contact biomimetic Ncad-Fc chimera instead of fibronectin (Fig. EV3D–F). Interestingly, while mitotic rounding was affected by the presence of Ncad-Fc as a substrate, as illustrated by the flattened morphology of the cells at the interface with the substrate (Aspect Ratio below 1, as compared to the FN control substrate) (Fig. EV3D), the number and length of actively polymerizing MTs were not affected (Fig. EV3E,F). These observations support the conclusion that N-cadherin-dependent cell–cell contacts are not promoting spindle pole MT polymerization activity but are more likely stabilizing the population of astral MTs at the plasma membrane (Fig. 3D,E).

Together, we show that during mitosis of aRG cells, biomechanical constraints imposed by neighboring cells can modulate the rate of MT polymerization at the spindle poles, with a poor contribution of cell–cell adhesion to this behavior.

### Biomechanical constraints enhance spindle pole polymerization activity and chromosome mis-segregation

MT dynamic instability is achieved by a balance between MT polymerization and depolymerization events (Mitchison and Kirschner, 1984). Depolymerases such as MCAK/Kif2C play key roles in mitotic spindle assembly and chromosome segregation in many cell types (Stout et al, 2011; Tanenbaum et al, 2011; Walczak et al, 2016; van Heesbeen et al, 2017; McHugh and Welburn, 2023). We functionally tested whether counteracting the high polymerization activity typical of 3D aRG mitosis could lower the frequency of chromosome mis-segregation. To this end, we used the small-molecule agonist of MCAK motor activity— UMK57—which decreases MT polymerization and the frequency of lagging chromosomes in aged human fibroblasts (Barroso-Vilares et al, 2020). To validate the effect of UMK57 in our experimental setup, we incubated 3D aRG cells for 1 h with UMK57 and evaluated the sub-cellular localization of MCAK in prometaphase cells. We observed that the treatment was sufficient to increase by almost twofold the number of kinetochores positive for MCAK (Fig. EV3G,H). In 3D aRG cells, this treatment had the expected effect of decreasing the number and length of actively polymerizing MTs (Fig. 3I–K). Importantly, a decrease in the frequency of lagging chromosomes (Figs. 3L and EV3I), without differences in mitotic timing (Fig. EV3K) was also observed. Moreover, UMK57 treatment was sufficient to reduce the frequency of lagging chromosomes in 3D aRG cells challenged with Mps1i (Figs. 3M and EV3J), while the combination of Mps1i and UMK57 starts delaying mitotic progression (Fig. EV3L).

We concluded that in mitotic aRG cells in 3D and thus under constraint, counteracting spindle pole MT polymerization rates is sufficient to decrease the frequency of chromosome mis-segregation.

### Mechanical compression enhances MT polymerization and promotes chromosome mis-segregation

**Figure 4:**
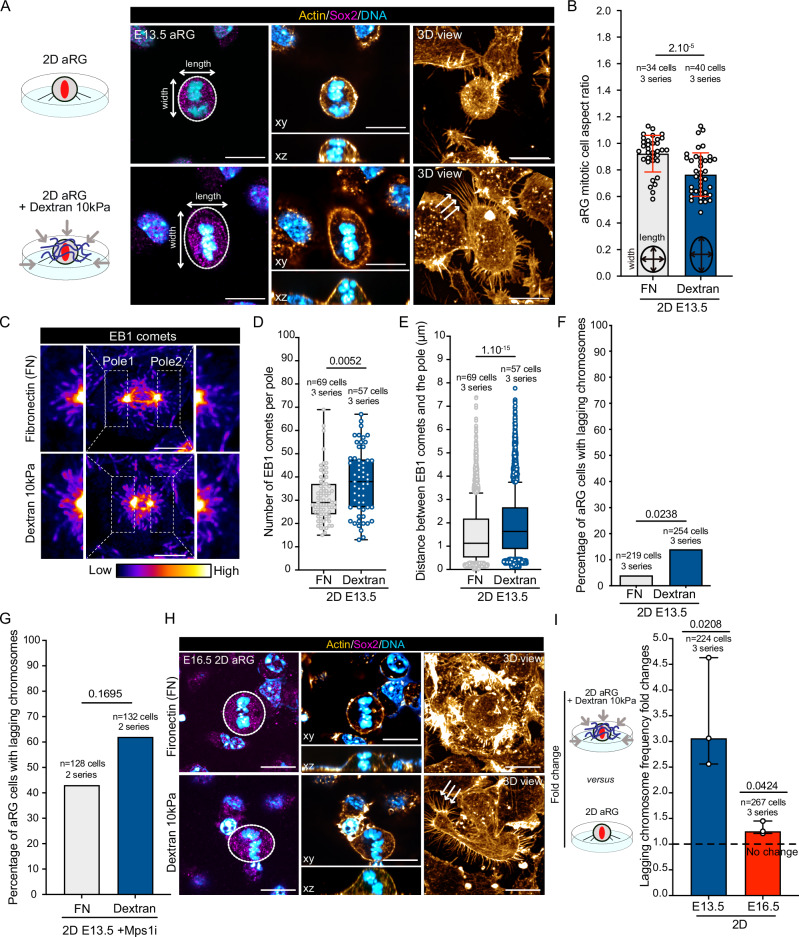
Compression of 2D aRG cells is sufficient to increase MT polymerization rates and chromosome mis-segregation. (**A**) On the left, a schematic diagram of 2D aRG cells with (bottom panels) or without (top panels) dextran to impose compression of 10 kPa. On the right, pictures of equatorial views (left panels) or substrate view (right panels) of aRG cells in metaphase showing actin (gold), Sox2 (magenta), and DNA (cyan). Small white arrows point to retraction fibers. The white dotted line highlights the cell border. (**B**) Scatter dot bar plot showing 2D aRG aspect ratios evaluated at the substrate level. (**C**) Pictures of EB1 comets from aRG cells in metaphase. High magnifications of the two spindle poles are provided as left and right insets. The fire LUT was used to represent the levels of EB1 fluorescence intensity. (**D**, **E**) Box and whiskers plots showing the number of EB1 comets per pole (**D**) and corresponding EB1 comet distances traveled from the pole (**E**) in the absence (Fibronectin substrate only, FN) or presence of dextran. Median values are indicated by the middle line, and whiskers range from minimum to maximum value (**D**) or 10–90 percentiles (**E**). (**F**, **G**) Bar graphs showing the frequency of lagging chromosomes. (**H**) Pictures of equatorial views (left panels) or substrate views (right panels) of aRGs in metaphase labeled. Small white arrows point to retraction fibers. The white dotted line highlights the cell border. (**I**) Bar graphs comparing fold change increase in the frequency of lagging chromosomes in 2D aRG cultures. Data are mean ± SD. Data were submitted to normality and lognormality tests for the choice of an appropriate statistical test. Statistical significance was assessed by Mann–Whitney tests (**B**, **D**, **E**), Fisher exact test on normalized contingency data (**F**, **G**), one-sample *t* and Wilcoxon test in (**I**). Scale bars: (**A**, **H**) 12 μm and (**C**) 5 μm. Source data are available online for this figure.

**Figure EV4:**
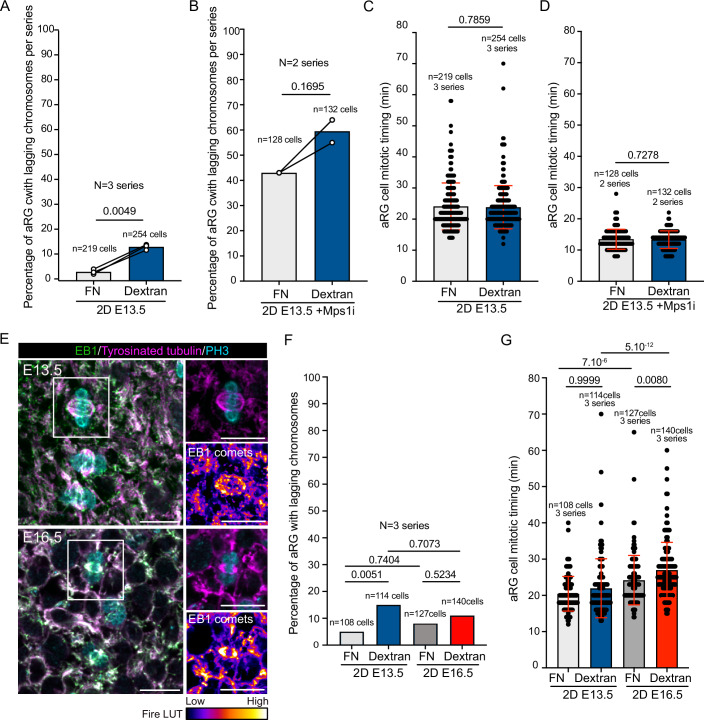
Analysis of 2RG primary cultures under increased compression imposed by dextran. (**A**, **B**) Bar graphs showing the mean frequency of lagging chromosomes and paired values for each series. (**C**, **D**) Scatter dot bar plot showing the mitotic timing. (**E**) on the left, pictures of cortical explants at the indicated developmental stage labeled with antibodies against EB1 (green), tyrosinated tubulin (magenta), and PH3 (Cyan). On the right, insets are shown with EB1 represented with Fire LUT. (**F**) Bar graph showing the percentage of cells with lagging chromosomes from cumulative analysis of three series at different neuro-developmental stages. (**G**) Scatter dot bar plot showing the corresponding mitotic timing. All data in (**C**, **D**, **G**) are presented as mean ± SD with a center line at the mean value and error bars indicating SD. Data were submitted to normality and lognormality tests for the choice of an appropriate statistical test. Statistical significance was assessed with paired *t* test (**A**, **B**) Mann–Whitney test (**C**, **D**) chi-square test on normalized contingency data (**F**) Kruskal–Wallis test (**G**). Scale bars: (**E**) 10 μm. Source data are available online for this figure.

Our findings support a causal relationship between biomechanical constraints, MT polymerization rates, and mitotic fidelity in aRG cells (Figs. 3D–M and EV3). We tested whether imposing constraints on 2D aRG cultures, which lack cell–cell contacts during mitosis, was sufficient to make them 3D-like. We used a biocompatible osmolyte of high molecular weight—dextran—that is diluted in the culture medium (Monnier et al, 2016; Dolega et al, 2021). Dextran exerted a mechanical pressure by counteracting the force required for mitotic cells to round up, as illustrated by their elongated shape at the interface with the substrate (Fig. 4A,B). Importantly, dextran did not alter their circular shape at the equator and therefore preserved the space required to accommodate the bipolar spindle and the metaphase plate (Fig. 4A) (Lancaster et al, 2013). Strikingly, however, this treatment was sufficient to increase spindle pole MT polymerization (Fig. 4C–E) and the frequency of lagging chromosomes in the absence (Figs. 4F and EV4A) or presence of Mps1i (Figs. 4G and EV4B) without affecting mitotic timing (Fig. EV4C,D). We concluded that aRGs dissociated from E13.5 brains cultured in 2D are responsive to mechanical constraints, which can also be referred to as mechanosensitivity.

We previously reported changes in spindle morphology when comparing brains from E13.5 and E16.5 neuro-developmental stages (Fig. EV1A,B) (Vargas-Hurtado et al, 2019). Co-labeling whole cerebral cortices at both stages for tubulin and EB1 comets as readouts of spindle MT dynamic properties in the native neuroepithelium showed obvious differences as well (Fig. EV4E).

To determine whether aRGs dissociated from E16.5 brains were comparable to E13.5 aRG in terms of mechanosensitivity, we imposed mechanical compression using dextran on 2D aRG cultures derived from E16.5 brains. We found only a 1.3-fold increase in the frequency of lagging chromosomes in E16.5 cultures, while E13.5 cultures exhibited a 3.4-fold increase (Figs. 4H,I and  EV4F,G).

These results indicate that when submitted to the same level of mechanical constraints as E13.5 mitotic aRGs, E16.5 aRGs are not as responsive and are therefore less mechanosensitive. We concluded that compressive stress imposed by 3D biomechanical constraints when cells undergo mitosis in multicellular environments dictates spindle pole MT polymerization capacity and chromosome segregation accuracy in a restricted developmental time window.

### Cortical actin is a conveyor of biomechanical compression in mitotic aRG cells

**Figure 5:**
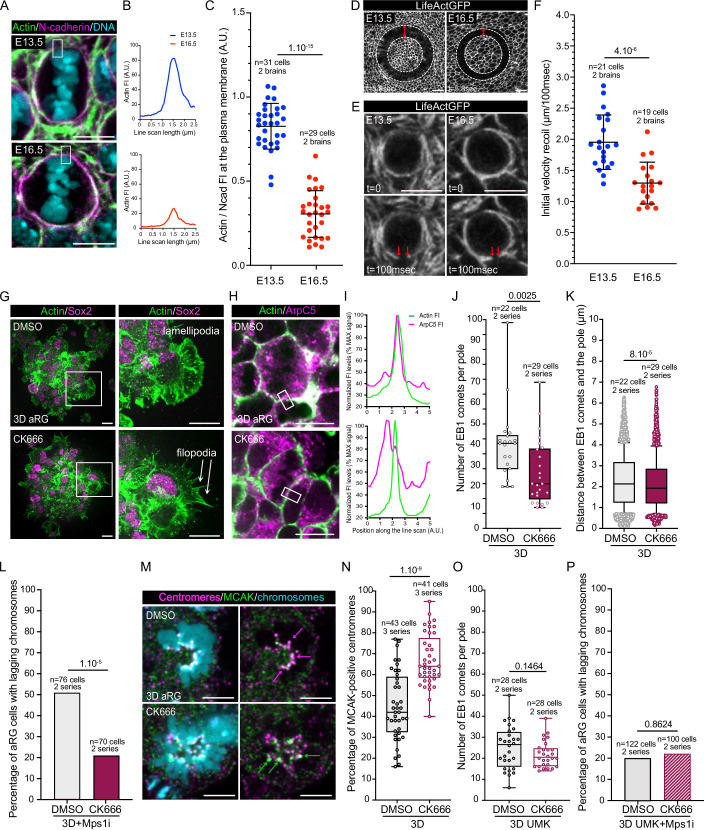
Cortical actin modulates MT polymerization rates and chromosome mis-segregation. (**A**) Pictures of aRG cells in mitosis from embryonic cerebral cortices of the indicated stages showing actin (green), N-cadherin (magenta), and DNA (cyan). The white rectangles in (**A**) point to the regions chosen for line scan analysis of actin FI provided in (**B**). (**C**) Scatter dot plot showing the FI level of actin over N-cadherin FI. (**D**) Pictures of the ventricular lining showing LifeAct-GFP boundaries (white dotted lines) after a circular shape laser cut. The red arrows show the distance after 5.8 s of recoil velocity. (**E**) Pictures of aRG mitotic cells from embryonic cerebral cortices of the indicated developmental stages expressing LifeAct-GFP to visualize the plasma membrane before (*t* = 0, upper panels) and 100 milliseconds (msec, lower panels) after laser cut. The two red arrows show the distance of recoil. (**F**) Scatter dot plot showing the initial velocity recoil. (**G**) On the left, images of 3D aRG cultures showing actin (green) and Sox2 (magenta) under the specified conditions. On the right, the same conditions show higher magnifications of the white squared regions on the left panels depicting lamellipodia in DMSO (top panels) and filopodia in CK666 (bottom panels) treatments. (**H**) Pictures of 3D aRG cultures showing ArpC5 localization (magenta) and actin (green). (**I**) Actin (green line) and Arpc5 (pink line) FI line scans of the plasma membrane in the regions depicted as white rectangles in (**H**). (**J**, **K**) Box and whiskers plots showing the number (J) and distance of individual EB1 comets traveled from the spindle pole (**K**) after 2 min of MT repolymerization. (**L**) Bar graph showing the percentage of cells with lagging chromosomes from the cumulative analysis of two series. (**M**) Pictures showing centromeres (magenta), MCAK (green), and chromosomes (cyan). Pink arrows point to centromeres devoid of MCAK signal, green arrows to centromeres enriched in MCAK. (**N**) Box and whiskers graph showing the percentage of centromeres loaded with MCAK. (**O**) Box and whiskers plot showing the number of EB1 comets polymerized per pole. (**P**) Bar graph showing the percentage of cells with lagging chromosomes from the cumulative analysis of two series. Data are presented as mean ± SD in (**C**, **F**). Median values are indicated by the middle line, and whiskers range from minimum to maximum values (**J**, **N**, **O**) or 10–90 percentiles (**K**). Data were submitted to normality and lognormality tests for the choice of an appropriate statistical test. Statistical significance was assessed with unpaired *t* test (**C**, **F**, **N**, **O**), Mann–Whitney test (**J**, **K**), and a Chi-square test (**L**, **P**). Scale bars: (**A**, **C**, **M**) 5 μm; (**D**, **E**, **G**, **H**) 10 μm. Source data are available online for this figure.

**Figure EV5:**
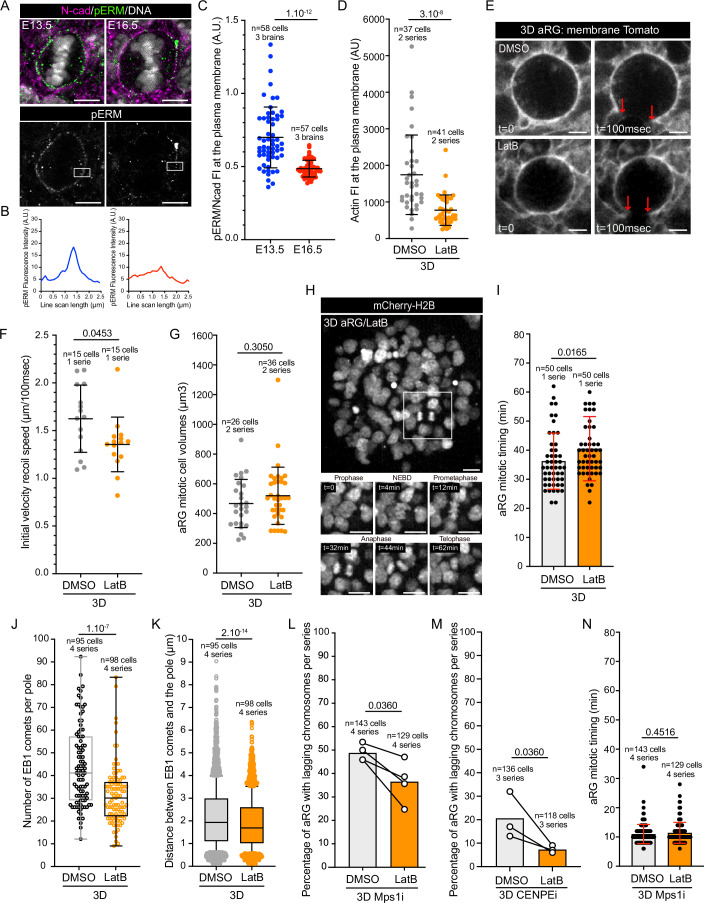
Impact of biomechanical constraint release induced by actin depolymerization. (**A**) Pictures of aRG mitotic cells from cerebral cortices at the indicated developmental stages labeled with antibodies that recognize phosphorylated ERM (pERM) (green and gray in the bottom), Ncad (magenta), and DNA (gray). The white rectangles point to the regions chosen for line scan analysis of pERM provided in (**B**). (**C**, **D**) Scatter dot plots showing cortical pERM over Ncad FI (**C**) or actin FI (**D**) in the conditions indicated. (**E**) Pictures of 3D aRG mitotic cells expressing membrane Tomato before (time 0, *t* = 0, left panels) or after (100 milliseconds, *t* = 100 msec, right panels) plasma membrane laser cuts. The red arrows show the position before and after the laser cut. (**F**) Scatter dot plots of the corresponding velocity recoil speeds. (**G**) Scatter dot plot of aRG mitotic cell volumes. (**H**) On the top, a picture of a neurosphere expressing m-Cherry-H2B during LatB treatment. On the bottom, the sequence of mitotic progression of a representative cell (white rectangle) from the neurosphere. (**I**) Scatter dot bar plot of aRG mitotic timing. (**J**, **K**) Box and whiskers plots displaying the cumulative number of EB1 comets per pole (**J**) and the distance between individual EB1 comets and the pole (**K**) after 2 min MT repolymerization. (**L**–**N**) Bar graphs depicting the mean percentage of cells with lagging chromosomes with data plotted per series (**L**, **M**) and corresponding mitotic timing (**N**) in the different conditions indicated. Data are represented as mean ± SD with a center line at the mean value and error bars indicating SD (**C**, **D**, **F**, **G**, **I**, **N**). Median values are indicated by the middle line, and whiskers range from minimum to maximum values (**J**) or 10 to 90 percentiles (**K**). Data were submitted to normality and lognormality tests for the choice of appropriate statistics. Statistical significance was assessed with Mann–Whitney tests or paired *t* test (only **L**, **M**). Scale bars: (**A**, **E**) 5 μm, (**H**) 10 μm. Source data are available online for this figure.

The mechanism for sensing compression as an indicator of neighboring cell density in 3D aRG in mitosis remains unknown. To identify possible cell indicators, we compared the levels of cortical actin, which contributes to mitotic cortical tension (Stewart et al, 2011; Chugh et al, 2017; Taubenberger et al, 2020) in E13.5 and E16.5 brains. We also characterized phosphorylated Ezrin, Radixin, and Moesin (pERM) proteins at the cell cortex as active pERM proteins link the plasma membrane and actin filaments to provide plasma membrane rigidity (Carreno et al, 2008; Kunda et al, 2008; Machicoane et al, 2014; Taubenberger et al, 2020). Interestingly, both cortical actin (Fig. 5A–C) and p-ERM levels (Fig. EV5A–C) were higher at E13.5 as compared to E16.5. To determine whether the observed differences in actin and pERM recruitment reflected changes in cortical membrane tension, we measured the initial velocity recoil speed after laser-cuts of aRG cells to obtain relative values of cortical tension (Farhadifar et al, 2007; Rujano et al, 2013; Krndija et al, 2019) (Fig. 5D–F). We used embryonic brains endogenously expressing LifeAct-GFP (Fig. 5D). We observed a higher velocity of membrane recoil, indicative of higher cortical membrane tension in E13.5 as compared to E16.5 brains (Fig. 5E,F).

We hypothesized that cortical actin may be influenced by compression levels, as cortical membrane tension is driven by the length of actin filaments in mitosis (Chugh et al, 2017). To test this possibility, we first used latrunculin B (LatB) at very low doses (50 nM) in an acute manner (1 h to 2.5 h of incubation). LatB binds actin monomers and therefore prevents actin filament polymerization (Spector et al, 1983). In 3D aRG configurations, LatB treatment resulted in a decrease in cortical actin recruitment (Fig. EV5D) and decreased cortical membrane tension after laser cut experiments (Fig. EV5E,F). In agreement with the literature, mitotic cell volumes remained unaltered after LatB treatment (Fig. EV5G) (Zlotek-Zlotkiewicz et al, 2015). Importantly, at the dose used and during the time-lapse recording (maximum 2.5 h), LatB treatment only slightly delayed anaphase onset, without affecting chromosome alignment or mitotic exit (Fig. EV5H,I). Altogether, our experimental conditions (dose and time of drug incubation) enabled us to focus our attention on mitotic entry and progression without adverse consequences of global actin depolymerization on aRG fitness. Analysis of EB1 comet behavior after MT depolymerization–repolymerization assays revealed a decrease in both the number and length of polymerizing MTs from the spindle poles (Fig. EV5J,K). Furthermore, in addition to decreasing polar MT polymerization, a decrease in the frequency of lagging chromosomes after acute treatments with Mps1i (Fig. EV5L) or CENPEi (Fig. EV5M) was also noticed, without impact on mitotic timing (Fig. EV5N).

**Figure EV6:**
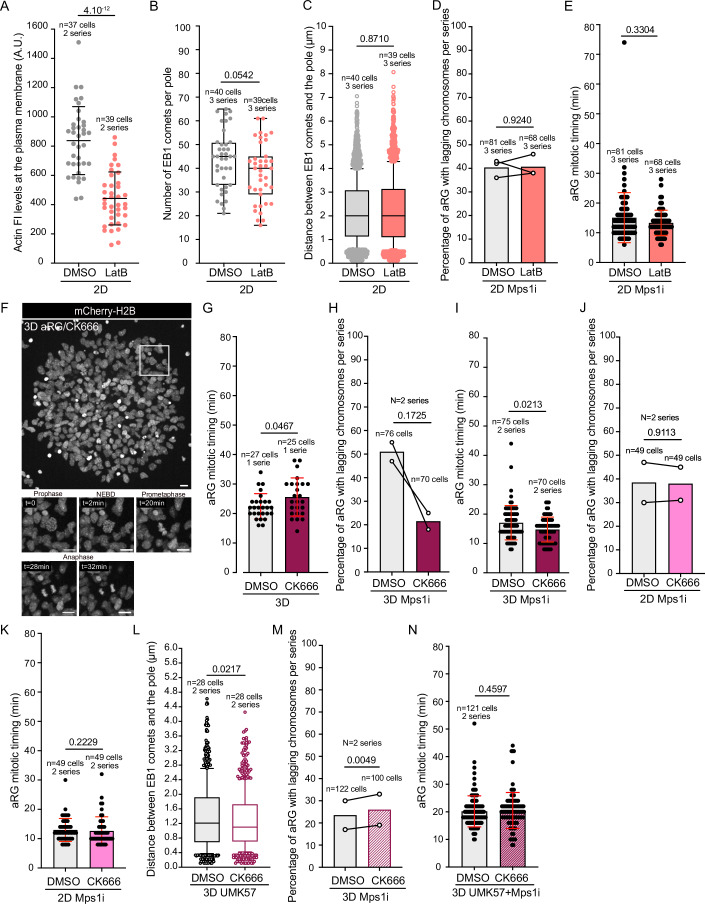
Analysis of 3D and 2D aRG primary cell cultures after cortical actin depolymerization. (**A**) Scatter dot plot showing cortical actin FI levels in 2D aRG cells. (**B**, **C**) Box and whiskers plots displaying cumulative number of EB1 comets polymerized per pole (**B**) and their distance from the pole (**C**) during 2 min MT repolymerization. (**D**, **E**) Bar graphs depicting the mean percentage of cells with lagging chromosomes with data plotted per series (**D**) and corresponding mitotic timing (**E**). (**F**) On top, a picture of a neurosphere expressing m-Cherry-H2B during CK666 treatment. On the bottom, the sequence of mitotic progression of a representative cell from the neurosphere (white rectangle). (**G**) Box and whisker plots showing mitotic timing. (**H**–**N**) Bar graphs depicting the mean percentage of cells with lagging chromosomes with data plotted per series (**H**, **J**, **M**) and corresponding mitotic timing (**I**, **K**, **N**) in the different conditions indicated. (**L**) Box and whiskers plot depicting the distance traveled by EB1 comets. Data are represented as mean ± SD with a center line at the mean value and error bars indicating SD (**A**, **E**, **G**, **I**, **K**, **N**). Median values are indicated by the middle line, and whiskers range from minimum to maximum values (**B**) or 10 to 90 percentiles (**C**, **L**). Data were submitted to normality and lognormality tests for the choice of appropriate statistics. Statistical significance was assessed with Mann–Whitney tests (**C**, **E**, **I**, **K**, **N**), unpaired *t* test (**A**, **B**, **L**), Welch’s test (**G**), ratio paired *t* test (**D**, **H**, **J**, **M**). Scale bars: (**F**) 10 μm. Source data are available online for this figure.

Importantly, cytoplasmic actin can contribute to MT dynamics, mitotic spindle assembly, and chromosome segregation (Mogessie and Schuh, 2017; Colin et al, 2018; Kita et al, 2019; Plessner et al, 2019). It was therefore important to test that cortical actin is a critical factor exclusively in 3D constrained settings. We therefore tested the consequences of actin depolymerization in 2D aRGs, which are not subjected to constraints imposed by neighbors during mitosis. Even if LatB treatment led to a decrease in cortical actin levels (Fig. EV6A), it did not impact the number or length of MTs polymerized from the poles (Fig. EV6B,C), nor the frequency of chromosome mis-segregation (Fig. EV6D,E), discarding possible side effects contributing to spindle assembly and/or chromosome segregation independently of cortical actin.

To further investigate the role of cortical actin as a conveyor of biomechanical compression in mitosis, we perturbed branched actin nucleation *via* an acute treatment with the Actin-Related Protein 2/3 (Arp2/3) complex inhibitor CK666 (Hetrick et al, 2012) at 100 μM for 1h-2.5 h. Similarly to LatB, we verified that mitotic progression was not significantly impaired regarding chromosome alignment, segregation, and mitotic timing (Fig. EV6F,G). As expected from a decrease in branched actin polymerization activity by Arp2/3 complex inhibition, a switch from lamellipodia to filopodia structures (Suraneni et al, 2012) at the interface between 3D aRG cultures and the culture support was noticed (Fig. 5G). Furthermore, we observed a delocalization of ArpC5, a subunit of the Arp2/3 complex, from the plasma membrane to the cytoplasm (Fig. 5H,I). Strikingly, both MT nucleation capacity from the spindle poles (Fig. 5J,K) and the rate of chromosome mis-segregation (Figs. 5L and EV6H,I), were significantly decreased in 3D aRG cells after Arp2/3 inhibition. Importantly, similarly to LatB, no such reduction in chromosome mis-segregation frequency was observed after CK666 treatment of 2D aRG cultures (Fig. EV6J,K).

Our results show that perturbing cortical cell properties—either by shortening actin filaments or decreasing the polymerization of branched actin—is sufficient to concurrently alter polar MT polymerization and the frequency of chromosome segregation errors exclusively in aRG cells under 3D constraints.

### Cortical actin acts upstream of MCAK to tune polar MT polymerization and modulate chromosome segregation errors

**Figure EV7:**
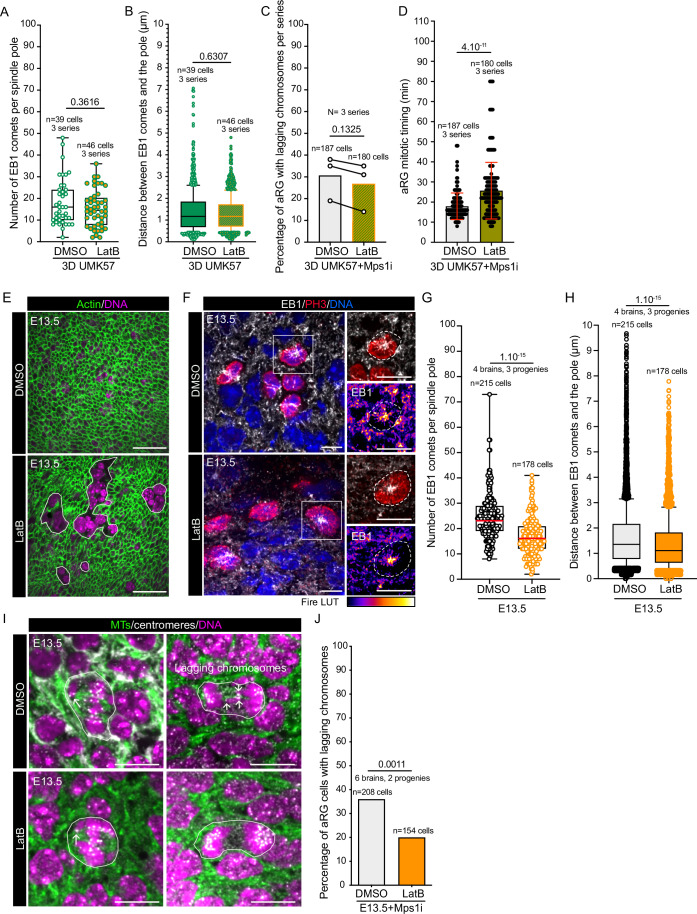
Analysis of the impact of biomechanical constraint release by actin depolymerization in E13.5 cerebral cortical explants. (**A**, **B**) Box and whiskers plots depicting the number (**A**) and the distance traveled (**B**) by EB1 comets in the indicated conditions. (**C**, **D**) Bar graph depicting the mean percentage of cells with lagging chromosomes, with data plotted per series and corresponding mitotic timing. (**E**) Pictures of E13.5 cerebral cortices showing actin (green) and DNA (magenta) at the level of the ventricular lining; white dashed lines highlight disruption of the neuroepithelium. (**F**) On the left, *en face* views of E13.5 cerebral cortices labeled with EB1 and PH3 antibodies shown in gray and magenta. DNA is in blue. On the right, insets show mitotic cells and EB1 comets depicted with the Fire LUT based on FI. (**G**, **H**) Box and whiskers plots showing EB1 comet number (**G**) and distances from spindle poles (**H**). (**I**) Pictures of E13.5 cerebral cortices labeled with antibodies recognizing MTs (green), centromeres (gray), and PH3 (purple). (**J**) Bar graph showing the percentage of cells with lagging chromosomes from the cumulative analysis of 6 brains out of 2 progenies. Data are presented as mean ± SD in (**D**). Median values are indicated by the middle line, and whiskers range from minimum to maximum values (**A**, **G**) or 10–90 percentiles (**B**, **H**). Data were submitted to normality and lognormality tests for the choice of an appropriate statistical test. Statistical significance was assessed with Mann–Whitney test (**A**, **B**, **D**, **G**, **H**), paired *t* test (**C**), and Chi-square test (**J**). Scale bars: (**E**) 20 μm; (**F**) left 5 μm and right 10 μm; (**I**) 10 μm. Source data are available online for this figure.

We next determined whether cortical actin and MCAK depolymerase activity act in the same pathway. We investigated MCAK kinetochore recruitment as a readout of its activity (Fig. EV3G,H) in 3D aRG cells after release of cortical actin tension upon CK666 treatment. We observed a 2.5-fold increase in the number of kinetochores containing MCAK (Fig. 5M,N), suggesting that releasing cortical tension is sufficient to modulate MCAK activity. A prediction of these results was that releasing cortical tension in the presence of sustained MCAK activity (in the presence of the UMK57 agonist) should not influence spindle pole MT polymerization nor chromosome mis-segregation anymore. Indeed, both parameters remained unchanged in such conditions (Figs. 5O,P and  EV6L,N). These results were further confirmed by combining UMK57 and LatB treatments in 3D aRG cultures (Fig. EV7A–D).

Together, these results suggest that biomechanical constraints on aRG mitosis are transmitted through cortical actin to MCAK, balancing polar MT polymerization and ensuring chromosome segregation fidelity.

### Releasing biomechanical constraints in vivo modulates astral MT polymerization and mitotic fidelity

**Figure 6:**
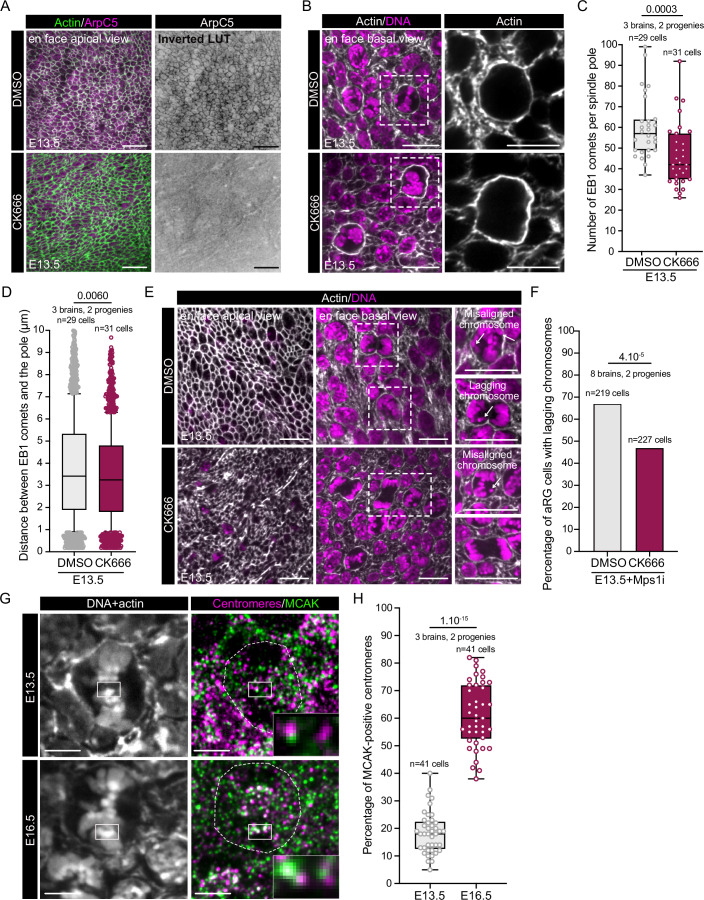
Releasing cortical tension in the embryonic cerebral cortex modulates spindle pole MT polymerization rates and mitotic accuracy through MCAK activity. (**A**) Pictures of E13.5 cortical explants labeled with antibodies that recognize ArpC5 (magenta and gray on the right) and actin (green). (**B**) Images of E13.5 cortical explants showing actin (gray) and DNA (magenta). (**C**, **D**) Box and whiskers plot showing the number (**C**) and the distance of individual EB1 comets (**D**) after 3 min of MT repolymerization. (**E**) Images of E13.5 cortical explants treated as indicated, showing actin (gray) and DNA (magenta) at different positions along the apical–basal axis as indicated. The white arrows point either to a mis-aligned chromosome in metaphase or a lagging chromosome in anaphase. (**F**) Bar graph showing the percentage of cells with lagging chromosomes from the cumulative analysis of 8 brains out of 2 progenies. (**G**) Pictures of aRGs in metaphase in cerebral cortices showing DNA and actin (gray), MCAK (green), and centromeres (magenta) at the indicated developmental stages. White boxes show higher magnifications of MCAK staining on a pair of kinetochores identified by centromere staining. Circular dotted lines highlight actin-stained metaphase cell boundaries. (**H**) Box and whiskers plot showing the percentage of MCAK-positive centromeres. Median values are indicated by the middle line, and whiskers range from minimum to maximum values (**C**, **H**) or 10–90 percentiles (**D**). Error bars indicate SEM, and the center corresponds to the mean. Data were submitted to normality and lognormality tests for the choice of an appropriate statistical test. Statistical significance was assessed with Mann–Whitney tests (**C**, **D**), unpaired *t* test (**H**), and Fisher’s exact test in (**F**). Scale bars: (**A**) 20 μm; (**B**) left 20 μm; right, 15 μm; (**E**) 10 μm and insets, 15 μm; (**G**) 5 μm. Source data are available online for this figure.

Our results show that cortical actin levels link polar MT polymerization and chromosome mis-segregation rates in mitotic aRG cells under constrained conditions imposed by neighboring cells. We next tested whether releasing biomechanical constraints in vivo would alleviate the levels of chromosome mis-segregation observed in the first period of neurogenesis -E13.5, when aRG crowding is elevated in the VZ and when aRG mitotic cells are mechanosensitive. Short-term incubation (less than 3 h) of cerebral cortex explants was sufficient to perturb cortical actin as illustrated by the disruption of the ventricular lining after LatB treatment (Fig. EV7E). Delocalization of the Arp2/3 complex from the plasma membrane (Fig. 6A) and changes in mitotic cell shape in the VZ were also observed after CK666 treatment (Fig. 6B), validating these two approaches to release cortical membrane tension in the brain in vivo in an acute manner. Interestingly, and similar to the experiments performed in vitro, the length and number of polar MTs after depolymerization/repolymerization assays were decreased (Figs. 6C,D and EV7F–H), along with the frequency of lagging chromosomes (Figs. 6E,F and EV7I,J). Three converging lines of evidence led us to predict elevated MCAK activity at E16.5: cortical membrane tension release increases MCAK activity in E13.5 3D aRG cultures; cortical actin levels are lower in mitotic aRGs at E16.5 in vivo; and E16.5 primary cultures show no sensitivity to mechanical compression in vitro. In line with this prediction, we used kinetochore-bound MCAK as a readout of its activity and found a threefold increase in prometaphase aRGs at E16.5 in comparison to E13.5 (Fig. 6G,H).

Our results show that the confluence of a crowded tissue environment and low MCAK levels found in E13.5 neurogenic stages tips the balance toward excessive MT polymerization and renders aRGs prone to chromosome mis-segregation.

## Discussion

In this study, we demonstrate that biomechanical constraints imposed by cell density critically affect chromosome segregation fidelity in the developing neocortex. These constraints arise when aRG cells are both spatially confined in space and restricted in the time window available to generate a progenitor pool of the appropriate size. Spatial confinement within the densely packed VZ is most pronounced during the first half of neurogenesis, when aRGs expand the pool of progenitors while giving rise to BPs. At this stage, they exhibit elevated levels of branched cortical actin associated with increased cortical stiffness during mitosis. As neurogenesis progresses, aRG daughter cells delaminate from the VZ, thereby reducing cellular crowding along with the incidence of chromosome segregation errors.

**Figure 7:**
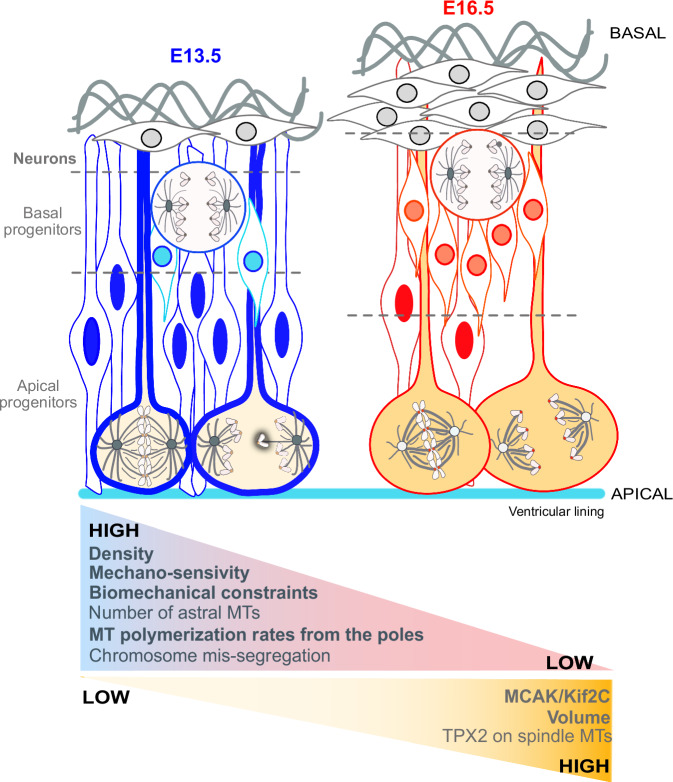
The population of aRG cells is mechanosensitive during the expansion phase. Model illustrating the differences between aRG cells at the two developmental stages characterized during this study. At E13.5, aRG cells, represented in blue, contain a thicker actin cortex than E16.5 aRG cells in red. Features of both cell types are depicted in the triangles below. Bold text illustrates the main findings of this study.

To link high cell density and chromosome segregation fidelity, we identified a connection between high cortical actin levels, increased MT polymerization rates at spindle poles, and reduced activity of the MT depolymerase MCAK/Kif2C (Fig. 7). The high MT polymerization rates are consistent with previous reports showing that aRG cells at mid-neurogenic stages possess mitotic spindles with elevated numbers and lengths of astral MTs (Mora-Bermúdez et al, 2014; Vargas-Hurtado et al, 2019). We further demonstrate that these features can be mimicked ex vivo by re-imposing confinement or tuning down after remodeling of cortical actin. Increased MCAK activity following cortical actin reorganization may serve at least two functions: modulating spindle pole MT polymerization capacity by regulating MT plus-end dynamics and contributing to error correction of MT- kinetochore attachments, as extensively described in multiple cell types (Stout et al, 2011; Tanenbaum et al, 2011; Walczak et al, 2016; van Heesbeen et al, 2017; McHugh and Welburn, 2023).

It is unclear why mitotic aRG cells display elevated cortical actin levels upon confinement. One plausible explanation is that, under the load of VZ mechanical constraint, enhanced cortical actin recruitment is required for cortical stiffening and aRG rounding-up, which are prerequisites for proper spindle assembly, positioning, and function (Matthews et al, 2012; Lancaster et al, 2013; Cadart et al, 2014; Lancaster and Baum, 2014; Machicoane et al, 2014; Pietro et al, 2016; Chugh et al, 2017; Taubenberger et al, 2020; Monster et al, 2021; Vadnjal et al, 2022). Another possibility is that aRG cells reorganize cortical actin in response to the level of mechanical constraint they face before mitotic entry. Accordingly, aRG nuclei in the G2 phase of the cell cycle must shuttle down to the apical side of the neuroepithelium under restricted lateral cell movements (Okamoto et al, 2013; Shimamura and Miyata, 2024).

Our results further indicate that aRGs in the VZ are more mechanosensitive before mid-neurogenic stages. Indeed, E13.5 aRG cells respond more strongly to equivalent mechanical constraints than E16.5 cells. This stage-specific mechanosensitivity may be required to fine-tune MT polymerization rates at spindle poles and maintain an appropriate astral MT network for accurate positioning of aRG divisions within the neuroepithelium. Failure in this regulation could lead to tissue dysplasia and disrupted cortical layering. However, this heightened mechanosensitivity and increased MT polymerization rate also elevate the likelihood of mitotic errors. Consistent with this, BPs that delaminate from the VZ and lose apicobasal polarity exhibit higher mitotic fidelity than aRGs when subjected to similar mitotic challenges. We exclude loss of polarity as the primary contributing factor. Indeed, dissociated aRGs cultured as 3D floating neurospheres retain mechanosensitivity, even if lacking their elongated shape or polarity cues.

Few studies have examined the role of tissue environment in influencing mitotic fidelity. It has recently been shown that epithelial cells show increased chromosome segregation errors after polarity loss outside of their native environment and in an integrin-dependent manner (Knouse et al, 2018). Our findings support the view that tissue properties can impact chromosome segregation fidelity, while a major contribution from cell crowding and cortical actin has to be considered instead of polarity in the developing mouse brain.

In pathological conditions where aRG mitosis is challenged, such as the ones found in MCPH patients carrying mutations in mitotic genes (Thornton and Woods, 2009; Kaindl et al, 2010; Jayaraman et al, 2018; Naveed et al, 2018; Farcy et al, 2023), increased frequency of chromosome mis-segregation may favor the generation of aneuploid daughter cells. Indeed, lagging chromosomes have 50% chances of segregating in the wrong daughter cell. When generated, abnormal genomes containing post-mitotic cells are efficiently removed from the cycling population by apoptosis or premature neuronal differentiation according to the literature in model organisms (Lizarraga et al, 2010; Marthiens et al, 2013; Gogendeau et al, 2015; Marjanović et al, 2015; Pilaz et al, 2016; Shi et al, 2019), therefore preventing the accumulation of aneuploid progenitors. In humans, this is still not known, but it remains an attractive hypothesis to explain the specific brain size reduction typical of MCPH patients.

Altogether, our findings suggest that during the initial period of cerebral cortex development, the combined demands of high proliferative activity and complex three-dimensional morphogenesis come at the cost of reduced mitotic accuracy. This trade-off occurs within a limited developmental window and does not produce overt defects in neocortex size or function at birth. However, this intrinsic vulnerability may be unmasked in conditions that impair mitotic spindle assembly or function, such as in human microcephaly-related disorders.

## Methods

**Table:** Reagents and tools table

Reagent/resource	Reference or source	Identifier or catalog number
**Experimental models**		
*Mus musculus* C57Bl6/N	Charles River (France)	N/A
*Mus musculus* R26-H2B-mCherry transgenic line	Curie Institute SPF inbred from Abe et al, 2011	N/A
*Mus musculus* LifeAct-GFP transgenic line	Curie Institute SPF inbred from Riedl et al, 2008	N/A
*Mus musculus* mT/mG transgenic line	Curie Institute SPF inbred from Muzumdar et al, 2007	N/A
**Recombinant DNA**		
pGFP-EB1	Addgene from Piehl and Cassimeris, 2003	17234/RRID: Addgene_17234
**Antibodies**		
Mouse IgG1 anti-α-tubulin (clone DM1α) 1/500	Sigma-Aldrich	T6199/RRID: AB_477583
Human anti-Centromere 1/100	Antibodies Incorporated	15-234-0001/RRID: AB_2687472
Mouse IgG1 anti-EB1 1/500	Cell Signaling	2164S/RRID: AB_2141765
Rabbit anti-MCAK/Kif2C 1/500	Abcam	ab71706/RRID: AB_1269382
Mouse anti-IgG1 anti-N-cadherin 1/250	BD Transduction Laboratories	610921/RRID: AB_398236
Rabbit anti-NuMA 1/500	Novus Biologicals	NB500-174/RRID: AB_10002562
Rabbit anti-phospho-ERM 1/500	Cell Signaling	3141/RRID: AB_330232
Rabbit anti-phospho-histone 3 (Ser10) 1/500	Sigma	H0412/RRID: AB_477043
Mouse IgG2a anti-p16Arc (ARPC5 subunit) 1/500	Synaptic System Antibodies	305011/RRID: AB_887896
Goat anti-Sox2 1/500	R&D	AF2018/RRID: AB_355110
Rat anti-tyrosinated tubulin (clone YL1/2) 1/100	Sigma	MAB1864/RRID: AB_2210391
Alexa Fluor Plus 647 Phalloidin 1/500	Thermo Fisher Scientific	A-30107
Alexa Fluor Plus 555 Phalloidin 1/500	Thermo Fisher Scientific	A-30106
Alexa Fluor 488 Phalloidin 1/500	Thermo Fisher Scientific	A-12379
DAPI 4’-6-diamino-2 phenylindole 1/500	Sigma	D1306
Goat anti-Rabbit IgG (H + L) Highly Cross-Adsorbed Secondary Antibody, Alexa Fluor 647 1/500	Thermo Fisher Scientific	A-21245/RRID: AB_2535813
Goat anti-Rabbit IgG (H + L) Highly Cross-Adsorbed Secondary Antibody, Alexa Fluor 546 IF 1/500	Thermo Fisher Scientific	A-11035/RRID: AB_2534093
Cy™5 AffiniPure® Donkey Anti-Rabbit IgG (H + L) IF 1/500	Jackson ImmunoResearch	709-175-149/RRID: AB_2340539
Goat anti-Rabbit IgG (H + L) Highly Cross-Adsorbed Secondary Antibody, Alexa Fluor 488 IF 1/500	Thermo Fisher Scientific	A-11034/RRID: AB_2576217
Goat anti-Mouse IgG1 Cross-Adsorbed Secondary Antibody, Alexa Fluor™ 488 IF 1/500	Thermo Fisher Scientific	A-21121/RRID: AB_2535764
Goat anti-Mouse IgG2a Cross-Adsorbed Secondary Antibody, Alexa Fluor™ 546 IF 1/500	Thermo Fisher Scientific	A-21133/RRID: AB_2535772
Donkey anti-Goat IgG (H + L) Highly Cross-Adsorbed Secondary Antibody, Alexa Fluor 488 IF 1/500	Thermo Fisher Scientific	A-32814/RRID: AB_2762838
Cy™3 AffiniPure® Donkey Anti-Human IgG (H + L) IF 1/500	Jackson ImmunoResearch	709-165-149/RRID: AB_2340535
**Chemicals, enzymes, and other reagents**		
Accutase	Sigma	A6964
Amaxa mouse NSC nucleofector kit	Lonza	VPG-1004
Antibiotic-antimycotic (100X)	Gibco	15240-062
B27 supplement without vitamin A	Gibco	15287-010
CK666	Tocris	3950
Dextran powder	Sigma	D5376
DMEM/HamF12 culture powder	Gibco	42400-028
DMSO anhydrous	Sigma	570672
EverBright mounting medium	Biotium	23001
Recombinant murine EGF	Peprotech	31509
Human FGF basic (FGF-2) 146a.a	Peprotech	100-18C
Fibronectin	Sigma	F1141
Glucose	Sigma	G7021
GSK923295	Selleckchem	S7090
Isoflurane	Baxter	DDG9621
Latrunculin B	Millipore	428020
Leibowitz’s L-15 medium	Thermo Fisher Scientific	11415064
MPI-0479605	Selleckchem	S7488
Ncad-Fc	BD	1388-NC
N2 supplement	Gibco	17502-048
Poly-D-Lysine hydrobromide	Sigma	P6407
Penicillin–streptomycin	Gibco	15070-063
Sodium bicarbonate	Sigma	S5761
Tridecafluoro-1,1,2,2-tetrahydrooctyl) trichlorosilane	ABCR	AB111444
10S Voltalef oil	VWR Chemicals	24627.188
**Software**		
Image J	Image J2 software V2.16.0/1.54p	RRID:SCR_003070 https://imagej.net/software/fiji/downloads
Affinity designer 2	Affinity	https://affinity.serif.com/fr/designer/.
Imaris software v.9.8.2	Bitplane	RRID:SCR_007370 https://imaris.oxinst.com/support/imaris-release-notes/9-9-0
GraphPad Prism version 10 for Mac	GraphPad Software	RRID:SCR_002798 www.graphpad.com.
Cellpose2	Pachitariu and Stringer, 2022	RRID:SCR_021716 http://www.cellpose.org
**Other**		
Cell culture inserts	Millipore	PICMORG50
PDL-coated glass-bottom dish	Greiner Bio-one	627870
Slice Anchor Kit for RC-22, SHD-22KIT	Warner Instruments	64-0263

### Animal husbandry and experimental procedures

Concerning animal care, we followed the European and French National Regulation for the Protection of Vertebrate Animals used for Experimental and other Scientific Purposes (Directive 2010/63; French Decree 2013-118). The project was authorized and benefited from the guidance of the Animal Welfare Body, Research Centre, Institut Curie. All mice were kept in the Institut Curie Specific Pathogen Free (SPF) animal facility for breeding. C57Bl6/N pregnant females were purchased from Charles River Laboratories (France). The transgenic mT/mG mouse line (stock number 007576, The Jackson Laboratory) (Muzumdar et al, 2007), LifeAct-GFP mouse line (Riedl et al, 2008), and Rosa26-mCherry-H2B mouse line (Abe et al, 2011) were inbred in our animal facility. For all pregnant females, the day of mating was referred to as 0.5 days of gestation (E0.5). Pregnant females were anesthetized by inhalation of isoflurane (Baxter, DDG9621) before being sacrificed by cervical dislocation. Embryos were collected after a cesarean section.

### Preparation of cortical explants, 3D or 2D apical radial glia (aRG) primary cultures

Dorsal telencephalons (referred to as cerebral cortices or cortical explants) of wild type C57Bl6/N mice were dissected at embryonic day 13.5 (E13.5) or 16.5 (E16.5) of neurogenesis. Dissections of mouse embryonic brain tissues for *en face view* analysis were described in detail in Rujano et al (2015). After dissection, cortical explants were incubated for 15 min at 37 °C under 5% CO_2_ in aRG culture medium (Vargas-Hurtado et al, 2019) before any other procedures. aRG culture medium contains DMEM/HamF12 medium (Gibco, 42400-028), supplemented with glucose (Sigma, G7021, 2.9 g/L), sodium bicarbonate (Sigma, S5761, 1.2 g/L), B27 supplement w/o vitamin A (Gibco, 12587-010; 2%), Fibroblast Growth Factor (Human FGF basic (FGF-2) 146a.a, Peprotech, 100-18 C, 20 ng/ml), Epidermal Growth Factor (Recombinant murine EGF, Peprotech, 31509, 20 ng/ml) and antibiotic-antimycotic 1% (Gibco, 15240-062).

For lateral view analysis, E13.5 brains were embedded in a solution of 3% low-melting agarose (Sigma, A9045) dissolved in dissection medium, containing DMEM/HamF12 medium (Gibco, 42400-028), supplemented with glucose (Sigma, G7021, 2.9 g/L), sodium bicarbonate (Sigma, S5761, 1.2 g/L) and penicillin-streptomycine (Gibco, 15070-063, 1%), and were maintained at 38 °C. Embryonic brains embedded in the agarose blocks were sectioned along the rostro-caudal axis with a vibratome (Leica VT1200S) in cold PBS to a thickness of 300 μm at a speed of 300 mm/s with an amplitude of 0.8 mm. Brain sections were deposited on cell culture inserts (Millipore, PICMORG50) and incubated at the air/liquid interface in the presence of 1 mL enriched slice culture medium. This slice culture medium is similar to the aRG culture medium supplemented with N2 supplement (Gibco 17502-048, 1%), decomplemented horse serum (5%), fetal bovine serum (5%), and penicillin–streptomycin (Gibco, 15070-063, 1%). Sections of cerebral cortices were incubated for 15 min at 37 °C under 5% CO_2_ before fixation or pharmacological treatments.

To establish primary cultures of apical radial glia (aRG), we have adapted the protocol described in Sun et al (2011). Cortical explants were dissociated into a single cell suspension by a combination of enzymatic treatment—incubation for 5 min at 37 °C with Accutase (Sigma, A6964)— and mechanical dissociation of mouse E13.5 brains. To establish 3D aRG cultures, cells were maintained as 3D floating aggregates—also called neurospheres—in non-adherent flasks at 37 °C under 5% CO_2_. Cells were cultured for 1 week in proliferation medium to enable maximal enrichment for Sox2-positive apical radial glia (aRG) before any experimental procedure (Vargas-Hurtado et al, 2019). They were kept in culture for 4 weeks.

Before using drugs and immunofluorescent approaches or time-lapse microscopy, 3D aRG cells were distributed on poly-D-Lysine-coated glass/plastic supports for 2H. For live imaging, neurospheres were plated on PDL-coated glass-bottom dishes (Greiner Bio-One, 627870) 2H before the start of the experiment. To establish 2D aRG cultures for perturbation assays, cells were dissociated the day before from neurospheres and plated on plastic/glass supports precoated sequentially for 2H at RT with poly-D-lysine hydrobromide (PDL, Sigma, P6407, 2 μg/cm^2^) and fibronectin (Sigma, F1141, 1 μg/cm^2^).

Transfections were performed with the Amaxa mouse NSC nucleofector kit (Lonza, VPG-1004), according to the manufacturer’s instructions. pGFP-EB1 was a gift from Lynne Cassimeris (RRID:Addgene_17234, http://n2t.net/addgene:17234) (Piehl and Cassimeris, 2003).

### Imposed mechanical stress on 2D aRG cells

To achieve bi-functionalized PDMS trenches, we adapted a technique previously developed by Yamada et al (2016) named in-mold patterning (IMP). Briefly, this technique uses a polydimethylsiloxane (PDMS) stamp of inverted trench polarity and a PDMS inker. The inker is incubated with 10% sodium dodecyl sulfate (SDS) solution for 15 min, rinsed with water, and further incubated with a 100 μg/ml fibronectin (FN) solution for at least 1 h. The stamp is exposed to an oxygen plasma, silanized using (Tridecafluoro-1,1,2,2-tetrahydrooctyl) trichlorosilane (ABCR, AB111444) for 30 min, and incubated with 10% SDS solution for 30 min before being dried with pressurized nitrogen. Lastly, the stamp is pressed for 3 min on the inker using a 30 g metal weight (the FN solution is pipetted just before, preventing the inker from drying before the stamping process) and removed. The inked stamp is further spin-coated with fresh PDMS at 500 rpm for 10 s, then 3000 rpm for 30 s (for a final theoretical thickness of about 75 μm). A plasma-activated glass coverslip is then placed on top of the PDMS layer as a physical support which, after reticulation at 70 °C overnight, will stick to the IMP. After peeling off the stamp, we obtain an easy-to-handle structured substrate with 10 µm deep trenches functionalized with FN at their bottom. The sample is next incubated for 1 h in a 4 µg/mL Ncad-Fc solution (BD, 1388-NC), deposited in the form of a drop on top of it, to functionalize the walls and the upper surface of the trenches. Importantly, we noticed that the Ncad-Fc did not cover the FN. At last, to remove Ncad-Fc from the surface of the IMP substrate and avoid cells to adhere elsewhere than inside the trenches, we used the stamp-off method originally proposed by Desai et al (2011), consisting of placing in conformal contact a flat, plasma-activated PDMS layer, over the IMP substrate for 30 s. The top Ncad-Fc is then transferred to this PDMS stamp-off object.

To impose mechanical stress on 2D aRG and perform live imaging, aRG cells dissociated from neurospheres were cultured on a PDL/fibronectin substrate and incubated for 1 h (fixed samples) or 2.5 h (live imaging) in the presence of a solution of dextran (Sigma, D5376) prepared at a final concentration of 80 mg/mL in the proliferation medium. Indeed, it has been proven (Dolega et al, 2021) that the osmotic pressure Π due to the osmolyte directly results in a solid stress σ of identical magnitude (σ = Π), under two conditions: (1) in the limit where the compressibility of the fluid is much lower than that of the solid phase. This condition is met here, as the bulk modulus of water is more than a thousand times larger than that of cells (Monnier et al, 2016), (2) when the osmolyte cannot be internalized by the cells, which is the case for dextran polymers of large molecular weight (Zlotek-Zlotkiewicz et al, 2015). Dextran of large molecular weight are long polymers. Thus, their osmotic pressure cannot simply be computed using van ‘t Hoff approximation, which is valid only for very diluted solutions. To determine the precise dilution required to obtain the desired pressure, we used the calibration curves published previously by (Vink, 1971; Bonnet-Gonnet et al, 1994).

Dissociated aRG cells were coated on a substrate of Ncadherin-Fc chimera (NcadFc, BD, 1388-NC) to mimic N-cadherin-dependent cell–cell contacts (Gavard et al, 2004). Ncad-Fc substrate was prepared by incubating PDL-coated supports with a solution of Ncad-Fc at 10μg/mL (BD, 1388-NC) prepared in Leibowitz’s L-15 medium (ThermoFisher Scientific, 11415064).

### Microtubule depolymerization–repolymerization experiments and drug-based assays

For microtubule (MT) depolymerization–repolymerization assays, cortical explants or aRG cultures were incubated for 1 h at 4 °C in proliferation medium to depolymerize MTs. Explants/cells were then incubated between 1.5 min and 3 min at 37 °C for MT repolymerization in the same medium before fixation for 2 min in −20 °C precooled methanol and immunofluorescent staining.

For drug treatments, the appropriate concentrations were predetermined in adherent aRG cultures. Chromosome misalignment and mis-segregation events were promoted using inhibitors of CENPE motor activity (CENPEi, GSK923295, Selleckchem, S7090) at 400 nM and Mps1 activity (Mps1i, MPI-0479605, Selleckchem, S7488) at 25 nM on 2D or 3D aRG cultures, 100 nM on E13.5 whole cerebral cortex explants, or 400 nM on E13.5 brain vibratome sections deposited on cell culture inserts. Actin filament polymerization was prevented using Latrunculin B (Millipore, 428020) at 50 nM. Branched actin polymerization was prevented using CK666 (Tocris Bioscience, 3950) at 100 μM on 2D or 3D aRG cultures and 200–400 μM on E13.5 cerebral cortex explants. MT dynamics were perturbed after treatment with the MCAK agonist UMK57 at 1 μM (Barroso-Vilares et al, 2020). When used in combination, CK666 was used at 50 μM, UMK57 at 0.25 μM, and Mps1i at 12.5 nM. All the drugs were diluted in dimethyl sulfoxide anhydrous (DMSO, Sigma, 276855). Explants/cells incubated with DMSO alone, diluted at equivalent concentrations (V/V), were used as negative controls. Cells were incubated for 1 h with the drugs before fixation and immunofluorescent staining or for 2.5 h during live imaging. Explants were incubated for 1 h with actin-depolymerizing drugs (LatB, CK666) or for 3 h with Mps1i before fixation.

### Immunofluorescence approaches

Cells/explants were fixed using different methods depending on the primary antibodies used. Cells or explants were either fixed for 15 or 30 min, respectively, in paraformaldehyde (PFA), diluted at 4% in PBS at room temperature, or fixed for 2 min in −20 °C precooled methanol. Samples were further incubated for 1 h in blocking solution (PBS, BSA 3%, Triton X-100 0.3%, Sodium azide 0.02%), followed by sequential overnight primary and secondary antibody incubations in the same buffer. Washes were performed with the blocking solution after each incubation. Cortical explants were mounted with the apical surface facing the glass coverslips for further analysis of apically localized mitotic progenitors, as described in Rujano et al (2015).

The following primary antibodies were used: mouse IgG1 anti-α-tubulin, dilution 1/500 (clone DM1α, Sigma, T6199, RRID:AB_477583), human anti-centromere, dilution 1/100 (Antibodies Incorporated, 15-234-0001, RRID:AB_2687472), mouse IgG1 anti-EB1, dilution 1/500 (Cell Signaling, 2164S, RRID:AB_2141765), rabbit anti-MCAK/Kif2C, dilution 1/500 (Abcam, ab71706, RRID:AB_1269382), mouse IgG1 anti-N-cadherin, dilution 1/500 (BD Transduction Laboratories, 610921, RRID:AB_398236), rabbit anti-NuMA, dilution 1/500 (Novus Biological, NB500-174, RRID:AB_10002562), rabbit anti-phospho-ERM, dilution 1/500 (Cell Signaling Technology, 3141, RRID:AB_330232), rabbit anti-Phospho-Histone 3 (Ser10, PH3), dilution 1/500 (Sigma, H0412, RRID:AB_477043), mouse IgG2a anti-p16Arc, dilution 1/500 (ARPC5 subunit, Synaptic System Antibodies, 305011, RRID:AB_887896), goat anti-Sox2, dilution 1/500 (R&D, AF2018, RRID:AB_355110), rat anti-tyrosinated tubulin, clone YL1/2 (Sigma, MAB1864, RRID:AB_2210391),. All secondary antibodies used were highly cross-absorbed: goat anti-rabbit Alexa Fluor 488 (Thermo Fisher Scientific, A-11034, RRID:AB_2576217), goat anti-rabbit Alexa Fluor 546 (Thermo Fisher Scientific, A-11035, RRID:AB_2534093), goat anti-rabbit Alexa Fluor 647 (Thermo Fisher Scientific, A-21245, RRID:AB_2535813), Cy5 AffiniPure Donkey Anti-Rabbit (Jackson Immunoresearch, 709-175-149, RRID:AB_2340539), goat anti-mouse IgG1 Alexa Fluor 488 (Thermo Fisher Scientific, A-21121, RRID:AB_2535764), goat anti-mouse IgG2a Alexa Fluor 546 (Thermo Fisher Scientific, A-21133, RRID:AB_2535772), donkey anti-goat IgG Alexa Fluor 488 (Thermo Fisher Scientific, A-32814, RRID:AB_2762838), Cy3 AffiniPure Donkey Anti-Human (Jackson Immunoresearch, 709-165-149, RRID:AB_2340535). For actin labeling, Alexa Fluor 488 Phalloidin (Thermo Fisher Scientific, A-12379), Alexa Fluor Plus 555 Phalloidin (Thermo Fisher Scientific, A-30106), and Alexa Fluor Plus 647 Phalloidin (Thermo Fisher Scientific, A-30107) were used at 1/500 dilution. 4’,6-diamidino-2-phenylindole (DAPI, Sigma, D1306) was used to label DNA at a concentration of 3 μg/ml and incubated overnight at RT at the same time as secondary antibodies. EverBrite Mounting medium (Biotium, 23001) was used for mounting.

### Cortical membrane factor recruitment analysis

For in vivo analysis, actin, phospho-ERM (p-ERM), and Nuclear Mitotic Apparatus (NuMA) protein levels at the cortical membrane were related to N-cadherin protein levels, which do not change between early and late neurogenesis. A 5 pixel width line scan fluorescence intensity analysis was performed for each marker along the plasma membrane identified with the N-cadherin staining. The mean intensity value was evaluated for each marker of interest and related to the mean intensity value of N-cadherin at the plasma membrane.

A similar 5-pixel-width line scan fluorescence intensity analysis was performed to evaluate actin fluorescence intensity (FI) levels at the plasma membrane after drug treatment in 3D or 2D aRG primary cultures. The mean intensity value was plotted for each mitosis.

### Evaluation of MCAK loading at kinetochores

To evaluate the fraction of kinetochores positive for MCAK, aRG cells in early prometaphase were selected for analysis. Images of *en face* views of each spindle from one pole to the other were considered for analysis. Cells were immunostained with the spindle pole marker γ-tubulin (not shown), a centromere marker to identify the site of kinetochore protein recruitment on chromosomes and count the number of structures of interest at any given position, and MCAK/Kif2C. Three z positions were considered for scoring per spindle, but only the mean value is provided per spindle. During mitosis, MCAK is distributed in the cytoplasm, at the spindle poles, and at kinetochores. To score the fraction of kinetochores positive for MCAK per spindle above the fluorescence intensity (FI) around the centromeric regions of interest, the FI evaluated on the spindle was systematically used to determine the minimal value of FI to be considered. All kinetochores with a visible MCAK signal after this treatment were scored as positive, whereas the absence of an MCAK signal scored the kinetochores as negative.

### aRG mitosis morphometrics analysis

To evaluate aRG mitotic cell volumes in a complex 3D environment and delineate cell contour, we took advantage of the cytoplasmic dispersion of the stem cell transcription factor Sox2 after nuclear envelope breakdown. For volume computation, the Cellpose algorithm (CellPose2, RRID:SCR_021716, http://www.cellpose.org) (Pachitariu and Stringer, 2022) was launched on the Sox2 channel on each slice separately in batch (Fig. EV1F). For each stack, a Fiji macro was developed where the user chooses the cells of interest by drawing a rectangle around them. The largest label obtained from Cellpose segmentation on each slice is stored in the ROI Manager. The user can manually refine the segmentation if needed, by modifying the segmentation on some slices or adding missing regions (especially for out-of-focus slices where the segmentation was less accurate). The volume is then computed using the sum of area multiplied by the interval between optical slices (Z step) (Fig. EV1F). The dedicated macro can be found in https://github.com/Anne-SophieMACE/MitoticCellVolume_EB1cometAnalysis.

3D reconstructions of mitotic cell volumes displayed in Fig. 1H were performed using the Imaris software (RRID:SCR_007370, http://www.bitplane.com/imaris/imaris).

### aRG nuclear shape segmentation and evaluation

To compare nuclear shapes along the apicobasal axis of the neuroepithelium, one z-section chosen in the dorso-lateral region of a cerebral cortex labeled with Dapi to stain DNA and Sox2 to identify the ventricular zone containing aRGs (Fig. 2A) was selected. Cellpose algorithm (CellPose2, RRID:SCR_021716, http://www.cellpose.org) (Pachitariu and Stringer, 2022) was launched on the Dapi channel to delineate nuclear shape. Labels obtained from Cellpose segmentation were stored in the ROI Manager. Sox2-positive area containing APs was drawn manually in order to delineate VZ from SVZ. Area of each region of interest (VZ, Sox2-positive and SVZ, Sox2 negative) was evaluated in proportion to the whole section area (Fig. 1C). The aspect ratios (as a proxy of nuclear shapes) between the nuclear width defined as the dimension along the ventricular lining and the height defined as the dimension along the apicobasal axis were calculated for each segmented nucleus and plotted in Fig. 2B. Aspect ratios were color-coded using the BAR plugin in Fiji and the Fire LUT settled between nuclear aspect ratio values of 0.3 and 1 (Fig. 2A). The dedicated macros can be found in https://github.com/Anne-SophieMACE/MitoticCellVolume_EB1cometAnalysis, labeled as cellpose on nuclei.

### Cortical membrane tension measurement by laser ablation

Embryonic cortical explants prepared from LifeAct-GFP (cortical actin endogenously labeled with GFP) expressing transgenic mice progeny were placed for *en face view* acquisitions with the apical surface down-facing the glass-bottom tissue culture dishes (Fluorodish, FD35-100, WPI) in 80 μL of 37 °C prewarmed medium. Explants were maintained in place during the procedure with a slice anchor (Slice Anchor Kit for RC-22, SHD-22KIT, 64-0263, Warner Instruments) to minimize sample drift, surrounded with 10S Voltalef oil (VWR Chemicals, 24627.188) to favor medium oxygenation and prevent evaporation during the whole procedure. 3D aRG cultures prepared from mT/mG (membrane-targeted tandem dimer Tomato (mT)) expressing transgenic mice progeny were plated on PDL-precoated glass-bottom four-compartment cell culture dishes (Greiner Bio-One, 627870). Tissues/cells were imaged using a two-photon laser-scanning microscope (LSM880, Carl Zeiss) in single photon mode (488 or 561 line), using a 63×/NA 1.4 OIL DICII PL APO objective. The cortical membrane was ablated using a Ti:Sapphire laser (Mai Tai DeepSee, Spectra Physics) set at 800 nm. The laser power was optimized to efficiently ablate the cortical membrane without creating cavitation or cell damage. A single Z-section was considered at the equator of each mitotic cell for laser cutting and acquisition. Images were acquired with a time interval δt = 100 ms.

Image quality was improved using the Fiji software Gaussian blur filter to improve signal-to-noise and increase cortical membrane localization accuracy. Cortical membrane tension was evaluated by measuring the initial velocity of recoil post-ablation, corresponding to the ratio of the size of the wound in μm over the time elapsed between laser cut and first image recording (based on measured δt).

### Image acquisitions on fixed samples and time-lapse recording

Images were acquired on a spinning disk confocal wide-field microscope (Gataca Systems, France). Based on a CSU-W1 (Yokogawa, Japan), the spinning head was mounted on an inverted Eclipse Ti2 (Nikon) microscope equipped with a motorized XY Stage (Nikon, Japan). Images were acquired through 100×1.4NA Plan-Apo (fixed samples) or 40×1.3NA Plan-Apo (live imaging) objectives with a sCMOS camera (Prime95B, Photometrics, USA) or a Kinetix camera (efficiency 95%). For acquisition of *en face* view spindle images (Fig. EV1A), images were acquired with a super-resolution module on a large field of view (Live-SR). Optical sectioning was achieved using a piezo stage (Nano-z series, Mad City Lab, USA). Gataca Systems’ laser bench was equipped with 405, 491, and 561 nm laser diodes, delivering 150 mW each, coupled to the spinning disk head through a single-mode fiber. Multi-dimensional acquisitions were performed using MetaMorph 7.10.1 software (MetaMorph Microscopy Automation and Image Analysis Software, RRID:SCR_002368, http://www.moleculardevices.com/Products/Software/Meta-Imaging-Series/MetaMorph.html). Image analyses were performed using free online Fiji software (Image J2 software, RRID:SCR_003070https://imagej.net/software/fiji/downloads).

For time-lapse recording and scoring of chromosome segregation errors, mCherry-H2B expressing aRG cells in 2D or 3D were plated on the appropriate substrates in glass-bottom four-compartment cell culture dishes (627870, Greiner Bio-One). Images were acquired every 2 min with 1 μm z intervals for a maximum of 20 z-stacks per position during 2.5 to 3 h after adding the drugs.

Mitotic timing was calculated as the time elapsed between nuclear envelope breakdown (NEBD) and anaphase onset. A mean mitotic timing between 20 and 30 min in the control aRG population was considered as a quality control criterion to select the primary cultures to be used for analysis. Any control aRG population displaying more than 30 min mitotic timing by mean was discarded from the analysis. The frequency of chromosome segregation errors in the population was scored as the number of anaphases among mitotic cells analyzed that display at least one isolated chromatid that lags between the two main masses of separated chromatids. We focused our attention on the two first time points (*t* = 4 min) after anaphase onset for live analysis and on anaphases with the two masses of chromosomes clearly separated for fixed samples. There is one chance out of two that the wrongly attached chromatid (merotelic attachment) missegregates into the daughter cells to generate aneuploidy. Whether in 3D or in 2D settings, all analyses were performed while keeping the 3D volume of the mitotic cell analyzed or after 3D projection using the Imaris software if required (when the cell was not appropriately oriented for analysis, for example).

Data were plotted using GraphPad Prism (version 10 for Mac, GraphPad software, RRID:SCR_002798, www.graphpad.com) and statistical analysis performed with the same software. Figures were mounted using Affinity Designer software (Affinity Designer, RRID:SCR_016952, https://affinity.serif.com/en-us/designer/).

### MT dynamics analysis after live imaging or MT depolymerization–repolymerization assays

For tracking of EB1-positive comets in time-lapse movies of mitotic cells, the spindle pole with the highest MT polymerization activity was chosen. Acquisitions were performed at a single z section during 1 min every 1 sec in prometaphase with a ×100 objective mounted on the spinning disk wide version microscope (Gataca Systems, France), described in the image acquisitions section. We chose for analysis spindle poles stable in z and positioned in *en face* views so that tracked comets were not lost because of *z* axis movement. For each movie, we chose randomly three time points during the one-minute recording. We tracked each EB1-positive comet arising from one single pole until its disappearance. We evaluated the number of comets arising from the pole at a given time point (MT polymerization rate) and the maximum distance they traveled before disappearing (evaluation of the MT polymerization persistence).

A semi-automated Fiji macro was developed to compute EB1-positive comet number, length, and distance traveled from the pole on fixed samples after MT depolymerization–repolymerization assays (Fig. EV3A). The analysis was performed on a 0.4 μm maximum *z*-projection (3 *z* sections acquired at intervals of 0.2 μm). The user is asked first to draw the pole and the area where comets should be detected. The comets are detected as local maxima using the Find Maxima Plugin in Fiji, with the prominence parameter chosen by the user to detect most comets; manual editing of comets is possible by the user. Using the ROI of the pole, the distance from each comet to the pole is easily computed with the Fiji Plugin “Distance Transform 3D”. For measuring comet length, we considered the fact that all comets propagate outward from the pole and that the length can be determined by analyzing the line passing through the pole and the comet center. Along this line, a Gaussian fit centered on the comet’s position is performed; the length of the comet is then defined as the FWHM (full width at half maximum), approximated by 2.355 sigma. Aberrant values or bad fit are excluded, and the user can again manually check and adjust comets whose size was not well approximated (for instance, comets rotated relative to the pole/comet line, or those too close to another comet). The dedicated macro can be found in https://github.com/Anne-SophieMACE/MitoticCellVolume_EB1cometAnalysis.

Experiments and quantifications were performed by different authors of the manuscript for transparency in terms of data reproducibility.

## Supplementary information

Peer Review File

Source data Fig. 1

Source data Fig. 2

Source data Fig. 3

Source data Fig. 4

Source data Fig. 5

Source data Fig. 6

Figure EV1 Source data

Figure EV2 Source data

Figure EV3 Source data

Figure EV4 Source data

Figure EV5 Source data

Figure EV6 Source data

Figure EV7 Source data

Expanded View Figures

## Data Availability

The computer codes produced in this study are available in the following databases: Modelling computer scripts : GitHub https://github.com/Anne-SophieMACE/MitoticCellVolume_EB1cometAnalysis. The source data of this paper are collected in the following database record: biostudies:S-SCDT-10_1038-S44318-026-00848-3.
