# Supplementary material for: Mechanical constraints regulate embryonic stem cell mitosis in the developing brain
Source: EMBO J. 2026 Jul 10;45(16):5622–56. doi: 10.1038/s44318-026-00848-3 (PMC13482099; doi:10.1038/s44318-026-00848-3)
Supplement: Supplementary file 1 [file 44318_2026_848_MOESM15_ESM.pdf]

Expanded View Figures

Figure EV1. Analysis of aRG cell density as neurogenesis progresses.

(**A**) Pictures of *en face* views of whole-mount cerebral cortical explants from the indicated neurogenic stages showing MTs in green and DNA in magenta on the left and MTs in green and NuMA in magenta on the right. Insets show higher magnifications of mitotic cells. Dotted white lines highlight cell boundaries. (**B**) Scatter dot plot showing the ratio between NuMA/N-Cad FI level at the plasma membrane. (**C**) Pictures of *en face* views of cortical explants from the indicated neurogenic stages showing N-Cad signal. (**D**, **E**) Scatter dot plots showing aRG cell apical process density (**D**) and area (**E**). (**F**) Images showing the methods used to quantify mitotic aRG cell volumes. Lateral (xz, left panel) and *en face* (xy, right panels) views of a Sox2-positive aRG mitosis. Dotted red lines indicate examples of measured areas at different z positions. All data in (**B**, **D**, **E**) are presented as mean ± SD with a center line at the mean value and error bars indicating SD. Data were submitted to normality and lognormality tests for the choice of an appropriate statistical test. Statistical significance was assessed with a Mann–Whitney test (**B**) and Welch's tests (**D**, **E**). Scale bars: (**A**) 10 µm; (**C**) 5 µm; (**F**) 10 µm. Source data are available online for this figure.

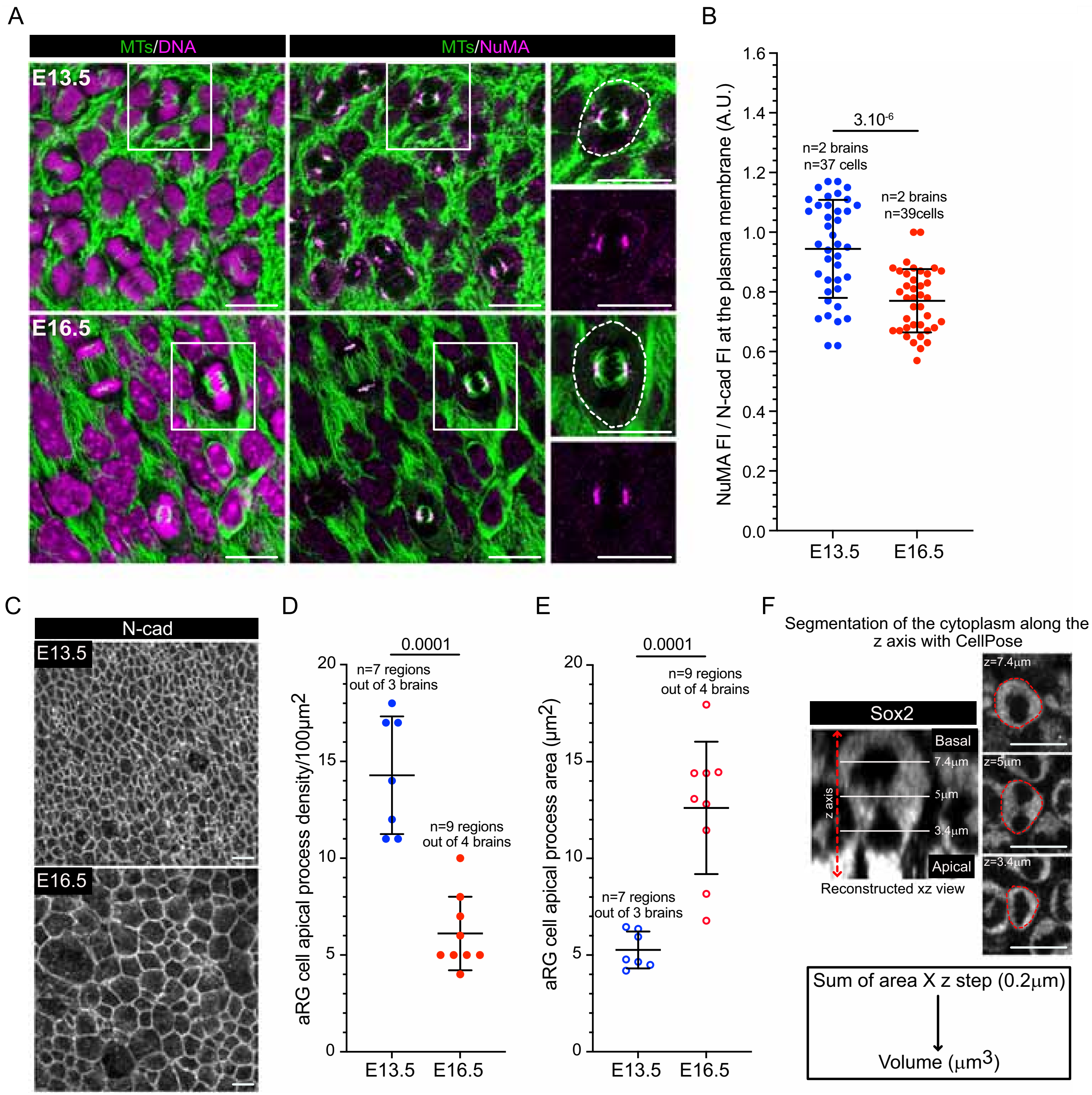
A
MTs/DNA
MTs/NuMA
E13.5
E16.5
B
3.10-6
n=2 brains
n=37 cells
n=2 brains
n=39cells
NuMA FI / N-cad FI at the plasma membrane (A.U.)
E13.5
E16.5
C
N-cad
E13.5
E16.5
D
0.0001
n=7 regions
out of 3 brains
n=9 regions
out of 4 brains
aRG cell apical process density/100µm2
E13.5
E16.5
E
0.0001
n=9 regions
out of 4 brains
n=7 regions
out of 3 brains
aRG cell apical process area (µm2)
E13.5
E16.5
F
Segmentation of the cytoplasm along the z axis with CellPose
Sox2
Basal
7.4µm
5µm
3.4µm
Apical
z axis
Reconstructed xz view
z=7.4µm
z=5µm
z=3.4µm
Sum of area X z step (0.2µm)
Volume (µm3)

 © The Author(s)

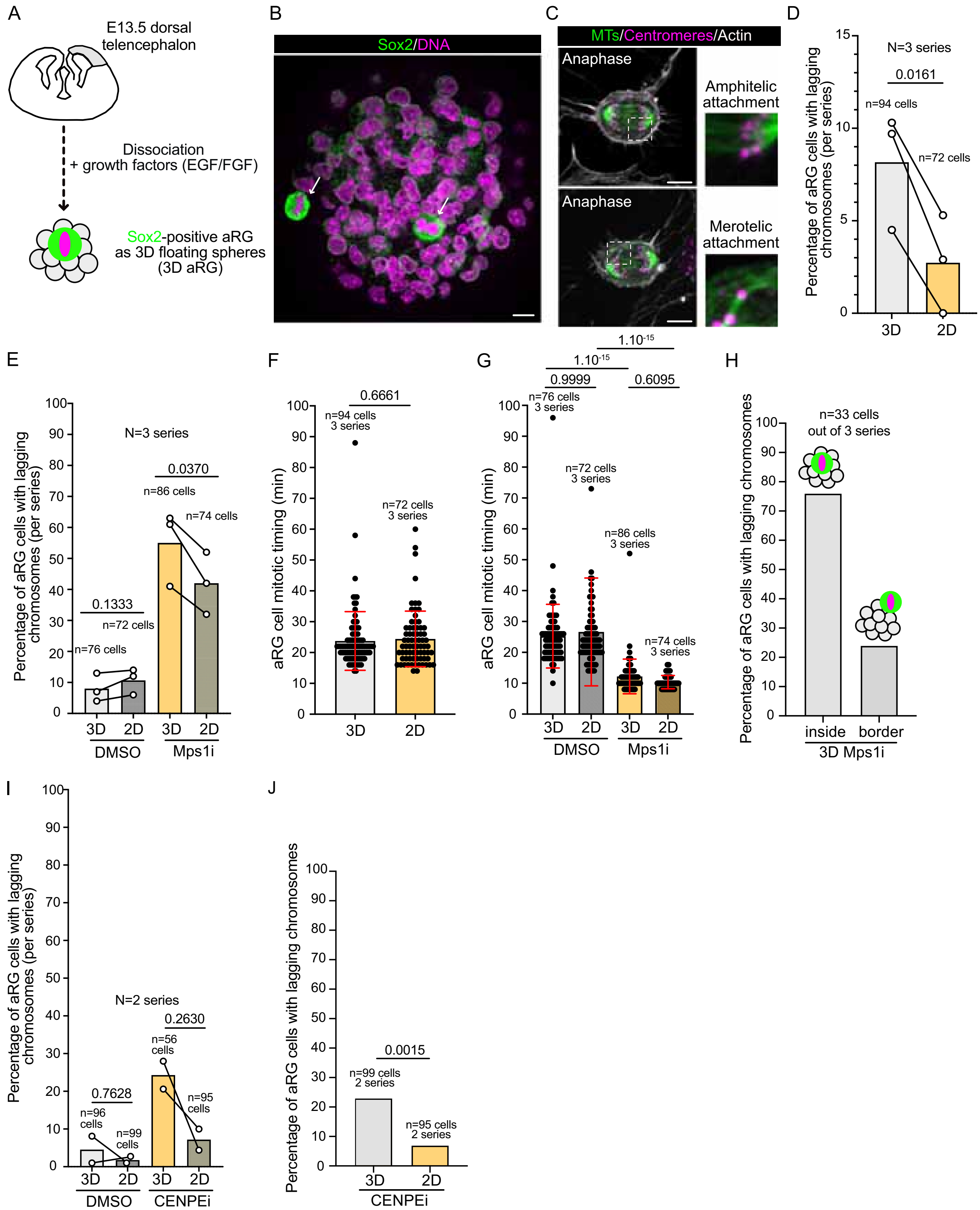
A
E13.5 dorsal telencephalon
Dissociation + growth factors (EGF/FGF)
Sox2-positive aRG as 3D floating spheres (3D aRG)
B
Sox2/DNA
C
MTs/Centromeres/Actin
Anaphase
Amphitelic attachment
Anaphase
Merotelic attachment
D
Percentage of aRG cells with lagging chromosomes (per series)
N=3 series
0.0161
n=94 cells
n=72 cells
3D
2D
E
Percentage of aRG cells with lagging chromosomes (per series)
N=3 series
0.0370
n=86 cells
n=74 cells
0.1333
n=72 cells
n=76 cells
3D 2D 3D 2D
DMSO Mps1i
F
aRG cell mitotic timing (min)
0.6661
n=94 cells 3 series
n=72 cells 3 series
3D 2D
G
aRG cell mitotic timing (min)
1.10-15
1.10-15
0.9999
0.6095
n=76 cells 3 series
n=72 cells 3 series
n=86 cells 3 series
n=74 cells 3 series
3D 2D 3D 2D
DMSO Mps1i
H
Percentage of aRG cells with lagging chromosomes
n=33 cells out of 3 series
inside border
3D Mps1i
I
Percentage of aRG cells with lagging chromosomes (per series)
N=2 series
0.2630
n=56 cells
n=95 cells
0.7628
n=96 cells
n=99 cells
3D 2D 3D 2D
DMSO CENPEi
J
Percentage of aRG cells with lagging chromosomes
0.0015
n=99 cells 2 series
n=95 cells 2 series
3D 2D
CENPEi

Figure EV2. Mitotic timing does not account for differences between 3D and 2D aRG cell primary cultures.

(**A**) Schematic representation of the protocol used to establish primary Sox2-positive aRG cultures from E13.5 dorsal telencephalon. (**B**) Picture of 3D aRG cells forming a floating sphere showing Sox2 (green) and DNA (magenta). (**C**) Pictures of aRG anaphase cells showing MTs (green), centromeres (magenta), and actin (gray) to illustrate amphitelic and merotelic attachments in the insets. (**D**, **E**) Bar graphs showing the mean frequency of lagging chromosomes and paired values for each series. (**F**, **G**) Scatter dot bar plots showing the mitotic timing. (**H**) Bar graph showing the frequency of cells with lagging chromosomes according to their position at the center or periphery of the neurosphere. (**I**, **J**) Bar graph showing the mean frequency of lagging chromosomes and paired values for each series (**I**) or the percentage of cells from cumulative analysis of the two series (**J**). All data in (**F**, **G**) are presented as mean ± SD with a center line at the mean value and error bars indicating SD. Data were submitted to normality and lognormality tests for the choice of appropriate statistics. Statistical significance was assessed with paired *t* test (**D**), ratio paired *t* tests (**E**, **I**), Mann–Whitney test (**F**), Kruskal–Wallis test (**G**), chi-square test on normalized contingency data in (**J**). Scale bars: (**B**) 10 µm and (**C**) 5 µm. Source data are available online for this figure.

 © The Author(s)

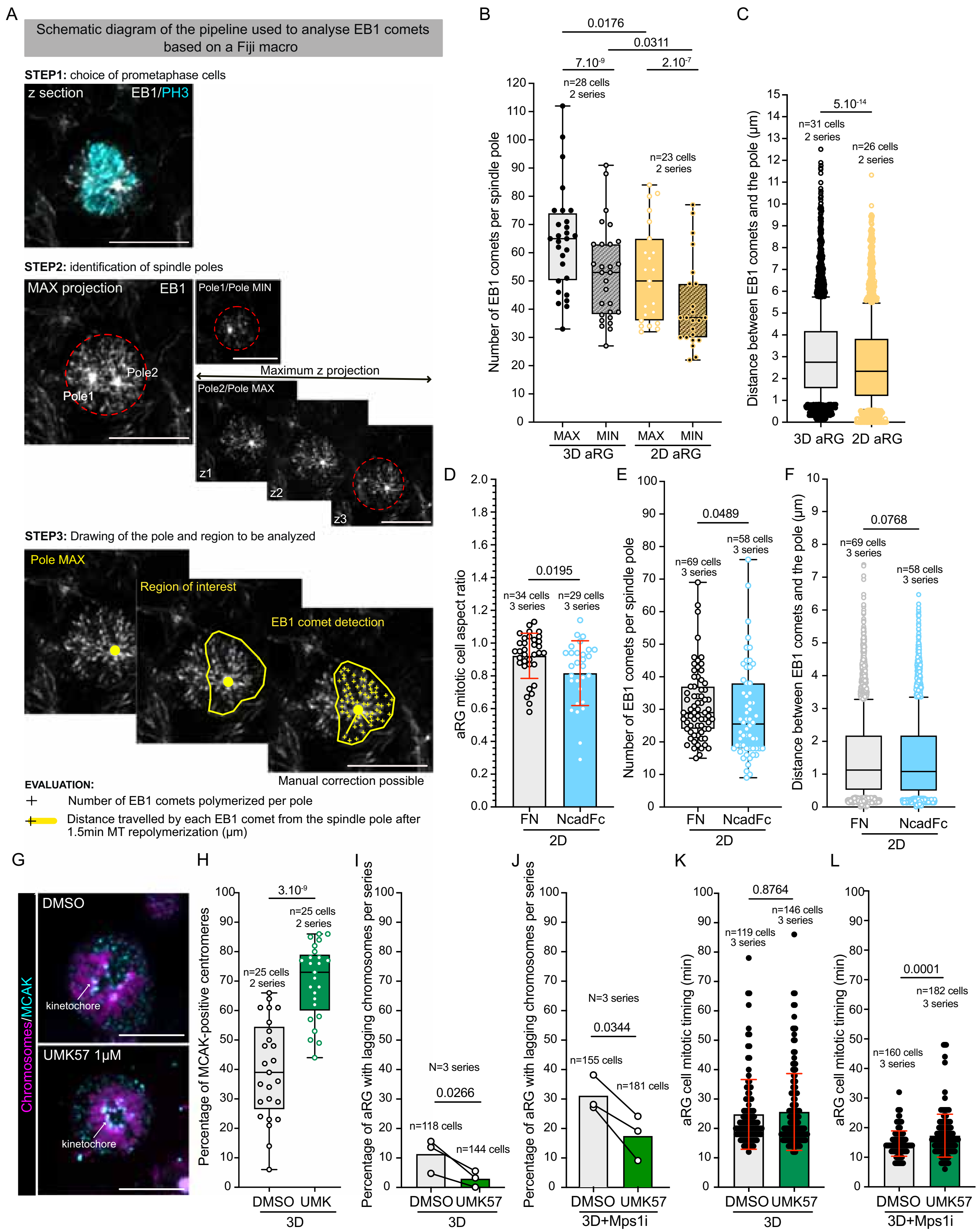
A
Schematic diagram of the pipeline used to analyse EB1 comets based on a Fiji macro
STEP1: choice of prometaphase cells
z section
EB1/PH3
STEP2: identification of spindle poles
MAX projection
EB1
Pole1/Pole MIN
Pole2
Pole1
Maximum z projection
Pole2/Pole MAX
z1
z2
z3
STEP3: Drawing of the pole and region to be analyzed
Pole MAX
Region of interest
EB1 comet detection
Manual correction possible
EVALUATION:
Number of EB1 comets polymerized per pole
Distance travelled by each EB1 comet from the spindle pole after 1.5min MT repolymerization (µm)
B
Number of EB1 comets per spindle pole
0.0176
0.0311
7.10-9
2.10-7
n=28 cells
2 series
n=23 cells
2 series
MAX
MIN
MAX
MIN
3D aRG
2D aRG
C
Distance between EB1 comets and the pole (µm)
5.10-14
n=31 cells
2 series
n=26 cells
2 series
3D aRG
2D aRG
D
aRG mitotic cell aspect ratio
0.0195
n=34 cells
3 series
n=29 cells
3 series
FN
NcadFc
2D
E
Number of EB1 comets per spindle pole
0.0489
n=69 cells
3 series
n=58 cells
3 series
FN
NcadFc
2D
F
Distance between EB1 comets and the pole (µm)
0.0768
n=69 cells
3 series
n=58 cells
3 series
FN
NcadFc
2D
G
Chromosomes/MCAK
DMSO
kinetochore
UMK57 1µM
kinetochore
H
Percentage of MCAK-positive centromeres
3.10-9
n=25 cells
2 series
n=25 cells
2 series
DMSO
UMK
3D
I
Percentage of aRG with lagging chromosomes per series
N=3 series
0.0266
n=118 cells
n=144 cells
DMSO
UMK57
3D
J
Percentage of aRG with lagging chromosomes per series
N=3 series
0.0344
n=155 cells
n=181 cells
DMSO
UMK57
3D+Mps1i
K
aRG cell mitotic timing (min)
0.8764
n=146 cells
3 series
n=119 cells
3 series
DMSO
UMK57
3D
L
aRG cell mitotic timing (min)
0.0001
n=182 cells
3 series
n=160 cells
3 series
DMSO
UMK57
3D+Mps1i

◀ **Figure EV3. Analysis of MT depolymerization–repolymerization assays in 2D and 3D aRG cultures.**

(**A**) Schematic diagram of the analysis of EB1 comets after MT depolymerization–repolymerization assays. The first step consists of choosing a prometaphase cell thanks to PH3 labeling. In the second step, the pole to be quantified is chosen, taking into consideration the EB1 signal. A maximum projection containing three individual z-stacks is used. The region to be considered is manually segmented. Through the dedicated macro, the parameters extracted are the number of EB1 comets and the distance traveled from the pole. (**B**, **C**) Box and whiskers plots showing the cumulative number of EB1 comets (**B**) and distance traveled (**C**) per pole during 1.5 min MT repolymerization. (**D**) Scatter dot bar plot showing the aspect ratios of 2D aRG cells plated on different substrates. (**E**, **F**) Box and whiskers graphs showing the mean number of EB1 comets (**E**) and the distance traveled from the pole (**F**). (**G**) Pictures of aRG cells treated as indicated, labeled with MCAK antibodies (cyan). Chromosomes are shown in magenta. (**H**) Box and whiskers plot showing the percentage of kinetochores positive for MCAK per cell. (**I**, **J**) Bar graphs showing the mean frequency of lagging chromosomes and paired values for each series. (**K**, **L**) Scatter dot bar plot showing the mitotic timing. Data are mean ± SD in (**D**, **K**, **L**). Median values are indicated by the middle line, and whiskers range from minimum to maximum values (**B**, **E**, **H**) or 10–90 percentiles (**C**, **F**). Data were submitted to normality and lognormality tests for the choice of appropriate statistics. Statistical significance was assessed with the Wilcoxon matched-pairs signed rank test (**B**), unpaired *t* test (**H**), Mann–Whitney tests (**C**, **D**, **E**, **F**, **K**, **L**), ratio paired *t* test (**I**), and paired *t* test (**J**). Scale bars: (**A**) 12 µm and (**G**) 10 µm. Source data are available online for this figure.

 © The Author(s)

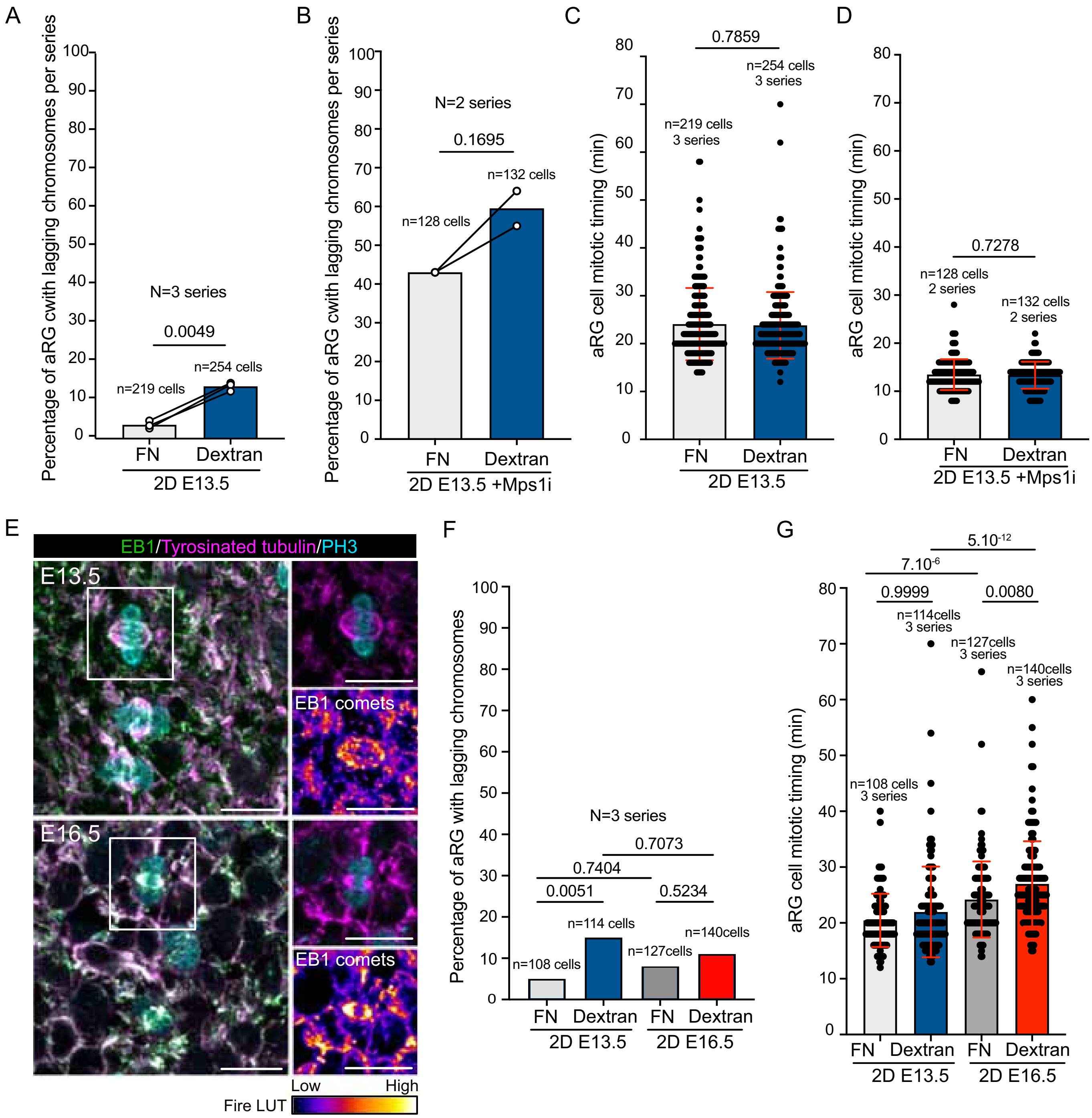

Figure EV4. Analysis of 2RG primary cultures under increased compression imposed by dextran.

(**A**, **B**) Bar graphs showing the mean frequency of lagging chromosomes and paired values for each series. (**C**, **D**) Scatter dot bar plot showing the mitotic timing. (**E**) on the left, pictures of cortical explants at the indicated developmental stage labeled with antibodies against EB1 (green), tyrosinated tubulin (magenta), and PH3 (Cyan). On the right, insets are shown with EB1 represented with Fire LUT. (**F**) Bar graph showing the percentage of cells with lagging chromosomes from cumulative analysis of three series at different neuro-developmental stages. (**G**) Scatter dot bar plot showing the corresponding mitotic timing. All data in (**C**, **D**, **G**) are presented as mean ± SD with a center line at the mean value and error bars indicating SD. Data were submitted to normality and lognormality tests for the choice of an appropriate statistical test. Statistical significance was assessed with paired *t* test (**A**, **B**) Mann–Whitney test (**C**, **D**) chi-square test on normalized contingency data (**F**) Kruskal–Wallis test (**G**). Scale bars: (**E**) 10 µm. Source data are available online for this figure.

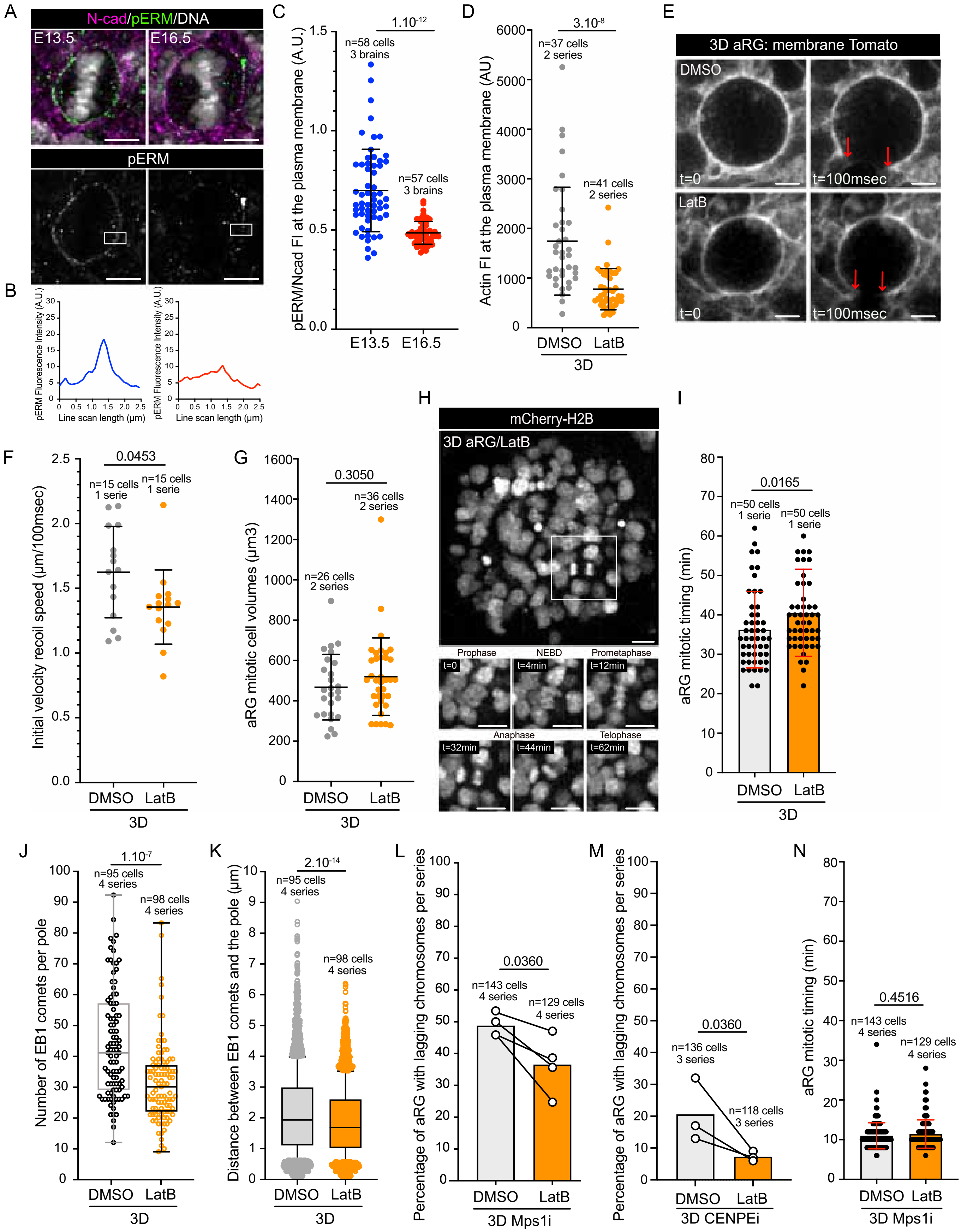
A
N-cad/pERM/DNA
E13.5
E16.5
pERM
B
pERM Fluorescence Intensity (A.U.)
Line scan length (µm)
C
pERM/Ncad FI at the plasma membrane (A.U.)
1.10-12
n=58 cells
3 brains
n=57 cells
3 brains
E13.5
E16.5
D
Actin FI at the plasma membrane (AU)
3.10-8
n=37 cells
2 series
n=41 cells
2 series
DMSO
LatB
3D
E
3D aRG: membrane Tomato
DMSO
LatB
t=0
t=100msec
F
Initial velocity recoil speed (µm/100msec)
0.0453
n=15 cells
1 serie
n=15 cells
1 serie
DMSO
LatB
3D
G
aRG mitotic cell volumes (µm3)
0.3050
n=26 cells
2 series
n=36 cells
2 series
DMSO
LatB
3D
H
mCherry-H2B
3D aRG/LatB
Prophase
NEBD
Prometaphase
Anaphase
Telophase
t=0
t=4min
t=12min
t=32min
t=44min
t=62min
I
aRG mitotic timing (min)
0.0165
n=50 cells
1 serie
n=50 cells
1 serie
DMSO
LatB
3D
J
Number of EB1 comets per pole
1.10-7
n=95 cells
4 series
n=98 cells
4 series
DMSO
LatB
3D
K
Distance between EB1 comets and the pole (µm)
2.10-14
n=95 cells
4 series
n=98 cells
4 series
DMSO
LatB
3D
L
Percentage of aRG with lagging chromosomes per series
0.0360
n=143 cells
4 series
n=129 cells
4 series
DMSO
LatB
3D Mps1i
M
Percentage of aRG with lagging chromosomes per series
0.0360
n=136 cells
3 series
n=118 cells
3 series
DMSO
LatB
3D CENPEi
N
aRG mitotic timing (min)
0.4516
n=143 cells
4 series
n=129 cells
4 series
DMSO
LatB
3D Mps1i

 © The Author(s)

Figure EV5. Impact of biomechanical constraint release induced by actin depolymerization.

(**A**) Pictures of aRG mitotic cells from cerebral cortices at the indicated developmental stages labeled with antibodies that recognize phosphorylated ERM (pERM) (green and gray in the bottom), Ncad (magenta), and DNA (gray). The white rectangles point to the regions chosen for line scan analysis of pERM provided in (**B**). (**C**, **D**) Scatter dot plots showing cortical pERM over Ncad FI (**C**) or actin FI (**D**) in the conditions indicated. (**E**) Pictures of 3D aRG mitotic cells expressing membrane Tomato before (time 0, $t = 0$, left panels) or after (100 milliseconds, $t = 100$ msec, right panels) plasma membrane laser cuts. The red arrows show the position before and after the laser cut. (**F**) Scatter dot plots of the corresponding velocity recoil speeds. (**G**) Scatter dot plot of aRG mitotic cell volumes. (**H**) On the top, a picture of a neurosphere expressing m-Cherry-H2B during LatB treatment. On the bottom, the sequence of mitotic progression of a representative cell (white rectangle) from the neurosphere. (**I**) Scatter dot bar plot of aRG mitotic timing. (**J**, **K**) Box and whiskers plots displaying the cumulative number of EB1 comets per pole (**J**) and the distance between individual EB1 comets and the pole (**K**) after 2 min MT repolymerization. (**L–N**) Bar graphs depicting the mean percentage of cells with lagging chromosomes with data plotted per series (**L**, **M**) and corresponding mitotic timing (**N**) in the different conditions indicated. Data are represented as mean ± SD with a center line at the mean value and error bars indicating SD (**C**, **D**, **F**, **G**, **I**, **N**). Median values are indicated by the middle line, and whiskers range from minimum to maximum values (**J**) or 10 to 90 percentiles (**K**). Data were submitted to normality and lognormality tests for the choice of appropriate statistics. Statistical significance was assessed with Mann–Whitney tests or paired *t* test (only **L**, **M**). Scale bars: (**A**, **E**) 5 µm, (**H**) 10 µm. Source data are available online for this figure.

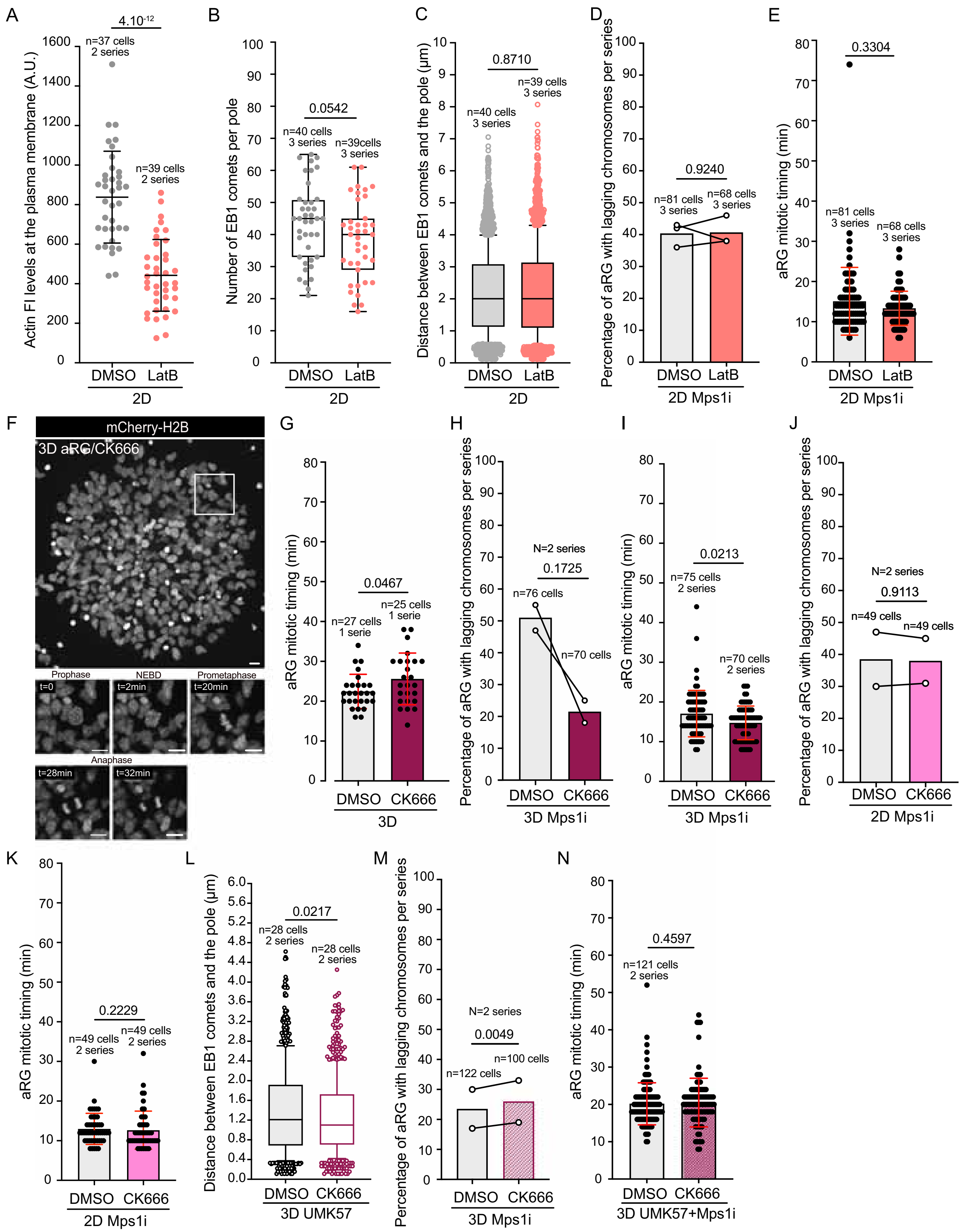
A
4.10-12
n=37 cells
2 series
n=39 cells
2 series
Actin FI levels at the plasma membrane (A.U.)
DMSO LatB
2D
B
0.0542
n=40 cells
3 series
n=39cells
3 series
Number of EB1 comets per pole
DMSO LatB
2D
C
0.8710
n=40 cells
3 series
n=39 cells
3 series
Distance between EB1 comets and the pole (µm)
DMSO LatB
2D
D
0.9240
n=81 cells
3 series
n=68 cells
3 series
Percentage of aRG with lagging chromosomes per series
DMSO LatB
2D Mps1i
E
0.3304
n=81 cells
3 series
n=68 cells
3 series
aRG mitotic timing (min)
DMSO LatB
2D Mps1i
F
mCherry-H2B
3D aRG/CK666
Prophase
NEBD
Prometaphase
t=0
t=2min
t=20min
Anaphase
t=28min
t=32min
G
0.0467
n=27 cells
1 serie
n=25 cells
1 serie
aRG mitotic timing (min)
DMSO CK666
3D
H
N=2 series
0.1725
n=76 cells
n=70 cells
Percentage of aRG with lagging chromosomes per series
DMSO CK666
3D Mps1i
I
0.0213
n=75 cells
2 series
n=70 cells
2 series
aRG mitotic timing (min)
DMSO CK666
3D Mps1i
J
N=2 series
0.9113
n=49 cells
n=49 cells
Percentage of aRG with lagging chromosomes per series
DMSO CK666
2D Mps1i
K
0.2229
n=49 cells
2 series
n=49 cells
2 series
aRG mitotic timing (min)
DMSO CK666
2D Mps1i
L
0.0217
n=28 cells
2 series
n=28 cells
2 series
Distance between EB1 comets and the pole (µm)
DMSO CK666
3D UMK57
M
N=2 series
0.0049
n=122 cells
n=100 cells
Percentage of aRG with lagging chromosomes per series
DMSO CK666
3D Mps1i
N
0.4597
n=121 cells
2 series
aRG mitotic timing (min)
DMSO CK666
3D UMK57+Mps1i

 © The Author(s)

Figure EV6. Analysis of 3D and 2D aRG primary cell cultures after cortical actin depolymerization.

(**A**) Scatter dot plot showing cortical actin FI levels in 2D aRG cells. (**B**, **C**) Box and whiskers plots displaying cumulative number of EB1 comets polymerized per pole (**B**) and their distance from the pole (**C**) during 2 min MT repolymerization. (**D**, **E**) Bar graphs depicting the mean percentage of cells with lagging chromosomes with data plotted per series (**D**) and corresponding mitotic timing (**E**). (**F**) On top, a picture of a neurosphere expressing m-Cherry-H2B during CK666 treatment. On the bottom, the sequence of mitotic progression of a representative cell from the neurosphere (white rectangle). (**G**) Box and whisker plots showing mitotic timing. (**H**–**N**) Bar graphs depicting the mean percentage of cells with lagging chromosomes with data plotted per series (**H**, **J**, **M**) and corresponding mitotic timing (**I**, **K**, **N**) in the different conditions indicated. (**L**) Box and whiskers plot depicting the distance traveled by EB1 comets. Data are represented as mean ± SD with a center line at the mean value and error bars indicating SD (**A**, **E**, **G**, **I**, **K**, **N**). Median values are indicated by the middle line, and whiskers range from minimum to maximum values (**B**) or 10 to 90 percentiles (**C**, **L**). Data were submitted to normality and lognormality tests for the choice of appropriate statistics. Statistical significance was assessed with Mann–Whitney tests (**C**, **E**, **I**, **K**, **N**), unpaired *t* test (**A**, **B**, **L**), Welch's test (**G**), ratio paired *t* test (**D**, **H**, **J**, **M**). Scale bars: (**F**) 10 µm. Source data are available online for this figure.

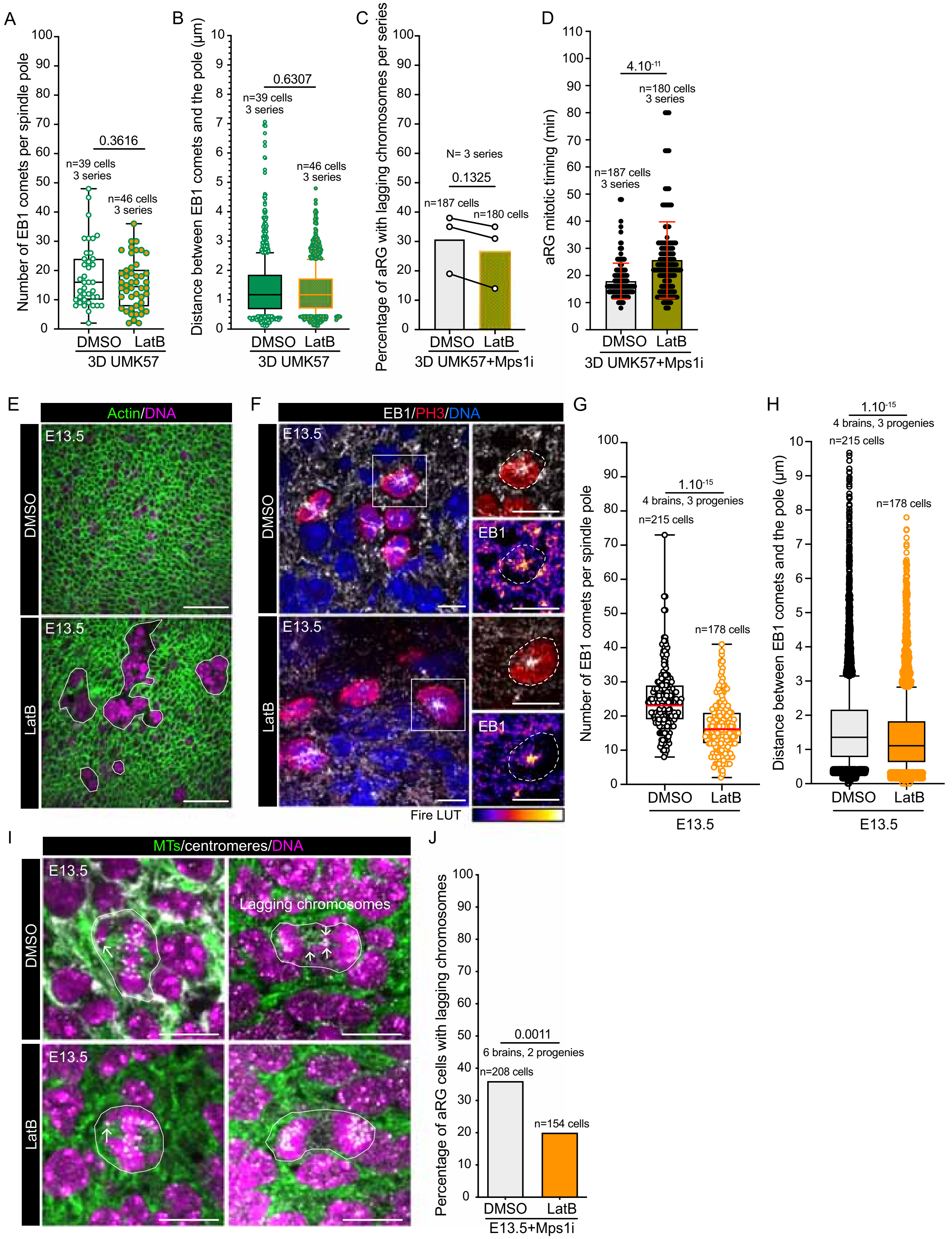
A
Number of EB1 comets per spindle pole
0.3616
n=39 cells
3 series
n=46 cells
3 series
DMSO LatB
3D UMK57
B
Distance between EB1 comets and the pole (µm)
0.6307
n=39 cells
3 series
n=46 cells
3 series
DMSO LatB
3D UMK57
C
Percentage of aRG with lagging chromosomes per series
N= 3 series
0.1325
n=187 cells
n=180 cells
DMSO LatB
3D UMK57+Mps1i
D
aRG mitotic timing (min)
4.10-11
n=180 cells
3 series
n=187 cells
3 series
DMSO LatB
3D UMK57+Mps1i
E
Actin/DNA
E13.5
DMSO
LatB
F
EB1/PH3/DNA
E13.5
EB1
DMSO
LatB
Fire LUT
G
Number of EB1 comets per spindle pole
1.10-15
4 brains, 3 progenies
n=215 cells
n=178 cells
DMSO LatB
E13.5
H
Distance between EB1 comets and the pole (µm)
1.10-15
4 brains, 3 progenies
n=215 cells
n=178 cells
DMSO LatB
E13.5
I
MTs/centromeres/DNA
E13.5
Lagging chromosomes
DMSO
LatB
J
Percentage of aRG cells with lagging chromosomes
0.0011
6 brains, 2 progenies
n=208 cells
n=154 cells
DMSO LatB
E13.5+Mps1i

 © The Author(s)

Figure EV7. Analysis of the impact of biomechanical constraint release by actin depolymerization in E13.5 cerebral cortical explants.

(**A**, **B**) Box and whiskers plots depicting the number (**A**) and the distance traveled (**B**) by EB1 comets in the indicated conditions. (**C**, **D**) Bar graph depicting the mean percentage of cells with lagging chromosomes, with data plotted per series and corresponding mitotic timing. (**E**) Pictures of E13.5 cerebral cortices showing actin (green) and DNA (magenta) at the level of the ventricular lining; white dashed lines highlight disruption of the neuroepithelium. (**F**) On the left, *en face* views of E13.5 cerebral cortices labeled with EB1 and PH3 antibodies shown in gray and magenta. DNA is in blue. On the right, insets show mitotic cells and EB1 comets depicted with the Fire LUT based on FI. (**G**, **H**) Box and whiskers plots showing EB1 comet number (**G**) and distances from spindle poles (**H**). (**I**) Pictures of E13.5 cerebral cortices labeled with antibodies recognizing MTs (green), centromeres (gray), and PH3 (purple). (**J**) Bar graph showing the percentage of cells with lagging chromosomes from the cumulative analysis of 6 brains out of 2 progenies. Data are presented as mean ± SD in (**D**). Median values are indicated by the middle line, and whiskers range from minimum to maximum values (**A**, **G**) or 10–90 percentiles (**B**, **H**). Data were submitted to normality and lognormality tests for the choice of an appropriate statistical test. Statistical significance was assessed with Mann–Whitney test (**A**, **B**, **D**, **G**, **H**), paired *t* test (**C**), and Chi-square test (**J**). Scale bars: (**E**) 20 µm; (**F**) left 5 µm and right 10 µm; (**I**) 10 µm. Source data are available online for this figure.

© The Author(s)
