# Supplementary material for: Mechanical constraints regulate embryonic stem cell mitosis in the developing brain
Source: EMBO J. 2026 Jul 10;45(16):5622–56. doi: 10.1038/s44318-026-00848-3 (PMC13482099; doi:10.1038/s44318-026-00848-3)
Supplement: Supplementary file 2 [file 44318_2026_848_MOESM1_ESM.pdf]

THE EMBO JOURNAL

Mechanical forces regulate embryonic stem cell division in the developing brain

Veronique Marthiens, Lin Jawish, Margaux Malosse, Clara Basto, Denis Krndija, Anne-Sophie Macé, Elmar Laigna, Antonia Terrizzano, Corentin Peyret, Teng Pan, Olivier Zajac, Louisiane Perrin, Elsa Logarinho, Catherine Villard, and Renata Basto

Corresponding author(s): Veronique Marthiens (veronique.marthiens@curie.fr) , Renata Basto (renata.basto@curie.fr)

Review Timeline:

Submission Date:	16th Oct 25
Editorial Decision:	16th Dec 25
Revision Received:	7th May 26
Editorial Decision:	9th Jun 26
Revision Received:	19th Jun 26
Accepted:	23rd Jun 26

Editor: Hartmut Vodermaier

Transaction Report:

(Note: With the exception of the correction of typographical or spelling errors that could be a source of ambiguity, letters and reports are not edited. Depending on transfer agreements, referee reports obtained elsewhere may or may not be included in this compilation. Referee reports are anonymous unless the Referee chooses to sign their reports.)

Dear Renata,

Thank you for submitting your manuscript on mitotic fidelity in the embryonic brain for our consideration. It has now been seen by three expert referees, whose reports are copied below. As you will see, all referees express considerable interest in the work in principle, but also raise a number of substantial concerns, including the over reliance on broad-scale pharmacological manipulations, limited in vivo validation, and uncertain physiological logic and conceptual clarity.

Since it is not clear whether the various major criticisms could be fully addressed to the referees' satisfaction during a regular round of revision, I would at this point invite you to assemble a tentative point-by-point response proposing how you might address particular concerns, and send this back to me by early next week. Based on such a revision plan, which I may in parts share/discuss with some of the referees, and possible follow-up discussion via video call, we could then get a better understanding of whether a revision for The EMBO Journal would seem realistic, or whether a less substantively revised, toned-down version would be a better candidate for rapid publication in one of our sister journals (such as EMBO Reports or Life Science Alliance).

Looking forward to hearing from you,

Best regards,

Hartmut

Hartmut Vodermaier, PhD
Senior Editor, The EMBO Journal
h.vodermaier@embojournal.org

Referee #1 (Report for Author)

Marthiens et al. present an ambitious and technically sophisticated study linking the mechanical environment of the developing brain to mitotic fidelity in apical radial glial (aRG) cells. The authors propose that high tissue density during early neurogenesis increases cortical actin tension, thereby enhancing microtubule polymerization at spindle poles and promoting chromosome mis-segregation. Using a broad range of approaches, in

vivo laser ablation, ex vivo brain explants, custom-designed PDMS confinement assays, and pharmacological perturbations, they offer an integrative view of how biomechanical forces may influence spindle assembly and chromosome segregation. The study is timely, methodologically innovative, and conceptually important because it addresses the underexplored link between tissue-scale mechanics and cell-intrinsic control of genome integrity. The data convincingly show correlations between cortical actin enrichment, increased cortical stiffness, and mitotic error frequency, making a strong case that mechanical context is a determinant of mitotic fidelity in neural progenitors.
However, while the study is elegant and exciting, several issues limit its interpretive strength and conceptual depth. The causal connection between mechanical compression, actin cortex remodeling, MCAK regulation, and chromosome mis-segregation remains largely inferential. The pharmacological manipulations (latrunculin B, CK666, dextran) alter multiple cellular processes and thus cannot unambiguously assign causality to actin-mediated tension. Mechanical measurements rely mainly on relative recoil velocities after laser ablation, which do not quantify actual stress magnitudes; complementary methods such as AFM, would substantiate the mechanical framework. In addition, developmental comparisons between E13.5 and E16.5 might reflect biochemical and metabolic differences beyond mechanics, and the authors should demonstrate that changes in mitotic fidelity are not secondary to other developmental factors. The in vivo validation is also limited: drug treatments of explants are short term and lack controls for potential cytotoxic or off-target effects. Finally, the conceptual model in Fig. 6G simplifies a bidirectional system: microtubule forces themselves can influence cortical actin organization, raising uncertainty about causal directionality.
Overall, the manuscript represents a substantial contribution but requires further mechanistic clarification and stronger quantitative grounding before publication in The EMBO Journal. The issues below should be addressed before the study can be considered definitive.
Major points
1. Demonstrate more directly that cortical actin tension modulates MCAK activity or microtubule polymerization. This could be achieved by monitoring MCAK localization or phosphorylation under compression or actin perturbation, or by testing whether MCAK overexpression rescues mis-segregation phenotypes.
2. Provide absolute measurements of tissue or cell mechanical stress (e.g., AFM indentation, microindentation, or optical tweezers) to support claims about compression and cortical tension.
3. Extend analysis beyond short-term explant assays, ideally through mechanical relaxation or local actin modulation in intact embryos, to establish physiological relevance.
4. Developmental specificity. Control for biochemical differences between E13.5 and E16.5

stages (cell cycle duration, polarity markers) to confirm that observed effects truly derive from mechanics.
5. Statistical transparency. Include full reporting of biological replicates, sample numbers, and variance for all quantitative data.
6. Physiological significance. Discuss to what extent the measured two-fold increase in lagging chromosomes can impact neurogenesis and brain size, integrating these effects into broader developmental or disease contexts such as microcephaly.

Minor points

1. Clarify terminology, phrases such as "cortical tension acts as a sensor" are misleading; tension is a consequence, not a sensor.
2. Improve figure legends with clearer statistical annotations and scale bars; some panels lack units or replicate information.
3. Simplify and reorganize the discussion to separate descriptive results from speculative interpretation; the current flow is somewhat repetitive.
4. Indicate whether drug treatments (LatB, CK666) affected cell viability or mitotic progression independently of mechanical changes.
5. Provide higher-resolution images for EB1 comet analyses and ensure that quantification criteria (thresholds, region selection) are transparent and reproducible.
6. Adjust color schemes throughout figures to colorblind-friendly palettes (e.g., magenta-cyan or orange-blue instead of red-green) to ensure accessibility and improve contrast for printed and digital formats.

Referee #2 (Report for Author)

In their article, the authors present the idea that biomechanical stress on spindles in the developing neocortex can add to mitotic errors. According to the authors, this is particularly true at early developmental stages when crowding is most severe. While experiments are done in a controlled and quantitative manner, some of the logic of the outcomes is not yet fully clear and would profit from some additional explanations.

General Points:

Introduction:

The introduction is very concise at places and rationales and arguments are not always fully worked out. For example, it could be better explained why the cortex would be more sensitive to chromosome segregation errors and more failed mitosises than other highly dividing tissues, including other epithelia, in the developing organism.

Line 60-61, the types of tissue remodelling referred to should be at least briefly mentioned

as should the fact that neurogenesis proceeds even after birth in many vertebrates including mammals.
Line 66-67, the rationale for studying 'biomechanics' in mitosis could be better worked out.

General logic:

- If detrimental for development at times, it is not clear why such biomechanical sensitivity persists over evolution in normal brain development. Conceptually, one would question why such sensitivity would persist. The authors could expand on that logical argument.
- Along the experimental outlines, it is slightly counterintuitive how interference with the actin cortex and thereby cortex-spindle interactions lead to less chromosome segregation errors. This could be further discussed (see also below).

Experimental Points:

- Ideally, crowding of the tissue should also be shown along the apicobasal axis to underline the statement that mitotic crowding is highest at early stages in the mitotic zone. While 3D analysis is probably too tedious, some 2D analysis using Cellpose or similar tools from apical to basal could be performed.
- Fig. 1F, some raw data on the analysis of spindle morphology should be added to the supplement.
- In Figure 2 the analysis pipeline of lagging chromosomes should be better explained and documented. The presented images look very blurry and it is unclear how scoring can be made reliable and reproducible over different culture conditions. Overall, some comment on the fates of these lagging chromosomes should be made as often these can still be caught and integrated into the spindle without causing aneuploidy. Maybe some estimation of the number of aneuploid cells in different conditions could be given.
- The link between the increase of astral microtubules and chromosome segregation errors could be better explained.
- Some, even qualitative, in vivo data on the rate of MT polymerization at the spindle poles would help the authors' arguments in addition to the ex vivo data presented.
- When it comes to the upregulation of cortical actin upon compression, what do the authors mean by 'sensor'? Would it not be possible that this is a tissue/cellular reaction to stiffening of the cortex in a stiffer environment that feeds back on all cells?
- What do the authors speculate is the nature of the spindle-astral microtubules-cortex interplay and how is this related to the tissue crowding?

Minor Points:

- Nowadays red/green annotated figures are no longer widely used in the field to make figure interpretation easier for people with compromised colour vision.
- The graphs, while correct, are on first sight not very intuitive. It might be more intuitive to plot all data points (not just outliers) or use methods like violin plots. It is at the authors' discretions though.
- Figure S2 F, it seems that in this experimental set-up lagging chromosomes are slightly more abundant in the 2D than in the 3D condition in the untreated scenario. Do the authors have an explanation for this?
- Line 102, it is not clear what is meant by 'differences in mitotic fidelity'.
- As the authors do not really measure 'mechanical forces at play' except in the laser ablation experiments, the language of what is measured and what a possible interpretation should be adapted.

Referee #3 (Report for Author)

General summary and opinion about the principle significance of the study, questions and findings

In their manuscript, Marthiens and colleagues adress the effects of tissue biomechanics on mitotic fidelity, using the developing mouse brain as model system. This is a relevant question that has been underexplored in tissue biology in general, and in developmental neurobiology in particular. The authors deploy a combination of in vivo, ex vivo 2D and 3D systems combined with pharmacological perturbation to address the question and precisely dissect the contribution of different players such as forces, cell-cell adhesion, tissue crowdiness and cytoskeletal components.

Specific major concerns essential to be addressed to support the conclusions

The authors leverage and heavily rely on pharmacology to dissect the involvment of several pathways and molecular plalyers. Althugh the choice is understandable from an experimental point of view, considering the complexity of the developing brain tissue and the possibility of drug's side effects and/or secondary effects involving cell types other than aRGs, it is necessary to corroborate the main findings using also a genetic manipulation (via elecroporation of specific shRNAs, e.g.).
I am referring in particular to the data presented in the paragraphs "Cortical actin senses biomechanical compression in mitotic aRG cells", that are of great relevance for the precise identification of actin as the main molecular pathway involved via the manipulation of Arp2/3. The same applies to the demonstration of actin working upstream of MCAK (in "Cortical actin acts upstream of MCAK to tune polar MT polymerization and modulate chromosome segregation errors"). Again, it would be important to corroborate the data obtained via pharmacological manipulation with a genetic manipulation as well.
The genetic manipulation will make the findings more convincing and related conclusions stronger.

The general idea of tissue mechanics affecting mitotic fidelity is intriguing. The authors should eleborate more on this point in the discussion. In its present form, the authors mentioned/speculate a possible involvement of tissue mechanics in neurodevelopmental disease. This is a very general statement, and t is not really clear what the authors exactly means with that. In addition, from a logical point of view: why the most relevant aspect should be disease, rather than physiology? I urge the authors to develop a deeper and more thought-provoking discussion on the possible implications of biomechanics and mitotic fidelity in physiology.

In that respect, and following the line of thoughts suggested by their data, the authors should consider/discuss the possibiity that basal mitosis (that are typically happening in a less crowded niche compared to the ventricular zone), could show a higher mitotic fidelity compared to apical mitosis. This is a very interesting point to consider, as one of the main argument for the evolutionary switch to basal mitosis has always been the maximization of the total number of mitosis per ventricular surface lenghth. The data shown by the authors make me wonder if the switch to a higher proportion of basal mitosis could be linked to the need of a higher mitotic fidelity as well. I am aware that this might not be the main interest of the Basto lab, but if the authors were to have quantifications on the occurrence of lagging chromosomes in apical vs basal mitosis, that could be mentioned and discussed. I believe this will increase tremendously the interest of the manuscrip for the brain evo-devo

community.

Minor concerns that should be addressed

The authors use the terms early neurogenesis and late neurogenesis when referring to E13 and E16, respectively. However, in the field of developmental neuroscience early neurogenesis is typically referred to E10-E11. I advice the authors to change the pharasing, to make the classification more sound and less misleading to neurogenesis experts.

Paragraph 174-186 "Since MT polymerization activity is asymmetric between the two spindle poles, we plotted the data of the pole displaying the highest activity."
Is the MT polymerization activity regulated proportionally at the two spindle poles?
In other words, it would be interesting (using the data the authors gathered already) to understand if constraints are making the spindle more asymmetric. This is a basic tissue cell biology question the authors have the tools to adress. I invite them to do so.

Reponses to reviewers of EMBOJ-2025-122764, Marthiens et al

Reviewer 1:

The causal connection between mechanical compression, actin cortex remodeling, MCAK regulation, and chromosome mis-segregation remains largely inferential.

We do agree and disagree with this statement. Our work provides compelling evidence of the connection between mechanical compression, actin cortex remodeling and chromosome mi-segregation. However, the reviewer is right when considering the implication of MCAK, which in this study has relied solely on the use of the agonist UMK57.

To overcome this point, we propose to include new data where MCAK depletion and over-expressing conditions will be used.

1. Demonstrate more directly that cortical actin tension modulates MCAK activity or microtubule polymerization. This could be achieved by monitoring MCAK localization or phosphorylation under compression or actin perturbation, or by testing whether MCAK overexpression rescues mis-segregation phenotypes.

We will certainly provide the characterization of MCAK localization and activity in our different settings, as suggested. The referee suggests as well to overexpress MCAK and monitor the rescue of chromosome missegregation phenotypes. We have already provided data where global MCAK activity is increased in 3D aRG cultures after incubation with the UMK57 agonist (Figure 3I-M) showing a decrease in microtubule depolymerization from the spindle pole along with decrease in chromosome segregation errors, as reported in the literature in other cell types (Barroso-Vilares & Logarinho, 2019; Orr et al., 2016). More, we have shown that 3D aRG are not sensitive to actin depolymerization anymore -no changes in MT polymerization from the spindle poles (Fig5M-N) nor in the frequency of lagging chromosomes (Fig5O) after latrunculin B treatment- when MCAK activity is increased, in favor of our model of actin acting upstream of MCAK activity.

We now obtain exactly the same results when cortical actin is reorganized after delocalization of the ARP2/3 complex from the plasma membrane (CK666 drug treatment) in combination with increased MCAK activity (UMK57 drug treatment). We propose to include this new set of data in the manuscript to reinforce the causal relationship between cortical actin and MCAK activity acting upstream of microtubule polymerization from the spindle pole and chromosome missegregation. Still, as this reviewer requests, we will perform experiments of MCAK over-expression and loss-of-function.

2. The pharmacological manipulations (latrunculin B, CK666, dextran) alter multiple cellular processes and thus cannot unambiguously assign causality to actin-mediated tension.

This reviewer is right, of course. Any drug treatment will alter several cellular processes on a long-term basis. The same effect is expected for any genetic manipulation of members of the actin cytoskeleton. We choose to use this approach after careful characterization of the drug effects. Please bear in mind that our drug

treatments were quite short to avoid the accumulation of any cellular stress or phenotypes that could indirectly contribute to a specific behavior. Further, before each set of experiments, we performed a dose-response analysis to find the lowest dose to be used to decrease in a quantitatively detectable manner actin recruitment at the plasma membrane in 3D aRG cultures (Figure S5D for latrunculin B treatment, Figure 5H-I for CK666 treatment) or detect cell shape changes after dextran incubation (Figure 4A-B). Moreover, as mentioned above, all the functional assays were performed after a short exposure to the drug or incubation with dextran, i.e., 1 hour for microtubule depolymerization-repolymerization assays and 2.5 hours for time-lapse recording. Such short-time windows aimed at restricting our analysis to cells in preparation for mitosis. The G2 phase length has been previously evaluated to be around 2 hours in aRG cells at this developmental stage (Arai et al., 2011) and so this short period of time ensures a minimal potential side effect. It is well known that nuclear actin plays an essential role in DNA damage response (Caridi et al., 2018; Palumbieri et al., 2023), and we do not want to interfere with this process, which can ultimately lead to indirect mitotic errors.

Additionally, even if compressive forces were mimicked with different approaches (PDMS devices in Figure 3A-E or incubation with dextran in Figure 4), the same increase in microtubule polymerization activity from the spindle poles was noticed in 2D aRG cultures. Finally, while latrunculin B and CK666 treatments perturb both microtubule polymerization capacity from the spindle poles and mitotic fidelity in 3D aRG cultures, we did not observe any effect in aRG cultures in 2D (FigS6B-E for latrunculin B or Figure S6G-H and not shown for CK666). The lack of effect in 2D attests for a lack of perturbations that could contribute to these phenotypes. Altogether, these observations strongly argue in favor of compressive forces conveyed by ARP2/3-dependent branched cortical actin at the origin of changes in spindle pole microtubule polymerization activity and chromosome segregation. Again, we do not think that a genetic perturbation is ideal here. We require an acute, fast way of decreasing actin polymerization levels. Take into consideration that cells lacking actin or with low actin levels will undergo cell shape changes and more importantly, will fail cytokinesis (Lancaster & Baum, 2014; Taubenberger et al., 2020), we would be in a position where any type of conclusion would not be possible to ascertain to a role of cortical actin. In other words, we would be studying a cumulative effect of actin depolymerization.

We propose to explain this rationale when introducing the actin depolymerization experiments. It is also worth mentioning that we did try to analyze a conditional ARPC4 knockout mouse model (Arpc4<tm1a(EUCOMM)Wtsi> Targeted Allele Detail MGI Mouse (MGI:4433308), but similarly to what has been published in other tissues (Perrin et al., 2025) and in the mouse brain (Wang et al., 2016), this lead to loss of epithelial tissue integrity. The brains from these mice were extremely soft, disrupted and difficult to handle. For these reasons, we decided to use the alternative pharmacological approaches.

3. Mechanical measurements rely mainly on relative recoil velocities after laser ablation, which do not quantify actual stress magnitudes; complementary methods such as AFM, would substantiate the mechanical framework.

Provide absolute measurements of tissue or cell mechanical stress (e.g., AFM indentation, microindentation, or optical tweezers) to support claims about

compression and cortical tension.

It is true that our approaches provide relative measures. But this is the case of many studies performed in tissues such as (Krndija et al., 2019; López-Gay et al., 2020; Rujano et al., 2013). They certainly do not replace absolute measures, but they provide with a basis to compare cortical tension within different conditions. We chose this strategy because in the mouse developing brain, the use of atomic force microscopy (AFM) in different regions at different developing stages of the mouse brain showed that tissue stiffness could not be explained by the stiffness of the cells that constituted the tissue (Iwashita et al., 2014). We are convinced that the measurements of stiffness performed by AFM cannot reflect an absolute measurement of cortical tension of individual cells, but instead reflects the contribution of multiple parameters, including the type of microtubule cytoskeleton and properties, extracellular matrix composition, which are outside of the scope of our study. Even if not an absolute measure, relative differences in recoil velocity after laser ablation is to our knowledge the only quantitative approach used at the scale of a tissue that reflect an evaluation of actin-dependent cortical tension of individual cells (Krndija et al., 2019; López-Gay et al., 2020). These experiments, shown in our study were very difficult to perform and were controlled to use different samples at the same day with the same laser conditions. We propose to explain this better and to recognize possible limitations of relative measures.

In our lab, we have made huge efforts to use optical tweezers to probe cell mechanical-properties. After weeks and weeks of attempts, we decided that these were not compatible with our models.

We understand that the referee wishes to be more convinced regarding the link between compression and actin-based cortical tension *in vivo*. We have a clear correlation between increase in cell density (Figure 1B-C), actin recruitment at the plasma membrane (Figure 5A-C) and cortical membrane tension after velocity recoil measurement (Figure 5D-F) when comparing E13.5 and E16.5 aRG in mitosis. Individual mitotic cell size (Figure 1D), shape (Figure 1E-G) and volume (Figure 1H) are also directly related to differences in cell density in the ventricular zone. As our *ex vivo* dextran experiment (Figures 4 and S4) supports the view that imposing mechanical compressive forces of 10kPa to individual mitotic cells is sufficient to mimic the *in vivo* changes in terms of microtubule polymerization activity and mitotic error frequency, we favored the interpretation that compressive forces imposed by cell density is the force at play *in vivo*. We do not think that it will be experimentally possible to link cell density and compressive forces *in vivo.* We will discuss based on the dextran experiments, the possibility that compression might be one of the stresses sensed upon cell density change *in vivo*.

To more directly ascertain this reviewer's point, we propose to provide lateral views of embryonic brain tissues at E13.5 and E16.5 and to probe in parallel mitotic cell surroundings (density) and ARP2/3 levels at the plasma membrane (similar to the staining of ARPC5 subunit provided in Figure 6A and 5H-I), which is a well-established readout of cortical tension (Chugh et al., 2017; Rosendahl et al., 2018; Toyoda et al., 2017).

4. In addition, developmental comparisons between E13.5 and E16.5 might reflect biochemical and metabolic differences beyond mechanics, and the authors should demonstrate that changes in mitotic fidelity are not secondary to other developmental factors.

Developmental specificity. Control for biochemical differences between E13.5 and E16.5 stages (cell cycle duration, polarity markers) to confirm that observed effects truly derive from mechanics.

Our choice of performing *ex vivo* biomechanical perturbation assays using the same populations of aRG cells at the same stage of development was driven by the intention to discard developmental stage-related neural lineage fate changes from our interpretation. Cell cycle lengthening has been previously reported during neurogenesis along with neural cell fate commitment (Arai et al., 2011; Calegari et al., 2005). We therefore expect changes in cell cycle length, and very likely polarity markers as well between E13.5 and E16.5. Nonetheless, based on the data provided in the manuscript, we are confident with the fact that spindle pole microtubule polymerization and mitotic fidelity can be tuned by cell density-related stress in the embryonic brain. It is not our intention to say that it is the only factor that contribute to mitotic fidelity, but instead one factor to be considered, which has never been reported before. Indeed, we have already shown differences in the loading of the microtubule-associated protein TPX2 on spindle microtubules between E13.5 and E16.5 mitosis responsible for changes in spindle morphology and related mitotic fidelity (Vargas-Hurtado et al., 2019). Our work shows that biomechanics is one parameter that has to be considered in the regulation of mitotic fidelity in the developing brain. We will state this very clearly in the text and we would be happy to change the title so that it is not interpreted as the only parameter.

Our results show that E13.5 aRG cells are mechanosensitive in mitosis and that changes in the density of surrounding cells are sufficient to alter microtubule polymerization characteristics and mitotic fidelity. It is also important to mention that aRG cells in culture as floating spheroids (Figure S2A-B) lack polarity (data not shown). As release of mechanical tension by actin cytoskeleton perturbation have the same impact on microtubule polymerization and mitotic fidelity in 3D aRG cultures (absence of polarity) and cortical explants (polarity preserved), it is likely that polarity is not a main contributor.

We have already experimental evidence that E16.5 aRG cells are also mechanosensitive in mitosis. Indeed, we observed an increase of the population of astral microtubules in mitosis when they are confined in the PDMS grooves, similar to what we have reported in Figure 3A-E for E13.5 aRG cells. These observations indicate that experimental conditions that mimic cell density (passive confinement imposed by the PDMS grooves) can also increase astral microtubule population at E16.5. This fuels the concept that mechanical parameters contribute at both stages. We will include this data in the revised version of the manuscript.

In parallel, we propose to perform dextran experiments to reimpose the same level of mechanical stress and compare in a more quantitative manner E13.5 to E16.5 aRG cells regarding microtubule polymerization rate and mitotic fidelity in these conditions. This will reinforce the fact that aRG, whatever neural lineage commitment characteristics, are able to respond to the same mechanical cues.

5. The in vivo validation is also limited: drug treatments of explants are short term and lack controls for potential cytotoxic or off-target effects.

Short treatments of cortical explants were indeed performed with the intention to avoid long-term cytotoxic effects of drug treatments. For example, we have already mentioned that cytokinesis failure and the generation of polyploid daughter cells (that did not undergo final cell division) is one expected side effect of genetically altered

actin levels or high level of actin depolymerizing drugs. This is the reason behind short-term actin drug treatments (less than 3 hours). We can explain this point better and even provide images showing the altered tissue organization of the ventricular zone in conditional ARP2/3 loss-of-function mutant mouse line. Indeed, we attempted to perform these experiments with this mouse line, but it was not possible, as the tissue was very abnormal, but we just found the same levels of abnormal tissue integrity as previously published by (Wang et al., 2016).

Extend analysis beyond short-term explant assays, ideally through mechanical relaxation or local actin modulation in intact embryos, to establish physiological relevance.

Demonstrating the modulatory effect of mechanobiology in an intact tissue is a real challenge in a mammalian organism. Indeed, the actin cytoskeleton is involved in many processes, including the capacity of cells to interact in tissues. Perturbing its integrity on a long-term scale may lead to an extreme disorganization of the tissue, i.e., global or local dysplasia, as already observed after adhesion molecule loss-of-function (N-cadherin KO mouse model to cite an example (Kadowaki et al., 2007)). We can also think of compensatory mechanisms that might be misleading in the interpretation of the results and polyploidization of the tissue after cytokinesis failure, which is an expected side effect of ARP2/3 loss-of-function.

In fact, we agree with the referee that genetic approaches using the ARPC4 KO mouse model for example (which will have a similar effect than CK666 drug treatment) would be a good approach, complementary to our pharmacological tools to get a long-term impact of biomechanical perturbation at the scale of the tissue. If this approach has been used to unravel the impact of cortical actin-based tension in mouse oocytes for example (Nikalayevich et al., 2024), one has to keep in mind that multicellularity makes this approach really challenging, as explained above. In fact, we have already obtained preliminary results by analyzing a partial ARP2/3 loss-of-function brain (ARPC4 +/-; Nestin-Cre+/-) and observed a confounding disruption of the ventricular lining, so expected dysplasia. These preliminary observations have precluded us from using long-term genetic approaches. Instead, we have decided to use our strategy of acute drug perturbation assays on intact telencephalic vesicles for short-term scale analysis.

6. Statistical transparency. Include full reporting of biological replicates, sample numbers, and variance for all quantitative data.
The full data sheet with all the information will be provided in the revised version of the manuscript.

7. Physiological significance. Discuss to what extent the measured two-fold increase in lagging chromosomes can impact neurogenesis and brain size, integrating these effects into broader developmental or disease contexts such as microcephaly.

Our view is that the high cell density, which results from high proliferation rate in the ventricular zone at early stages of neurogenesis, is an Achill heal for mitotic fidelity. Such mitotic vulnerability does not have obvious consequences on tissue homeostasis during development, when the frequency of abnormal mitotic figures is very low. Abnormal genome containing cells are efficiently removed from the cycling population (by apoptosis or premature neuronal differentiation according to the literature and reviewed in (Marthiens & Basto, 2020). We have previously evaluated less than 2% of

caspase-3 positive apoptotic cells in a normal mouse brain during neurogenesis (Marthiens et al., 2013).

Nonetheless, this vulnerability has obvious consequences on brain size in pathological conditions when mitosis is challenged, like in microcephaly-related disorders indeed. When abnormal mitotic figures with lagging chromosomes in anaphase are scored, there is one chance out of two that the chromosome that lags behind integrates the wrong nucleus and generates two aneuploid daughter cells that will be discarded from the proliferative pool. We therefore consider that two times more lagging chromosomes in anaphase in a population at a given time point corresponds to a doubling in the number of cells to be eliminated. Quantitative correlations between lagging chromosomes in anaphase, number of daughter cells eliminated after mitosis and brain size reduction at birth have not been directly assessed by lineage tracing of individual cells but reported at the global tissue level. Moreover, brain size reduction varies depending on the frequency and nature of abnormalities, on the developmental stage considered….

We will discuss these points more extensively.

Minor points

1. Clarify terminology, phrases such as "cortical tension acts as a sensor" are misleading; tension is a consequence, not a sensor. We will correct this terminology.
2. Improve figure legends with clearer statistical annotations and scale bars; some panels lack units or replicate information. The figures and figure legends will be changed accordingly.
3. Simplify and reorganize the discussion to separate descriptive results from speculative interpretation; the current flow is somewhat repetitive. We will make these changes in the organization of the discussion.
4. Indicate whether drug treatments (LatB, CK666) affected cell viability or mitotic progression independently of mechanical changes. We used very low doses of any of these drugs, and did not notice any change in cell viability above control condition or in mitotic timing (from metaphase to anaphase). We can provide these data after Latrunculin B or CK666 treatments alone. It is to note that the concentration of drug to be added in the medium was chosen according to the absence of such side effects. It is also important to remind that all our experiments were performed in a short time window (less than 3 hours).
5. Provide higher-resolution images for EB1 comet analyses and ensure that quantification criteria (thresholds, region selection) are transparent and reproducible. We will provide higher resolution images for EB1 comet analysis. All the quantification procedure and criteria will be further detailed in the supplementary figure 3. The Fiji plugin for analysis of EB1 comets included manual correction options. For example, it included the possibility to draw the pole of interest, the region of interest around each pole, to add non detected comets, remove wrongly detected ones…we will provide the different steps of the procedure for better evidence of transparency.
6. 6. Adjust color schemes throughout figures to colorblind-friendly palettes (e.g., magenta-cyan or orange-blue instead of red-green) to ensure accessibility and improve contrast for printed and digital formats.

We will make these color changes for all the figures.

Reviewer 2:

Introduction:

The introduction is very concise at places and rationales and arguments are not always fully worked out. For example, it could be better explained why the cortex would be more sensitive to chromosome segregation errors and more failed mitoses than other highly dividing tissues, including other epithelia, in the developing organism. Line 60-61, the types of tissue remodeling referred to should be at least briefly mentioned as should the fact that neurogenesis proceeds even after birth in many vertebrates including mammals.
Line 66-67, the rationale for studying 'biomechanics' in mitosis could be better worked out.

We will explain better the rationale behind our study.

Neurogenesis proceeds only before birth in the cerebral cortex in mammals. Because of time restriction and challenge to produce enough neurons to populate the different cortical layers, the rate of proliferation and therefore cell density in the ventricular zone is higher than in other regions of the brain and epithelia of the organism. In genetic forms of the human MCPH pathology, cerebral cortex size reduction at birth results from depletion of the embryonic neural progenitor pool consequent to defects in mitotic progression. Nonetheless, with the same mutation, the other tissues of the organism are spared in their development. We therefore aimed to identify the reasons behind the vulnerability of neuronal brain progenitors. As genetic mouse models for microcephaly report a prevalence of mitotic defects in the aRG population of the ventricular zone (Marthiens et al., 2013; Shi et al., 2019), characterized by high cell density, we wanted to evaluate a potential causal relationship between cell density and mitotic defects in this study.

General logic:

- If detrimental for development at times, it is not clear why such biomechanical sensitivity persists over evolution in normal brain development. Conceptually, one would question why such sensitivity would persist. The authors could expand on that logical argument.

This is a very good point and we will add it in the discussion. Our view is that this mechano-sensitivity is primarily required to adjust the rate of MT polymerization from the spindle poles and maintain the population of astral MTs for proper positioning of aRG cells in the neuroepithelium. Failure to do so will result in dysplasia of the tissue and disorganized cortical layering at birth. Nonetheless, a drawback of this mechano-sensitivity and high rate of MT polymerization is an increased probability to generate mitotic errors. As safeguard mechanisms exist to detect and correct these errors (like the spindle assembly checkpoint that we challenge with our Mps1 inhibitor), and to eliminate unfit cells from the cycling population (by apoptosis or premature neuronal differentiation), the consequences on brain size is undetectable. It is only in pathological conditions that this vulnerability is unraveled with consequences on brain size and functionality.

Along the experimental outlines, it is slightly counterintuitive how interference with the actin cortex and thereby cortex-spindle interactions lead to less chromosome segregation errors. This could be further discussed (see also below).

This is also a very interesting discussion point. It seems indeed that the majority of cortex-spindle interactions enabled by increased MT polymerization from the spindle

pole comes at the expense of the efficiency of chromosome capture and/or error correction mechanisms. The MCAK microtubule-depolymerase activity playing a dual role in modulating MT polymerization from the spindle pole and stabilizing correct chromosome attachment could mechanistically explain this interdependence. This possibility is highly fueled from our fundamental knowledge of MCAK mechanistic role in the literature.

Experimental points:

- Ideally, crowding of the tissue should also be shown along the apicobasal axis to underline the statement that mitotic crowding is highest at early stages in the mitotic zone. While 3D analysis is probably too tedious, some 2D analysis using Cellpose or similar tools from apical to basal could be performed.

We will compare lateral views of the tissue at E13.5 and E16.5 and display quantitative measurements of cell density after segmentation.

- Fig. 1F, some raw data on the analysis of spindle morphology should be added to the supplement.

Figure 1F displays comparison of cell volume after segmentation at E13.5 and E16.5. As we have already thoroughly analyzed changes in spindle morphology at these two stages of development and provided quantitative analysis (Vargas-Hurtado et al., 2019), we understand that the referee wishes that we include images of spindle morphology related to cell volume. We will include these images.

- In Figure 2, the analysis pipeline of lagging chromosomes should be better explained and documented. The presented images look very blurry and it is unclear how scoring can be made reliable and reproducible over different culture conditions.

We will provide better quality images and 3D reconstructions in the main figure 2, as well as corresponding movies used for analysis as supplementary material in order to better explain our scoring procedure for lagging chromosomes.

Overall, some comment on the fates of these lagging chromosomes should be made as often these can still be caught and integrated into the spindle without causing aneuploidy. Maybe some estimation of the number of aneuploid cells in different conditions could be given.

The referee is right. There is one chance out of two that the merotelic attachment, results in an error in chromosome segregation, i.e., generates two aneuploid cells. When scoring higher frequency of lagging chromosomes, we therefore score higher probability of mitosis to generate aneuploid progeny. These correlation between merotelic attachments, lagging chromosomes and aneuploidy are well documented in the literature and we will better explain this point in the main text. Estimation of the number of aneuploid cells generated in conditions where actin cytoskeleton is perturbed is difficult as cytokinesis fail at the end of cell division, preventing final cell division between the two daughter cells. Moreover, we have evidence that aneuploid cells are rapidly eliminated from the tissue. Indeed, we have sequenced single cell DNA from progenitor cells treated with mitotic poisons. We did not detect an increase in aneuploidy when compared to controls, most likely because these cells are rapidly eliminated. We can mention these results and discuss the possible frequencies of aneuploidy and their outcomes.

- The link between the increase of astral microtubules and chromosome segregation errors could be better explained.

We will explain this better, it is related with the work published *in vivo*, in the (Vargas-Hurtado, 2019) article. Our work now, provides an upstream link with cortical rigidity and cell density influencing microtubule polymerization rates and consequently mitotic fidelity.

- Some, even qualitative, in vivo data on the rate of MT polymerization at the spindle poles would help the authors' arguments in addition to the ex vivo data presented.

We are not sure of understanding this request. Does the reviewer suggest that EB1 comets should be characterized *in vivo,* in unchallenged conditions (without microtubule depolymerization)?

Microtubule depolymerization-repolymerization assays remain the best functional quantitative evaluation of microtubule polymerization properties, which justify short-term (1 hour maximum) *ex vivo* experiments. We have used EB1 comet number and distance from the spindle poles as an evaluation of MT polymerization parameters. If EB1 comet parameters can be analyzed in the tissue after 1hour long microtubule depolymerization on ice and 3 minutes-long cumulative repolymerization events, the same parameters are difficult to extract at steady-state. EB1/Kif18B and MCAK accumulate in a complex at the plus ends of astral microtubules polymerized from the spindle poles (McHugh & Welburn, 2023; Stout et al., 2011). As Kif18B is only regulating astral microtubule population, we will try to unravel the density of astral microtubules, using Kif18B as a marker.

- When it comes to the upregulation of cortical actin upon compression, what do the authors mean by 'sensor'? Would it not be possible that this is a tissue/cellular reaction to stiffening of the cortex in a stiffer environment that feeds back on all cells?
This point was also brought up by another reviewer. We will explain this better, we meant to state that the increase in branched cortical actin may be due to the response of mitotic cells to increase in cell density in their micro-environment.

- What do the authors speculate is the nature of the spindle-astral microtubules-cortex interplay and how is this related to the tissue crowding?
Factors that are important to regulate the interactions between plasma membrane and astral MTs for spindle positioning (active form of ERM proteins, FigS5A-C or NuMA, data not shown) are enriched at earlier stages of neurogenesis, when cell crowding is higher. Our interpretation is that increase in cell-cell interactions upon cell crowding (as mimicked by Ncadherin-Fc coating on the lateral sides of the PDMS grooves in Fig3A, D-E) favors astral microtubule stabilization at the plasma membrane, but not MT polymerization from the spindle poles *per se* (Figure S3E-F). Instead, accumulation of branched cortical actin at the plasma membrane initiates a yet-to be characterized signal that culminates in microtubule polymerization from the spindle poles. We are currently characterizing the behavior of certain mitotic factors. We can include these in the revised version.

Minor points:

Nowadays red/green annotated figures are no longer widely used in the field to make figure interpretation easier for people with compromised colour vision.
We will make these color changes for all the figures.

- The graphs, while correct, are on first sight not very intuitive. It might be more intuitive to plot all data points (not just outliers) or use methods like violin plots. It is at the authors' discretions though.
The graphs provided are generally helpful to plot both mean and variance. We can plot all data points, as in Figure 1 though.

- Figure S2F, it seems that in this experimental set-up lagging chromosomes are slightly more abundant in the 2D than in the 3D condition in the untreated scenario. Do the authors have an explanation for this?
The referee has righteously noticed the fact that DMSO anhydrous treatment alone increases the frequency of lagging chromosomes in 2D cultures, but not in 3D cultures, as compared to the control situation. However, this is not statistically significant and the differences are quite small – less than 2%. We will make reference to these, but we think they may just reflect experimental variations.

- Line 102, it is not clear what is meant by 'differences in mitotic fidelity'.
We will rephrase this sentence. Our previous work has demonstrated higher frequency of lagging chromosomes at E13.5 as compared to E16.5 after mitotic challenge with MPS1 or CENPE inhibitor of cerebral cortex explants. These differences in mitotic fidelity between the two stages is correlated with the *in vivo* differences observed in mitotic cell density, shape, size and volume in the neuroepithelium.

- As the authors do not really measure 'mechanical forces at play' except in the laser ablation experiments, the language of what is measured and what a possible interpretation should be adapted.

We will also rephrase. Indeed, we have measured relative differences inn cortical tension in relation with cell density.

Reviewer 3:

1) The authors leverage and heavily rely on pharmacology to dissect the involvment of several pathways and molecular players. Although the choice is understandable from an experimental point of view, considering the complexity of the developing brain tissue and the possibility of drug's side effects and/or secondary effects involving cell types other than aRGs, it is necessary to corroborate the main findings using also a genetic manipulation (via elecroporation of specific shRNAs, e.g.). I am referring in particular to the data presented in the paragraphs "Cortical actin senses biomechanical compression in mitotic aRG cells", that are of great relevance for the precise identification of actin as the main molecular pathway involved via the manipulation of Arp2/3. The same applies to the demonstration of actin working upstream of MCAK (in "Cortical actin acts upstream of MCAK to tune polar MT polymerization and modulate chromosome segregation errors"). Again, it would be important to corroborate the data obtained via pharmacological manipulation with a

genetic manipulation as well.
The genetic manipulation will make the findings more convincing and related conclusions stronger.

We understand that this reviewer prefers genetic approaches. So do we, as after all, we are a genetics lab, which has done many studies using fly genetics. But unfortunately, we tried to do *in vivo* genetic approaches without success. We used the ARPC4 conditional mouse model for example, which has been used to study the link between cortical membrane tension and chromosome segregation in mouse oocytes (Nikalayevich et al., 2024). However, when we analyzed the ventricular zones, we observed a disruption of the ventricular lining and tissue dysplasia, a disorganization already reported in the literature after ARP2/3 loss-of-function (Wang et al., 2016). This is exactly why we had to use the acute drug perturbation assays with whole dorsal telencephalon of preserved morphology for short-term scale analysis (Figure 6). We propose to explain this point in the text and to show the images of ARP2/3 brains. Reviewer 1 also raised this point. Please read above.

The general idea of tissue mechanics affecting mitotic fidelity is intriguing. The authors should elaborate more on this point in the discussion. In its present form, the authors mentioned/speculate a possible involvement of tissue mechanics in neurodevelopmental disease. This is a very general statement, and this not really clear what the authors exactly means with that. In addition, from a logical point of view: why the most relevant aspect should be disease, rather than physiology? I urge the authors to develop a deeper and more thought-provoking discussion on the possible implications of biomechanics and mitotic fidelity in physiology.

Our view is that the high cell density, which results from high proliferation rate in the ventricular zone at early stages of neurogenesis represents a vulnerability during mitosis for chromosome segregation. Such mitotic vulnerability does not have obvious consequences on tissue homeostasis during normal development, when all mitotic genes are expressed in a wild type manner. Nonetheless, in conditions where mitotic genes are mutated, even like in MCPH, the consequences are more important than in other tissues. This results in a premature depletion of the progenitor pool resulting in reduction of neuronal layers and therefore brain size at birth. We will further discuss this point in the discussion.

In that respect, and following the line of thoughts suggested by their data, the authors should consider/discuss the possibiity that basal mitosis (that are typically happening in a less crowded niche compared to the ventricular zone), could show a higher mitotic fidelity compared to apical mitosis. This is a very interesting point to consider, as one of the main arguments for the evolutionary switch to basal mitosis has always been the maximization of the total number of mitosis per ventricular surface lengh. The data shown by the authors make me wonder if the switch to a higher proportion of basal mitosis could be linked to the need of a higher mitotic fidelity as well. I am aware that this might not be the main interest of the Basto lab, but if the authors were to have quantifications on the occurrence of lagging chromosomes in apical vs basal mitosis, that could be mentioned and discussed. I believe this will increase tremendously the interest of the manuscript for the brain evo-devo community.

The reviewer is right. Since progenitors positioned away from the apical zone, more basally, undergo mitosis in a less crowded environment, they should be less prone to mitotic errors when facing the same mitotic challenge than apical mitosis. This is a very attractive hypothesis. We propose to directly test this possibility by comparing mitotic fidelity of basal *versus* apical mitosis in E13.5 lateral tissue sections.

Minor concerns:

The authors use the terms early neurogenesis and late neurogenesis when referring to E13 and E16, respectively. However, in the field of developmental neuroscience early neurogenesis is typically referred to E10-E11. I advise the authors to change the phrasing, to make the classification more sound and less misleading to neurogenesis experts.

Yes, the reviewer is right. It was more like taking into consideration, earlier stage than E16.5. We will make these changes according to the reviewer' recommendation.

Paragraph 174-186 "Since MT polymerization activity is asymmetric between the two spindle poles, we plotted the data of the pole displaying the highest activity." Is the MT polymerization activity regulated proportionally at the two spindle poles? In other words, it would be interesting (using the data the authors gathered already) to understand if constraints are making the spindle more asymmetric. This is a basic tissue cell biology question the authors have the tools to address. I invite them to do so.

Indeed, we have already these data and we can include them in the manuscript. In fact, we observed that MT polymerization activity is regulated proportionally at the two spindle poles as we did not observe any significant change in spindle asymmetry.

Bibliography

Arai, Y., Pulvers, J. N., Haffner, C., Schilling, B., Nüsslein, I., Calegari, F., & Huttner, W. B. (2011). Neural stem and progenitor cells shorten S-phase on commitment to neuron production. *Nature Communications*, *2*(1). https://doi.org/10.1038/ncomms1155

Barroso-Vilares, M., & Logarinho, E. (2019). Chromosomal instability and pro-inflammatory response in aging. *Mechanisms of Ageing and Development*, *182*(January). https://doi.org/10.1016/j.mad.2019.111118

Calegari, F., Haubensak, W., Haffher, C., & Huttner, W. B. (2005). Selective lengthening of the cell cycle in the neurogenic subpopulation of neural progenitor cells during mouse brain development. *Journal of Neuroscience*, *25*(28), 6533–6538. https://doi.org/10.1523/JNEUROSCI.0778-05.2005

Caridi, C. P., D'agostino, C., Ryu, T., Zapotoczny, G., Delabaere, L., Li, X., Khodaverdian, V. Y., Amaral, N., Lin, E., Rau, A. R., & Chiolo, I. (2018). Nuclear F-actin and myosins drive relocalization of heterochromatic breaks. *Nature*, *559*(7712), 54–60. https://doi.org/10.1038/s41586-018-0242-8

Chugh, P., Clark, A. G., Smith, M. B., Cassani, D. A. D., Dierkes, K., Ragab, A., Roux, P. P., Charras, G., Salbreux, G., & Paluch, E. K. (2017). Actin cortex architecture regulates cell surface tension. *Nature Cell Biology*, *19*(6), 689–697. https://doi.org/10.1038/ncb3525

Iwashita, M., Kataoka, N., Toida, K., & Kosodo, Y. (2014). Systematic profiling of spatiotemporal tissue and cellular stiffness in the developing brain. *Development*, *141*, 3793–3798.

Kadowaki, M., Nakamura, S., Machon, O., Krauss, S., Radice, G. L., & Takeichi, M. (2007). N-cadherin mediates cortical organization in the mouse brain. *Developmental Biology*, *304*(1), 22–33. https://doi.org/10.1016/j.ydbio.2006.12.014

Krndija, D., Marjou, F. El, Guirao, B., Richon, S., Leroy, O., Bellaiche, Y., Hannezo, E., & Vignjevic, D. M. (2019). Active cell migration is critical for steady-state epithelial turnover in the gut. *Science*, *365*(6454), 705–710. https://doi.org/10.1126/science.aau3429

Lancaster, O. M., & Baum, B. (2014). Shaping up to divide: Coordinating actin and microtubule cytoskeletal remodelling during mitosis. *Seminars in Cell and Developmental Biology*, *34*, 109–115. https://doi.org/10.1016/j.semcdb.2014.02.015

López-Gay, J. M., Nunley, H., Spencer, M., di Pietro, F., Guirao, B., Bosveld, F., Markova, O., Gaugue, I., Pelletier, S., Lubensky, D. K., & Bellaïche, Y. (2020). Apical stress fibers enable a scaling between cell mechanical response and area in epithelial tissue. *Science*, *370*(6514). https://doi.org/10.1126/science.abb2169

Marthiens, V., & Basto, R. (2020). Centrosomes: The good and the bad for brain development. *Biology of the Cell*, 1–20. https://doi.org/10.1111/boc.201900090

Marthiens, V., Rujano, M. a, Pennetier, C., Tessier, S., Paul-Gilloteaux, P., & Basto, R. (2013). Centrosome amplification causes microcephaly. *Nature Cell Biology*, *15*(7), 731–740. https://doi.org/10.1038/ncb2746

McHugh, T., & Welburn, J. P. I. (2023). Potent microtubule-depolymerizing activity of a mitotic Kif18b–MCAK–EB network. *Journal of Cell Science*, *136*(5). https://doi.org/10.1242/jcs.260144

Nikalayevich, E., Letort, G., de Labbey, G., Todisco, E., Shihabi, A., Turlier, H., Voituriez, R., Yahiatene, M., Pollet-Villard, X., Innocenti, M., Schuh, M., Terret, M.

E., & Verlhac, M. H. (2024). Aberrant cortex contractions impact mammalian oocyte quality. *Developmental Cell*, *59*(7), 841-852.e7. https://doi.org/10.1016/j.devcel.2024.01.027

Orr, B., Talje, L., Liu, Z., Kwok, B. H., & Compton Correspondence, D. A. (2016). Adaptive Resistance to an Inhibitor of Chromosomal Instability in Human Cancer Cells. *CellReports*, *17*, 1755–1763. https://doi.org/10.1016/j.celrep.2016.10.030

Palumbieri, M. D., Merigliano, C., González-Acosta, D., Kuster, D., Krietsch, J., Stoy, H., von Känel, T., Ulferts, S., Welter, B., Frey, J., Doerdelmann, C., Sanchi, A., Grosse, R., Chiolo, I., & Lopes, M. (2023). Nuclear actin polymerization rapidly mediates replication fork remodeling upon stress by limiting PrimPol activity. *Nature Communications*, *14*(1), 1–15. https://doi.org/10.1038/s41467-023-43183-5

Perrin, L., Gannavarapu, V. R., Pérez-González, C., Rivera, C., Descroix, S., Lennon-Duménil, A. M., Bellaïche, Y., Krndija, D., & Matic Vignjevic, D. (2025). The Arp2/3 complex maintains gut epithelial integrity under mechanical challenge. *Current Biology*, *35*(19), 4827-4836.e5. https://doi.org/10.1016/j.cub.2025.08.026

Rosendahl, P., Plak, K., Jacobi, A., Kraeter, M., Toepfner, N., Otto, O., Herold, C., Winzi, M., Herbig, M., Ge, Y., Girardo, S., Wagner, K., Baum, B., & Guck, J. (2018). Real-time fluorescence and deformability cytometry. *Nature Methods*, *15*(5), 355–358. https://doi.org/10.1038/nmeth.4639

Rujano, M. A., Sanchez-Pulido, L., Pennetier, C., le Dez, G., & Basto, R. (2013). The microcephaly protein Asp regulates neuroepithelium morphogenesis by controlling the spatial distribution of myosin II. *Nature Cell Biology*, *15*(11), 1294–1306. https://doi.org/10.1038/ncb2858

Shi, L., Qalieh, A., Lam, M. M., Keil, J. M., & Kwan, K. Y. (2019). Robust elimination of genome-damaged cells safeguards against brain somatic aneuploidy following Knl1 deletion. *Nature Communications*, *10*(1), 1–14. https://doi.org/10.1038/s41467-019-10411-w

Stout, J. R., Yount, A. L., Powers, J. A., LeBlanc, C., Ems-McClung, S. C., & Walczak, C. E. (2011). Kif18B interacts with EB1 and controls astral microtubule length during mitosis. *Molecular Biology of the Cell*, *22*(17), 3070–3080. https://doi.org/10.1091/mbc.E11-04-0363

Taubenberger, A. V., Baum, B., & Matthews, H. K. (2020). The Mechanics of Mitotic Cell Rounding. *Frontiers in Cell and Developmental Biology*, *8*(August), 1–16. https://doi.org/10.3389/fcell.2020.00687

Toyoda, Y., Cattin, C. J., Stewart, M. P., Poser, I., Theis, M., Kurzchalia, T. V., Buchholz, F., Hyman, A. A., & Müller, D. J. (2017). Genome-scale single-cell mechanical phenotyping reveals disease-related genes involved in mitotic rounding. *Nature Communications*, *8*(1), 1–10. https://doi.org/10.1038/s41467-017-01147-6

Vargas-Hurtado, D., Brault, J. B., Piolot, T., Leconte, L., Da Silva, N., Pennetier, C., Baffet, A., Marthiens, V., & Basto, R. (2019). Differences in Mitotic Spindle Architecture in Mammalian Neural Stem Cells Influence Mitotic Accuracy during Brain Development. *Current Biology*, *29*(18), 2993-3005.e9. https://doi.org/10.1016/j.cub.2019.07.061

Wang, P. S., Chou, F. S., Ramachandran, S., Xia, S., Chen, H. Y., Guo, F., Suraneni, P., Maher, B. J., & Li, R. (2016). Crucial roles of the Arp2/3 complex during mammalian corticogenesis. *Development (Cambridge)*, *143*(15), 2741–2752. https://doi.org/10.1242/dev.130542

Dr. Renata Basto
Institut Curie
UMR144
12 rue Lhomond
Paris 75005
France

16th Dec 2025

Re: EMBOJ-2025-122764
Brain biomechanics governs mitotic fidelity of embryonic neural stem cells

Dear Renata and Veronique,

Thank you again for your detailed point-by-point response letter and for discussing the referee reports and revision plans with me. I appreciate your responses and was happy to see that you seem to be in a good position to address the recurrent key issues I had noted in my previous message.

I would therefore be happy to invite you to revise the study very much as proposed in your letter, and to resubmit it to The EMBO Journal using the link below. It is our policy to allow only a single round of (major) revision, so please keep me updated should there be any unexpected problems with the revisions, or should you require an extension beyond the default 3-months deadline. As always, competing manuscript published elsewhere during the course of this revision will not affect our eventual decision on your study. Finally, please note the detailed information and guidelines on how to prepare a revision below (and in our online Guide to Authors) - closely adhering to them shall greatly facilitate the editorial process at the time of resubmission.

Thank you again for the opportunity to consider this work, and I look forward to receiving your revision in due time.

With kind regards,

Hartmut

Hartmut Vodermaier, PhD
Senior Editor, The EMBO Journal
h.vodermaier@embojournal.org

*** PLEASE NOTE: All revised manuscript are subject to initial checks for completeness and adherence to our formatting guidelines. Revisions may be returned to the authors and delayed in their editorial re-evaluation if they fail to comply to the following requirements. As a first step please read our guidelines for revised submissions:
https://link.springer.com/journal/44318/submission-guidelines#cms-Revised-submissions

1) Every manuscript requires a Data Availability section (even if only stating that no deposited datasets are included). Primary datasets or computer code produced in the current study have to be deposited in appropriate public repositories prior to resubmission, and reviewer access details provided in case that public access is not yet allowed.

2) Each figure legend must specify
- size of the scale bars that are mandatory for all micrograph panels
- the statistical test used to generate error bars and P-values
- the type error bars (e.g., S.E.M., S.D.)
- the number (n) and nature (biological or technical replicate) of independent experiments underlying each data point
- Figures may not include error bars for experiments with n<3; scatter plots showing individual data points should be used instead.

3) Revised manuscript text (including main tables, and figure legends for main and EV figures) has to be submitted as editable text file (e.g., .docx format). We encourage highlighting of changes (e.g., via text color) for the referees' reference.

4) Each main and each Expanded View (EV) figure should be uploaded as individual production-quality files (preferably in .eps, .tif, .jpg formats). For suggestions on figure preparation/layout, please refer to our Figure Preparation Guidelines:
https://media.springernature.com/original/springer-cms/rest/v1/content/27825798/data/v1

5) Point-by-point response letters should include the original referee comments in full together with your detailed responses to them (and to specific editor requests if applicable), and also be uploaded as editable (e.g., .docx) text files.

6) Please complete our Author Checklist, and make sure that information entered into the checklist is also reflected in the manuscript; the checklist will be available to readers as part of the Review Process File.

7) All authors listed as (co-)corresponding need to deposit, in their respective author profiles in our submission system, a unique ORCiD identifier linked to their name. Please see our Guide to Authors for detailed instructions.

8) Please note that supplementary information at EMBO Press has been superseded by the 'Expanded View' for inclusion of additional figures, tables, movies or datasets; with up to five EV Figures being typeset and directly accessible in the HTML version of the article.

9) To facilitate reproducibilty and cross-laboratory adoption of methodologies, please structure the Materials & Methods section as outlined in our guide to authors, including a completed Reagents and Tools Table.

10) Digital image enhancement is acceptable practice, as long as it accurately represents the original data and conforms to community standards. If a figure has been subjected to significant electronic manipulation, this must be clearly noted in the figure legend and/or the 'Materials and Methods' section. The editors reserve the right to request original versions of figures and the original images that were used to assemble the figure. Finally, we generally encourage uploading of numerical as well as gel/blot image source data.

At EMBO Press we ask authors to provide source data for the main manuscript figures. You will receive a separate email with instructions for providing source data with your revised manuscript, including how to upload and organize the files.

In the interest of ensuring the conceptual advance provided by the work, we recommend submitting a revision within 3 months (16th Mar 2026). Please discuss the revision progress ahead of this time with the editor if you require more time to complete the revisions.

Reponses to reviewers of EMBOJ-2025-122764, Marthiens et al

We acknowledge the reviewers for their insightful comments, suggestions and questions.

Referee1:

The causal connection between mechanical compression, actin cortex remodeling, MCAK regulation, and chromosome mis-segregation remains largely inferential.

We understand this reviewer's point of view. While our study connected mechanical compression, actin cortex remodeling and chromosome mi-segregation, it did not establish an obvious causal link between the three points referred above. After the revision however, we now provide new data to re-enforce these causal relationships as detailed below.

1. Demonstrate more directly that cortical actin tension modulates MCAK activity or microtubule polymerization. This could be achieved by monitoring MCAK localization or phosphorylation under compression or actin perturbation, or by testing whether MCAK overexpression rescues mis-segregation phenotypes.

We did exactly what this reviewer requested in the revised version of our manuscript. We show that MCAK localization, as a readout of its activity changes in different settings. First, we observed that the fraction of centromeres positive for MCAK increases when sustaining its activity upon treatment with the UMK57 agonist, indicating that the loading of MCAK at the centromeres is a good indicator of its activity (new panels Figure EV3G-H). Second, we show that decreasing cortical actin after CK666 drug treatment in 3D aRG cultures, is sufficient to increase the fraction of kinetochores positive for MCAK (new panels Figure 5M-N), indicating that MCAK activity is increased upon reorganization of branched actin. Third, and more importantly, we report that in the cerebral cortex, the fraction of centromeres positive for MCAK is higher at E16.5, as compared with E13.5 in aRG cells (new panels Figure 6G-H), at the stage when mitotic cells are less confined by their neighbors. Fourth, in conditions where MCAK activity is sustained (in the presence of the UMK57 agonist), release of cortical tension upon CK666 treatment does not influence MT polymerization rates (new panels Figure 5O and EV6L), nor chromosome segregation (new panels Figure 5P and EV6M-N) frequency. The same was true when the same experiment was performed after Latrunculin (Lat) B, instead of CK666 treatment (Figure EV7A-D). Altogether, these results further strengthen a role for cortical actin in modulating MCAK activity and consequently spindle pole MT polymerization rates and chromosome mis-segregation.

2. The pharmacological manipulations (latrunculin B, CK666, dextran) alter multiple cellular processes and thus cannot unambiguously assign causality to actin-mediated tension.

This reviewer is right, of course. Any drug treatment will alter several cellular processes on a long-term basis. The same effect is expected for any genetic manipulation of members of the actin cytoskeleton. Before each set of experiments, we chose the lowest dose to be used to decrease in a quantitatively detectable manner actin recruitment at the plasma membrane and cortical membrane stiffness (LatB, Figure EV5D-F, Figure EV7E), to delocalize the Arp2/3 complex from the plasma membrane (CK666, Figure 5H-I, Figure 6A-B, 6E) or to perturb mitotic cell shape (Dextran, Figure 4A-B, H) in E13.5 mouse aRG primary cell cultures. Importantly, we have controlled that the chosen doses were not impairing aRG mitotic progression after evaluation of the time elapsed between nuclear envelope breakdown and anaphase onset (mitotic timing). We have now included time-lapse images and mitotic timing quantification to show the absence of changes in mitotic progression when aRGs were treated with LatB (new panels EV5H-I), CK666 (new panels EV6F-G), or dextran (EV4C, new panel EV4G) alone. Moreover, as mentioned above, all the functional assays were performed after a short drug exposure or incubation with dextran, i.e., 1 hour for microtubule depolymerization-repolymerization assays and 2.5 hours for time-lapse recording. Such short-time windows aimed at restricting our analysis to cells in preparation for mitosis. The G2 phase length has been previously evaluated to be around 2 hours in aRG cells at this developmental stage (Arai *et al*, 2011) and so this short period of time ensures a minimal potential side effect. It is well known that nuclear actin plays an essential role in DNA damage response (Caridi *et al*, 2018; Palumbieri *et al*, 2023), and we did not want to interfere with this process, which can ultimately lead to indirect mitotic errors.

Additionally, even if compressive forces were mimicked with different approaches (PDMS devices in Figure 3A-E or incubation with Dextran in Figure 4), the same overall outcome in 2D aRG cultures - increase in microtubule polymerization activity from the spindle poles- was noticed. More importantly, while LatB and CK666 treatments perturb both microtubule polymerization capacity from the spindle poles and mitotic fidelity in 3D setting, we did not observe any effect in aRG cultures plated on 2D substrates (Figure EV6B-E for Lat B or Figure EV6J-K for CK666). The lack of effect in 2D attests for a lack of perturbations that could contribute to mitotic phenotypes in our experimental settings. Altogether, these observations strongly argue in favor of compressive forces conveyed by ARP2/3-dependent branched cortical actin at the origin of changes in spindle pole microtubule polymerization activity and chromosome segregation.

We do not think that a genetic perturbation is ideal here. We require an acute, fast way of decreasing actin polymerization activity. Taking into consideration that cells lacking actin or with low actin levels will undergo cell shape changes and more importantly will fail cytokinesis (Lancaster & Baum, 2014; Taubenberger *et al*, 2020), we would be in a position where any type of conclusion would not be possible to

ascertain to a role of cortical actin at mitotic entry. In other words, we would be studying a cumulative effect of actin depolymerization all along the cell cycle.

We have explained better this rationale when introducing the actin depolymerization experiments in the text. It is also worth mentioning that we did try to analyze a conditional ARPC4 knockout mouse model (Arpc4<tm1a(EUCOMM)Wtsi> Targeted Allele Detail MGI Mouse (MGI:4433308), but similarly to what has been published in other tissues (Perrin *et al*, 2025) and in the mouse brain (Wang *et al*, 2016), this lead to loss of epithelial tissue integrity (Figure 1 below). The brains from these mice were extremely soft, disrupted and difficult to handle. For these reasons, we decided to use alternative short-term pharmacological approaches in order to prevent long-term cumulative side-effects consequences of tissue dysplasia.

-

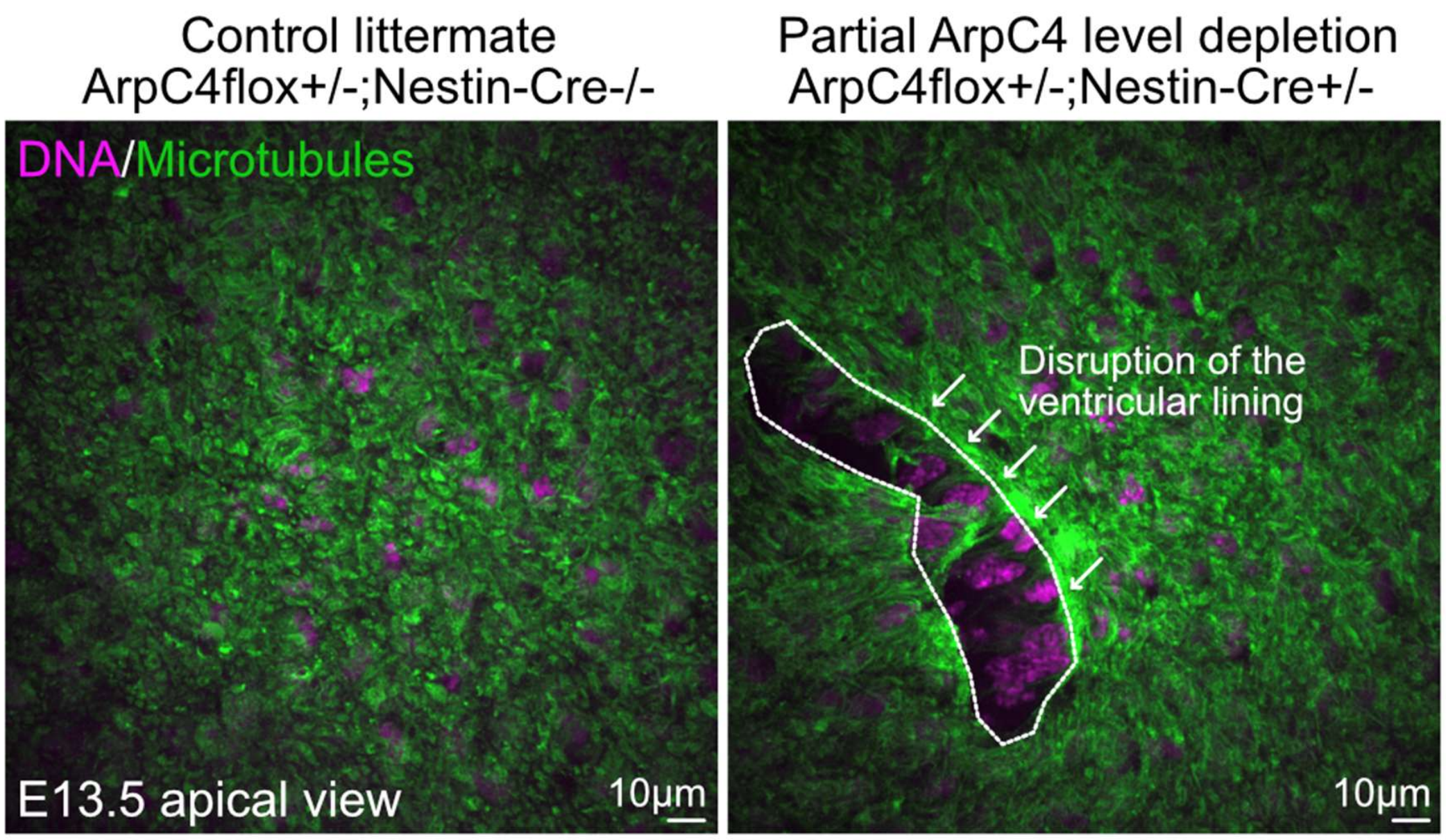

Figure 1: En face view of the apical side of the neuroepithelium that shows disruption of the ventricular lining in the E13.5 neocortex after partial depletion of the ArpC4 subunit (part of the ARP2/3 branched actin nucleation complex) in comparison with control littermate.This is illustrated by abnormal detachment of aRG apical processes (in green) and apparent mispositioning of interphase nuclei and mitotic chromosomes (DNA in magenta)

3. Mechanical measurements rely mainly on relative recoil velocities after laser ablation, which do not quantify actual stress magnitudes; complementary methods such as AFM, would substantiate the mechanical framework.

Provide absolute measurements of tissue or cell mechanical stress (e.g., AFM indentation, microindentation, or optical tweezers) to support claims about compression and cortical tension.

It is true that our approaches provide relative measures. But this is the case of many studies performed in tissues such as (Krndija *et al*, 2019; López-Gay *et al*, 2020; Rujano *et al*, 2013). They certainly do not replace absolute measures, but they provide

with a basis to compare cortical tension within different conditions. We chose this strategy because in the mouse developing brain, the use of atomic force microscopy (AFM) in different regions at different developing stages showed that tissue stiffness could not be explained by the stiffness of the cells that constituted the tissue (Iwashita *et al*, 2014). We are therefore convinced that the measurements of stiffness performed by AFM cannot reflect an absolute measurement of cortical tension of individual cells, but instead reflects the contribution of multiple parameters, including the type of microtubule cytoskeleton and properties, extracellular matrix composition, which are outside of the scope of our study. Even if not an absolute measure, relative differences in recoil velocity after laser ablation is to our knowledge the only quantitative approach used at the scale of a tissue that reflect an evaluation of actin-dependent cortical tension of individual cells (Krndija *et al*, 2019; López-Gay *et al*, 2020). These experiments shown in our study were very difficult to perform and were controlled to use different samples at the same day with the same laser conditions. In our lab, we have also made huge efforts to use optical tweezers to probe cell mechanical properties. After weeks and weeks of attempts, we decided that these were not compatible with *in vivo* experiments in tissues from different model organisms.

We understand that the referee wishes to be more convinced regarding the link between compression and actin-based cortical tension *in vivo*. We have a clear correlation between increase in cell density (Figure 1D-F), actin recruitment at the plasma membrane (Figure 5A-C) and cortical membrane tension after velocity recoil measurement (Figure 5D-F), when comparing E13.5 and E16.5 aRG in mitosis. Individual mitotic cell size (Figure 1F), shape (Figure 1G-I) and volume (Figure 1J) are also directly related to differences in cell density in the ventricular zone. To further re-enforce this point, we have also linked the position of mitotic aRGs within the 3D neurospheres with increased chromosome mis-segregation when compared to cells positioned at the periphery (new panel Figure EV2H). As the *ex vivo* Dextran experiments (Figure 4 and EV4) support the view that imposing mechanical compressive forces of 10kPa to individual mitotic cells is sufficient to mimic the *in vivo* changes in terms of microtubule polymerization activity and mitotic error frequency, we favored the interpretation that compressive forces imposed by cell density is a critical parameter *in vivo*.

We do not think that it is experimentally possible to link cell density and compressive forces *in vivo.*

4. In addition, developmental comparisons between E13.5 and E16.5 might reflect biochemical and metabolic differences beyond mechanics, and the authors should demonstrate that changes in mitotic fidelity are not secondary to other developmental factors.

Developmental specificity. Control for biochemical differences between E13.5 and E16.5 stages (cell cycle duration, polarity markers) to confirm that observed effects truly derive from mechanics.

This is a very important point and we now show, thanks to the reviewer's questions, that aRG cells are mechanosensitive in a restricted period during development. Indeed, we have performed Dextran experiments to reimpose the same level of mechanical stress (10kPa) and compared in a quantitative manner E13.5 and E16.5 aRG primary cell culture potential regarding chromosome segregation (new panels Figure 4H-I and EV4F-G). We have evaluated after time-lapse recording of 2D aRGs that chromosome mis-segregation was 3.4X more frequent upon dextran- imposed compressive forces at E13.5, as compared to 1.3X at E16.5 (new panel Figure 4I). This implies that aRG cells progressively lose their capacity to be influenced by compressive stress imposed by cell density, while progressing in their developmental lineage. In parallel, we have also evaluated that the activity of MCAK during mitosis is higher in the cerebral cortex at E16.5, as compared to E13.5, as indicated by a marked increase in the loading of MCAK on centromeres (new panels Figure 6G-H). As MCAK contributes to error-correction mechanisms at kinetochores (Barroso-Vilares *et al,* 2020), we propose that such endogenous increase of MCAK activity protects E16.5 aRGs from chromosome segregation errors *in vivo*. We have now discussed implications of such differences regarding the mechanosensitivity of E13.5 and E16.5 aRGs in the discussion section.

Altogether, based on the data provided in the manuscript, we are confident that spindle pole microtubule polymerization and mitotic fidelity can be tuned by cell density-related stress in the first half of neurogenesis, when aRGs are confined in the VZ because of their high proliferation rates. It is not our intention to say that it is the only factor that contributes to mitotic fidelity, but instead one new parameter to be considered in tissues, which has never been reported before. We have already shown differences in the loading of the microtubule-associated protein TPX2 on spindle microtubules between E13.5 and E16.5 mitosis responsible for changes in spindle morphology and related mitotic fidelity (Vargas-Hurtado et al., 2019). The Marthiens et al. 2026 manuscript shows for the first time that biomechanics is another parameter that must be considered in the regulation of mitotic fidelity in the developing brain. Whether differences in mechanosensitivity and TPX2 loading are mechanistically linked will have to be investigated in the future.

To answer the reviewer's question regarding biochemical differences between the two stages, decrease in cell cycle length and polarity have been reported during neurogenesis along with neural cell fate commitment (Arai *et al*, 2011; Calegari *et al*, 2005; Arai & Taverna, 2017). We therefore expect changes in aRG cell cycle lengths and polarity markers between E13.5 and E16.5. Regarding cell polarity, it is important to mention that aRG cells cultured as floating spheroids (Figure EV2A-B) lack polarity. As release of mechanical tension by actin cytoskeleton perturbation have the same impact on microtubule polymerization and mitotic fidelity in 3D aRG cultures (absence of polarity) and cortical explants (polarity preserved), we considered that polarity is not a main contributor. We have discussed this important point in the discussion.

5. The in vivo validation is also limited: drug treatments of explants are short term and lack controls for potential cytotoxic or off-target effects.

Short treatments of cortical explants (no more than 3 hours with the drugs) were performed with the intention to avoid long-term cytotoxic effects of the pharmacological approaches used, while still perturbing cortical actin levels and/or organization (Figure 6A, 6B, 6E for CK666 and EV7E and EV7F for LatB) for immediate functional analysis of mitosis. With this experimental design, we already noticed neuroepithelium dysplasia, including disruption of the ventricular lining illustrated by disorganization of the actin cytoskeleton at the apical side of the ventricular zone (for both actin drugs, Figure 6E and EV7E), mis-positioning of aRG nuclei in the ventricular zone (Figure EV7F, LatB), alterations of mitotic cell shapes (Figure 6B, CK666) or even delay/lack of furrow ingressions (Figure 6E, CK666). Lack of furrow ingressions might for example increase the probability of cytokinesis failure and results in the generation of polyploid daughter cells (that did not undergo final cell division). Please refer to our discussion on the experimental choices for acute and short-term pharmacological treatments in point 2.

Extend analysis beyond short-term explant assays, ideally through mechanical relaxation or local actin modulation in intact embryos, to establish physiological relevance.

Demonstrating the modulatory effect of mechanobiology in an intact tissue is extremely challenging in a mammalian organ. Indeed, the actin cytoskeleton is involved in many processes, including the capacity of cells to interact in tissues. Perturbing its integrity on a long-term scale leads to an extreme disorganization of the tissue, i.e., global or local dysplasia, as already observed after adhesion molecule loss-of-function (N-cadherin KO mouse model to cite an example (Kadowaki *et al*, 2007)), and as observed after partial Arp2/3 loss-of-function in our hands (see discussion in point 2). These side effects after genetic perturbation of actin cytoskeleton on tissue integrity prevented us from long-term analysis.

6. Statistical transparency. Include full reporting of biological replicates, sample numbers, and variance for all quantitative data.

The full data sheet with all the information is provided with the revised version of the manuscript.

7. Physiological significance. Discuss to what extent the measured two-fold increase in lagging chromosomes can impact neurogenesis and brain size, integrating these effects into broader developmental or disease contexts such as microcephaly.

We have discussed these points more extensively in the discussion section.

Minor points

1. Clarify terminology, phrases such as "cortical tension acts as a sensor" are misleading; tension is a consequence, not a sensor.

We have corrected this terminology and replaced it by conveyor.

2. Improve figure legends with clearer statistical annotations and scale bars; some panels lack units or replicate information.

The Figures and figure legends have been changed accordingly.

3. Simplify and reorganize the discussion to separate descriptive results from speculative interpretation; the current flow is somewhat repetitive.

We made these changes in the organization of the discussion.

4. Indicate whether drug treatments (LatB, CK666) affected cell viability or mitotic progression independently of mechanical changes.

We used the lowest doses for these drugs to see an effect and did not notice any change in cell viability above control conditions or in mitotic timing (from metaphase to anaphase), as now reported in new panels Figure EV5H-I for LatB and EV6F-G for CK666. It worth noticing that the concentration of drug to be added in the medium was chosen according to the absence of such side effects. It is also important to state that all our experiments were performed in a short time window (less than 3 hours) as explained above and in the main text.

5. Provide higher-resolution images for EB1 comet analyses and ensure that quantification criteria (thresholds, region selection) are transparent and reproducible.
We now provide higher resolution images for EB1 comet analysis (Figure EV3A). The Fiji plugin for analysis of EB1 comets included manual correction options. For example, it included the possibility to draw the pole of interest, the region of interest around each pole, to add non detected comets, remove wrongly detected ones…we provide the different steps of the procedure for better evidence of transparency.

6. Adjust color schemes throughout Figure to colorblind-friendly palettes (e.g., magenta-cyan or orange-blue instead of red-green) to ensure accessibility and improve contrast for printed and digital formats.

We made these color changes for all the Figures.

Referee 2:

Introduction:

The introduction is very concise at places and rationales and arguments are not always fully worked out. For example, it could be better explained why the cortex would be more sensitive to chromosome segregation errors and more failed mitoses than other highly dividing tissues, including other epithelia, in the developing organism. Line 60-61, the types of tissue remodeling referred to should be at least briefly

mentioned as should the fact that neurogenesis proceeds even after birth in many vertebrates including mammals.
Line 66-67, the rationale for studying 'biomechanics' in mitosis could be better worked out.

We have explained better the rationale behind our study by rewriting the introduction. Neurogenesis in the cerebral cortex proceeds in great majority before birth in mouse, even if neuron production persists post-natally in restricted regions, i.e. the hippocampus and the sub-ventricular zone lining the ventricle. Because of time restriction and challenge to produce enough neurons to populate the different cortical layers, the rate of proliferation and therefore cell density in the ventricular zone is higher than in other regions of the brain and epithelia of the organism. We wanted to evaluate a potential causal relationship between cell density and mitotic defects in this study.

General logic:
- If detrimental for development at times, it is not clear why such biomechanical sensitivity persists over evolution in normal brain development. Conceptually, one would question why such sensitivity would persist. The authors could expand on that logical argument.

This is a very good point and we have added to the discussion section. In the revised version of the manuscript, we now show that aRGs dissociated from E16.5 cerebral cortex are less mechanosensitive when compared to E13.5 aRGs (new panels Figure 4H-I and EV4F-G). In addition, we now show that chromosome segregation errors in progenitors that reside in the ventricular zone, the aRGs (also called apical progenitors, APs) is increased when compared to progenitors that populate the sub-ventricular zone from the same region of the dorsal telencephalon at the same developmental stage -E13.5 (new panels Figure 2C-D). Our view is that this mechano-sensitivity is primarily required to adjust the rate of MT polymerization from the spindle poles and maintain the population of astral MTs for proper positioning of aRG cells in the neuroepithelium. Failure to do so will result in dysplasia of the tissue and disorganized cortical layering at birth. Nonetheless, a drawback of this mechano-sensitivity and high rate of MT polymerization is an increased probability to generate mitotic errors. As safeguard mechanisms exist to detect and eliminate unfit cells from the cycling population (by apoptosis or premature neuronal differentiation (Lizarraga *et al*, 2010; Marjanović *et al*, 2016; Marthiens *et al*, 2013; Rujano *et al*, 2013; Shi *et al*, 2019; Phan *et al*, 2021; Viais *et al*, 2021; Gogendeau *et al*, 2015), the consequences on brain size are undetectable. It is only in pathological conditions that this vulnerability is unraveled with consequences on brain size and functionality.

Along the experimental outlines, it is slightly counterintuitive how interference with the actin cortex and thereby cortex-spindle interactions lead to less chromosome segregation errors. This could be further discussed (see also below).

This is also a very interesting discussion point that we have included. It seems indeed that the majority of cortex-spindle interactions enabled by increased MT

polymerization from the spindle pole comes at the expense of the efficiency of chromosome capture and/or error correction mechanisms. The MCAK microtubule-depolymerase activity playing a dual role in modulating MT polymerization from the spindle pole and in error correction, may explain mechanistically this interdependence. This possibility is highly fueled from our fundamental knowledge of MCAK mechanistic role in the literature and by our new findings included in the revised version of the manuscript and detailed below.

Experimental points:

- Ideally, crowding of the tissue should also be shown along the apicobasal axis to underline the statement that mitotic crowding is highest at early stages in the mitotic zone. While 3D analysis is probably too tedious, some 2D analysis using Cellpose or similar tools from apical to basal could be performed.

As requested by this reviewer, we now provide new data (Figure 1B) showing higher density of apical progenitor (AP) mitosis in the ventricular zone at E13.5 as compared to the higher density of basal progenitor (BP) mitosis in the sub ventricular zone at E16.5 in lateral sections of the cerebral cortex, with corresponding evaluation of the areas occupied by both regions (Figure 1C). Additionally, as required by this reviewer, we performed 2D analysis of nuclear shapes as a readout of the level of biomechanical constraints along the apico-basal axis (new panels Figure 2A-B). We segmented the nuclei after Cell Pose on 2D sections, identified Apical progenitors *versus* Basal progenitors nuclear fate after Sox2 staining and evaluated nuclear shapes with aspect ratios. These new panels illustrate the higher burden of constraints of Apical progenitors as compared with Basal progenitors at early stages.

- Fig. 1F, some raw data on the analysis of spindle morphology should be added to the supplement.

Figure 1F displays comparison of cell volumes after segmentation in the E13.5 and E16.5 VZ. As we have already thoroughly analyzed changes in spindle morphology at these two stages of development and provided quantitative analysis (Vargas-Hurtado *et al*, 2019a), we understand that the referee wishes that we include images of spindle morphology related to cell volume. We now provide new panels (Figure EV1A) of ventricular zone *en face* views depicting differences in spindle morphology related to cell size between the two stages. In agreement with our previous study, spindle microtubule density (in green) and mitotic cell area are both lower in mitotic aRG at E13.5 than at E16.5, whereas the population of astral microtubules contacting the cell cortex (illustrated by increased NuMA at the plasma membrane) is enriched.

- In Figure 2, the analysis pipeline of lagging chromosomes should be better explained and documented. The presented images look very blurry and it is unclear how scoring can be made reliable and reproducible over different culture conditions.

We have provided better images to illustrate the procedure for lagging chromosome scoring in new panel Figure 2H. We have also provided more explanations in the

Method section to better explain the criteria used to evaluate chromosome missegregation frequency.

Overall, some comment on the fates of these lagging chromosomes should be made as often these can still be caught and integrated into the spindle without causing aneuploidy. Maybe some estimation of the number of aneuploid cells in different conditions could be given.
The referee is right. There is one chance out of two that the merotelic attachment (shown now in panel EV2C) results in an error in chromosome segregation, i.e., generates two aneuploid cells. When scoring higher frequency of lagging chromosomes, we therefore score higher probability of mitosis to generate aneuploid progeny. These correlation between merotelic attachments, lagging chromosomes and aneuploidy are well documented in the literature and we have explained better and discussed them in the result and discussion sections.

- The link between the increase of astral microtubules and chromosome segregation errors could be better explained.
We provide in the revised version of the manuscript more data regarding the modulatory effect of cortical actin on MCAK activity. We show that MCAK activity (using MCAK loading on the kinetochores as a readout, new panel Figure EV3G-H) is potentiated after CK666-induced cortical actin remodeling (Figure 5M-N). We also show that MCAK levels at the centromere are higher at E16.5 (new panel Figure 6G-H), when astral microtubules -cortex interactions are less frequent (new panel Figure EV1-reduced NuMA levels at the cortical membrane of E16.5 mitotic aRGs as a readout of instructive interactions between astral microtubules and the plasma membrane). It appears therefore that cortical actin influences the recruitment of factors important for spindle positioning and interactions of the plasma membrane with astral microtubules is also an upstream regulator of MCAK activity, which regulates at the same time spindle pole microtubule polymerization activity and correct microtubule-kinetochore attachments. We have discussed these points better.

- Some, even qualitative, in vivo data on the rate of MT polymerization at the spindle poles would help the authors' arguments in addition to the ex vivo data presented.

Microtubule depolymerization-repolymerization assays remain the best functional quantitative evaluation of microtubule polymerization properties, which justify short-term (1 hour maximum) *ex vivo* experiments. We have used EB1 comet number and distance from the spindle poles as an evaluation of microtubule polymerization parameters. If EB1 comet parameters can be analyzed in the tissue after 1hour long microtubule depolymerization on ice and 3 minutes-long cumulative repolymerization events, the same parameters are difficult to extract at steady-state in a quantitative manner. To help the reader and as suggested by this reviewer, we have included a new panel (Figure EV4E) displaying comparative distribution of EB1 comets within the

E13.5 and E16.5 spindles. Actively polymerizing EB1-positive microtubules are enriched in the E13.5 spindles, as compared with E16.5.

- When it comes to the upregulation of cortical actin upon compression, what do the authors mean by 'sensor'? Would it not be possible that this is a tissue/cellular reaction to stiffening of the cortex in a stiffer environment that feeds back on all cells?
This point was also brought up by another reviewer. We will explain this better. We meant to state that the increase in branched cortical actin may be due to the response of mitotic cells to increase in cell density in their environment. We have removed the notion of sensor, which was misleading, and replaced it by "conveyor", which we believe to be more appropriate. We suspect that when aRG cells need to round-up for mitotic entry, they reorganize their cortical actin to accommodate enough intracellular space for spindle assembly and chromosome alignment. When aRGs face a higher load of mechanical constraints, as is the case at E13.5 in the ventricular zone, they increase enough cortical actin polymerization activity to achieve this task. It might not be perfect, as illustrated by the more elongated shape of aRG mitosis at this stage (Figure 1G-H), but still sufficient for spindle assembly and chromosome capture and segregation. We have discussed this model in the revised version of the manuscript.

- What do the authors speculate is the nature of the spindle-astral microtubules-cortex interplay and how is this related to the tissue crowding?
Factors that are important to regulate the interactions between plasma membrane and astral microtubules for spindle positioning (active form of ERM proteins, FigEV5A-C or NuMA, new panel Figure EV1A-B) are enriched at earlier stages of neurogenesis, when cell crowding is higher. Our interpretation is that increase in cell-cell interactions upon cell crowding (as mimicked by Ncadherin-Fc coating on the lateral sides of the PDMS grooves in Figure 3A-E) favors astral microtubule stabilization at the plasma membrane, but not microtubule polymerization from the spindle poles *per se* (Figure EV3D-F). Instead, accumulation of branched cortical actin at the plasma membrane initiates a yet-to be characterized signal that culminates in microtubule polymerization from the spindle poles.

Minor points:

Nowadays red/green annotated Figure are no longer widely used in the field to make figure interpretation easier for people with compromised colour vision.
We made these color changes for all the Figures.

- The graphs, while correct, are on first sight not very intuitive. It might be more intuitive to plot all data points (not just outliers) or use methods like violin plots. It is at the authors' discretions though.
The graphs used in this paper were chosen to visualize both median and variance. When comparison between medians was clear enough, we have plotted all data points, otherwise we kept the initial graphs.

- Figure S2F, it seems that in this experimental set-up lagging chromosomes are slightly more abundant in the 2D than in the 3D condition in the untreated scenario. Do the authors have an explanation for this?
The referee has righteously noticed the fact that DMSO anhydrous treatment alone increases the frequency of lagging chromosomes in 2D cultures, but not in 3D cultures, as compared to the control situation. However, this is not statistically significant, and the differences are quite small – less than 2%. We think they may just reflect experimental variations.

- Line 102, it is not clear what is meant by 'differences in mitotic fidelity'.
We have rephrased this sentence. Our previous work has demonstrated higher frequency of lagging chromosomes at E13.5 as compared to E16.5 after mitotic challenge with Mps1 or CENPE inhibitor of cerebral cortex explants. These differences between the two stages were referred to as “differences in mitotic fidelity”.

- As the authors do not really measure 'mechanical forces at play' except in the laser ablation experiments, the language of what is measured and what a possible interpretation should be adapted.
The referee is right. We cannot infer force measurement from our experimental data. We have indeed measured relative differences in cortical tension in relation with cell density, we have corrected this terminology.

Referee 3:

1) The authors leverage and heavily rely on pharmacology to dissect the involvment of several pathways and molecular players. Although the choice is understandable from an experimental point of view, considering the complexity of the developing brain tissue and the possibility of drug's side effects and/or secondary effects involving cell types other than aRGs, it is necessary to corroborate the main findings using also a genetic manipulation (via elecroporation of specific shRNAs, e.g.). I am referring in particular to the data presented in the paragraphs "Cortical actin senses biomechanical compression in mitotic aRG cells", that are of great relevance for the precise identification of actin as the main molecular pathway involved via the manipulation of Arp2/3. The same applies to the demonstration of actin working upstream of MCAK (in "Cortical actin acts upstream of MCAK to tune polar MT polymerization and modulate chromosome segregation errors"). Again, it would be important to corroborate the data obtained via pharmacological manipulation with a genetic manipulation as well.
The genetic manipulation will make the findings more convincing and related conclusions stronger.
We understand that this reviewer prefers genetic approaches. So do we, as after all, we are a genetics lab, which has done many studies using fly genetics. But

unfortunately, we tried to do *in vivo* genetic approaches without success. As described previously, and presented in Figure 1 above, we used the ARPC4 conditional mouse model, which has been used to study the link between cortical membrane tension and chromosome segregation in mouse oocytes (Nikalayevich *et al*, 2024). However, when we analyzed the ventricular zones, we observed a disruption of the ventricular lining and tissue dysplasia, a disorganization already reported in the literature after ARP2/3 loss-of-function (Wang *et al*, 2016). This is exactly why we had to use acute drug perturbation assays with whole dorsal telencephalon of preserved morphology for short-term scale analysis (Figure 6). Reviewer 1 also raised this point. Please read above as well.

In order to provide an alternative approach to the use of UMK57, we have also attempted to manipulate the intracellular levels of MCAK by over-expressing the construct containing MCAK fused with GFP (pYOY152 plasmid from Linda Wordeman (Addgene plasmid # 13987; http://n2t.net/addgene:13987 ; RRID:Addgene_13987)). This proved to be extremely toxic for aRG cells in culture. Indeed, 12 hours post-electroporation we detected very high levels of apoptosis through the presence of pyknotic nuclei. We then tried a new approach by electroporating low plasmid amounts (200ng) for a short period of time (4 to 6 hours post-transfection) (Figure 2). But even in these conditions, we could hardly detect any cell in mitosis. Moreover, the reduced number of cells that were in mitosis were arrested in prometaphase, and we could not observe cells in anaphase. We concluded that we cannot manipulate MCAK activity in this way in these cells, owing to obvious side-effects on mitotic progression, as already reported in the literature after experimental manipulation of MCAK levels (Wordeman *et al*, 2025).

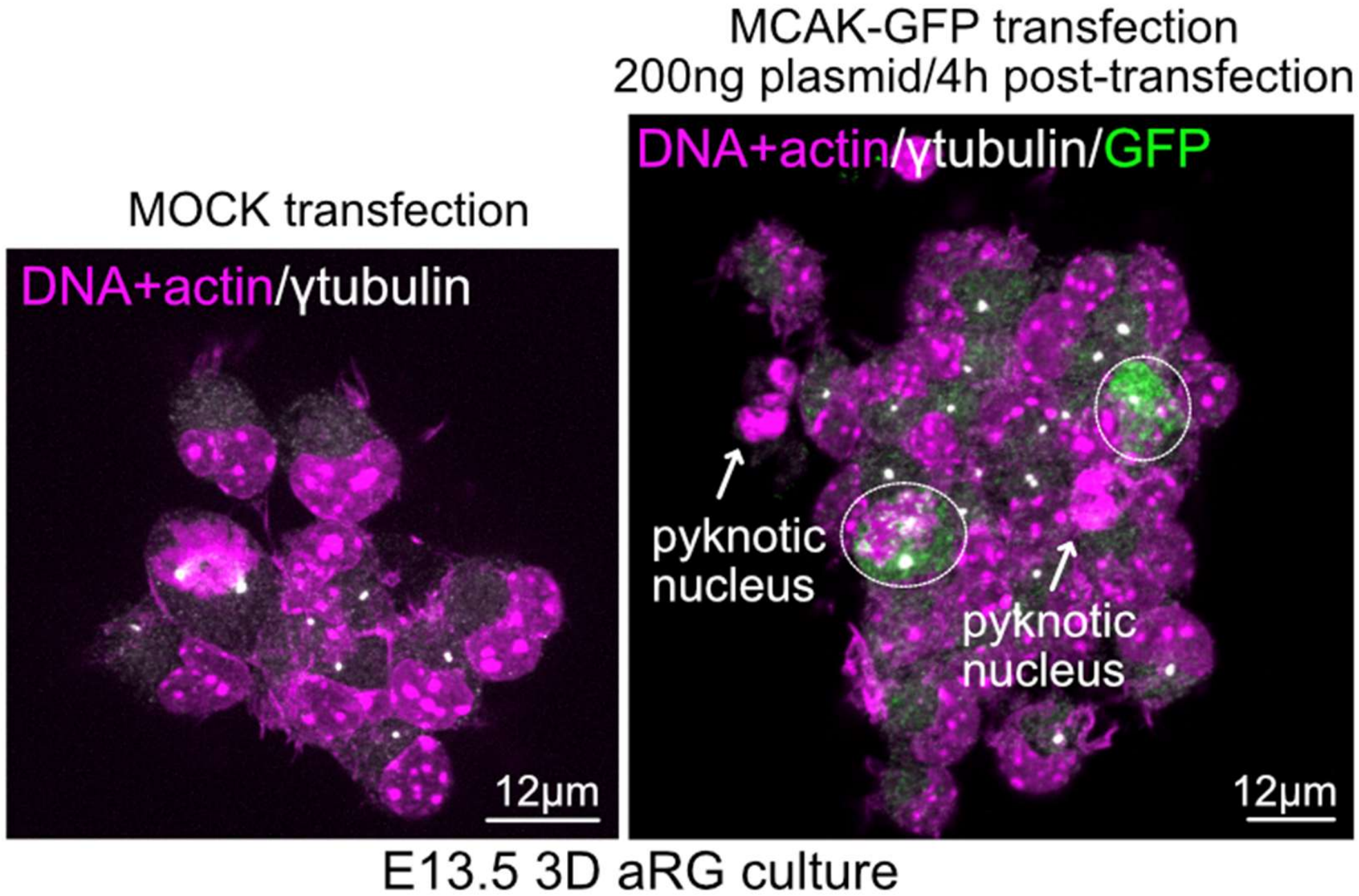

Figure 2: 3D aRG cells labelled with γ-tubulin antibody for centrosomes (grey), GFP antibody for MCAK-GFP (green). Actin and DNA are in magenta. aRG cells were fixed for analysis 4 hours post-transfection with 200ng MCAK-GFP plasmid.

The general idea of tissue mechanics affecting mitotic fidelity is intriguing. The authors should elaborate more on this point in the discussion. In its present form, the authors mentioned/speculate a possible involvement of tissue mechanics in neurodevelopmental disease. This is a very general statement, and this not really clear what the authors exactly means with that. In addition, from a logical point of view: why the most relevant aspect should be disease, rather than physiology? I urge the authors to develop a deeper and more thought-provoking discussion on the possible implications of biomechanics and mitotic fidelity in physiology.
As suggested by the reviewer, we have provided more detailed explanations regarding this aspect of the discussion in the revised version of the manuscript. Our view is that the high cell density, which results from high proliferation rate in the ventricular zone in the first half of neurogenesis represents a vulnerability during mitosis for chromosome segregation. We now provide new data indicating that aRGs are more responsive to the same load of mechanical constraint at E13.5 than at E16.5 regarding their capacity to segregate chromosomes accurately (new panels Figure 4H-I and EV4F-G). Our view is that this early mechano-sensitivity is primarily required to adjust the rate of microtubule polymerization from the spindle poles and maintain the population of astral microtubules for proper positioning of aRG cells in the neuroepithelium. Failure to do so would result in dysplasia of the tissue and disorganized cortical layering at birth. Nonetheless, a drawback of this mechano-sensitivity and high rate of microtubule polymerization in the aRG population is an increased probability to generate mitotic errors. As safeguard mechanisms exist to detect and correct these errors and to eliminate unfit cells from the cycling population

(Shi *et al*, 2019; Phan *et al*, 2021), the consequences on brain size is undetectable. It is only in pathological conditions that this vulnerability is unraveled with consequences on brain size and functionality. We have added this point in the discussion section.

In that respect, and following the line of thoughts suggested by their data, the authors should consider/discuss the possibility that basal mitosis (that are typically happening in a less crowded niche compared to the ventricular zone), could show a higher mitotic fidelity compared to apical mitosis. This is a very interesting point to consider, as one of the main arguments for the evolutionary switch to basal mitosis has always been the maximization of the total number of mitosis per ventricular surface lengh. The data shown by the authors make me wonder if the switch to a higher proportion of basal mitosis could be linked to the need of a higher mitotic fidelity as well. I am aware that this might not be the main interest of the Basto lab, but if the authors were to have quantifications on the occurrence of lagging chromosomes in apical vs basal mitosis, that could be mentioned and discussed. I believe this will increase tremendously the interest of the manuscript for the brain evo-devo community.
The reviewer is right. Since progenitors positioned away from the apical ventricular zone, undergo mitosis in a less crowded environment, they should be less prone to mitotic errors when facing the same mitotic challenge than apical mitosis. This was a very attractive hypothesis. We have tested it and included the results obtained in the revised version of the manuscript. Indeed, BPs, which have delaminated from the ventricular zone and lost their apico-basal polarity displayed better mitotic accuracy than APs/aRGs, when submitted to the same mitotic challenge (new panels Figure 2C-D). We discard the possibility that loss of polarity is a main factor that contributes, as aRGs keep mechanosensitivity after dissociation from the cerebral cortices and loss of polarity in our hands. This capacity to segregate chromosomes more accurately is accompanied by a decrease in the population of astral MTs described previously (Mora-Bermúdez *et al*, 2014). We therefore propose that the reduced requirement for astral microtubules to interact with the plasma membrane contributes to the assembly of a mitotic spindle that is more functional at segregating chromosomes correctly.

Minor concerns:

The authors use the terms early neurogenesis and late neurogenesis when referring to E13 and E16, respectively. However, in the field of developmental neuroscience early neurogenesis is typically referred to E10-E11. I advise the authors to change the phrasing, to make the classification more sound and less misleading to neurogenesis experts.

Yes, the reviewer is right. It was more like taking into consideration, earlier stages than E16.5. We made these changes according to the reviewer' recommendation.

Paragraph 174-186 "Since MT polymerization activity is asymmetric between the two spindle poles, we plotted the data of the pole displaying the highest activity." Is the MT polymerization activity regulated proportionally at the two spindle poles?

In other words, it would be interesting (using the data the authors gathered already) to understand if constraints are making the spindle more asymmetric. This is a basic tissue cell biology question the authors have the tools to address. I invite them to do so.

This is a very good point. We have now included these data in the revised version of the manuscript (Figure EV3B). In fact, we have observed that the differences in MT nucleation capacity between the two poles are preserved independently of whether aRG cells undergo mitosis with neighbors or not. This indicates that asymmetry in microtubule polymerization capacity between the two spindle poles is not influenced by biomechanical constraints. These observations are in agreement with the asymmetry observed *in vivo,* that do not change between E13.5 and E16.5, as reported in (Vargas-Hurtado et al., 2019).

Bibliography

Arai Y, Pulvers JN, Haffner C, Schilling B, Nüsslein I, Calegari F & Huttner WB (2011) Neural stem and progenitor cells shorten S-phase on commitment to neuron production. *Nat Commun* 2

Arai Y & Taverna E (2017) Neural progenitor cell polarity and cortical development. *Front Cell Neurosci* 11: 1–11

Barroso-Vilares M, Macedo JC, Reis M, Warren JD, Compton D & Logarinho E (2020) Small-molecule inhibition of aging-associated chromosomal instability delays cellular senescence. *EMBO Rep* 21

Calegari F, Haubensak W, Haffher C & Huttner WB (2005) Selective lengthening of the cell cycle in the neurogenic subpopulation of neural progenitor cells during mouse brain development. *Journal of Neuroscience* 25: 6533–6538

Caridi CP, D'agostino C, Ryu T, Zapotoczny G, Delabaere L, Li X, Khodaverdian VY, Amaral N, Lin E, Rau AR, *et al* (2018) Nuclear F-actin and myosins drive relocalization of heterochromatic breaks. *Nature* 559: 54–60

Gogendeau D, Siudeja K, Gambarotto D, Pennetier C, Bardin AJ & Basto R (2015) Aneuploidy causes premature differentiation of neural and intestinal stem cells. *Nat Commun* 6: 1–15

Iwashita M, Kataoka N, Toida K & Kosodo Y (2014) Systematic profiling of spatiotemporal tissue and cellular stiffness in the developing brain. *Development* 141: 3793–3798

Kadowaki M, Nakamura S, Machon O, Krauss S, Radice GL & Takeichi M (2007) N-cadherin mediates cortical organization in the mouse brain. *Dev Biol* 304: 22–33

Krndija D, Marjou F El, Guirao B, Richon S, Leroy O, Bellaiche Y, Hannezo E & Vignjevic DM (2019) Active cell migration is critical for steady-state epithelial turnover in the gut. *Science (1979)* 365: 705–710

Lancaster OM & Baum B (2014) Shaping up to divide: Coordinating actin and microtubule cytoskeletal remodelling during mitosis. *Semin Cell Dev Biol* 34: 109–115

Lizarraga SB, Margossian SP, Harris MH, Campagna DR, Han AP, Blevins S, Mudbhary R, Barker JE, Walsh CA & Fleming MD (2010) Cdk5rap2 regulates centrosome function and chromosome segregation in neuronal progenitors. *Development* 137: 1907–1917

López-Gay JM, Nunley H, Spencer M, di Pietro F, Guirao B, Bosveld F, Markova O, Gaugue I, Pelletier S, Lubensky DK, *et al* (2020) Apical stress fibers enable a scaling between cell mechanical response and area in epithelial tissue. *Science (1979)* 370

Marjanović M, Sánchez-huertas C, Terré B, Gómez R, Scheel F, Pacheco S, Knobel PA, Martínez-marchal A, Palenzuela L, Wolfrum U, *et al* (2016) cep63 Reveals a Role for the Centrosome in Meiotic Recombination. *Nat Commun*

Marthiens V, Rujano M a, Pennetier C, Tessier S, Paul-Gilloteaux P & Basto R (2013) Centrosome amplification causes microcephaly. *Nat Cell Biol* 15: 731–40

Mora-Bermúdez F, Matsuzaki F & Huttner WB (2014) Specific polar subpopulations of astral microtubules control spindle orientation and symmetric neural stem cell division. *Elife* 3

Nikalayevich E, Letort G, de Labbey G, Todisco E, Shihabi A, Turlier H, Voituriez R, Yahiatene M, Pollet-Villard X, Innocenti M, *et al* (2024) Aberrant cortex contractions impact mammalian oocyte quality. *Dev Cell* 59: 841-852.e7

Palumbieri MD, Merigliano C, González-Acosta D, Kuster D, Krietsch J, Stoy H, von Känel T, Ulferts S, Welter B, Frey J, *et al* (2023) Nuclear actin polymerization rapidly mediates replication fork remodeling upon stress by limiting PrimPol activity. *Nat Commun* 14: 1–15

Perrin L, Gannavarapu VR, Pérez-González C, Rivera C, Descroix S, Lennon-Duménil AM, Bellaïche Y, Krndija D & Matic Vignjevic D (2025) The Arp2/3 complex maintains gut epithelial integrity under mechanical challenge. *Current Biology* 35: 4827-4836.e5

Phan TP, Maryniak AL, Boatwright CA, Lee J, Atkins A, Tijhuis A, Spierings DC, Bazzi H, Foijer F, Jordan PW, *et al* (2021) Centrosome defects cause microcephaly by activating the 53BP1-USP28-TP53 mitotic surveillance pathway. *EMBO J* 40

Rujano MA, Sanchez-Pulido L, Pennetier C, le Dez G & Basto R (2013) The microcephaly protein Asp regulates neuroepithelium morphogenesis by controlling the spatial distribution of myosin II. *Nat Cell Biol* 15: 1294–306

Shi L, Qalieh A, Lam MM, Keil JM & Kwan KY (2019) Robust elimination of genome-damaged cells safeguards against brain somatic aneuploidy following Knl1 deletion. *Nat Commun* 10: 1–14

Taubenberger A V., Baum B & Matthews HK (2020) The Mechanics of Mitotic Cell Rounding. *Front Cell Dev Biol* 8: 1–16

Vargas-Hurtado D, Brault JB, Piolot T, Leconte L, Da Silva N, Pennetier C, Baffet A, Marthiens V & Basto R (2019a) Differences in Mitotic Spindle Architecture in Mammalian Neural Stem Cells Influence Mitotic Accuracy during Brain Development. *Current Biology* 29: 2993-3005.e9

Viais R, Fariña-Mosquera M, Villamor-Payà M, Watanabe S, Palenzuela L, Lacasa C & Lüders J (2021) Augmin deficiency in neural stem cells causes p53-dependent apoptosis and aborts brain development. *Elife* 10: 1–25

Wang PS, Chou FS, Ramachandran S, Xia S, Chen HY, Guo F, Suraneni P, Maher BJ & Li R (2016) Crucial roles of the Arp2/3 complex during mammalian corticogenesis. *Development (Cambridge)* 143: 2741–2752

Wordeman L, Wagenbach M & Vicente JJ (2025) MCAK / Kif2C centromeric activity level tunes K-fiber stability.

Dr. Renata Basto
Institut Curie
UMR144
12 rue Lhomond
Paris 75005
France

9th Jun 2026

Re: EMBOJ-2025-122764R
Brain biomechanics governs mitotic fidelity of embryonic neural stem cells

Dear Renata,

Thank you for submitting your revised manuscript on the effect of biomechanical constraints on neuronal mitotic fidelity for our consideration. It has now been seen once more by the three original referees (comments copied below), who were fully satisfied with the revisions and only note a few remaining presentational issues that I would ask you to incorporate during a final round of minor revision. In addition, there are a few editorial issues that should be taken care of at this stage:

- Please enter valid email addresses for all coauthors in the submission system, so that they could be informed about the submission and final decision. At resubmission, acknowledgement emails failed to be delivered to Anne-Sophie Mace.

- Please adjust the order of the manuscript sections, and also make sure to use the correct section headers:
Single title page with complete (full-name!) author information, Abstract, Introduction, Results, Discussion, Methods, Data Availability, Acknowledgements, Disclosure and Competing Interests Statement, References, Main Figure Legends, Tables, Expanded Figure Legends.

- As we are switching from a free-text author contribution statement towards a more formal statement based on Contributor Role Taxonomy (CRediT) terms, please remove the present Author Contribution section and instead specify each author's contribution(s) directly in the Author Information page of our submission system during upload of the final manuscript. See https://casrai.org/credit/ for more information.

- Please carefully go through the reference list and make sure that all references have their complete citation information - including year, journal name & volume, and page/elocator numbers - such information is currently missing for several of them. Please include DOI information/URLs only in case of advance publications that do not yet have volume/page numbers, and reserve the PREPRINT label only for actual preprints on respective server platforms.

- I wonder if the title could be revised to become slightly more explicit and accessible also to a broader readership of non-specialists - with especially the term 'brain biomechanics' remaining a bit vague/general right now? If helpful, we could go somewhat over the 100-character limit stipulated in our guidelines.

- Please provide suggestions for a short 'blurb' text prefacing and summing up the conceptual aspect of the study in two sentences (max. 250 characters), followed by 3-5 one-sentence 'bullet points' with brief factual statements of key results of the paper; they will form the basis of an editor-written 'Synopsis' accompanying the online version of the article. Please also upload a synopsis image, which can be used as a "visual title" for the synopsis section of your paper. The image (maybe based on a compacted and landscape-format-re-arranged version of Figure 7?) should be ideally in JPG format, and please make sure that it remains in the modest dimensions of (exactly) 550 pixels wide and between 300 pixels high.

- Finally, during routine pre-acceptance checks, our data editors have raised the following queries regarding figures, data, and legends; I would appreciate if you briefly answered to them in the cover letter of your final submission, and made the requested text modifications with changes/additions highlighted via the "Track changes" option, to facilitate our final checking":
1. Please provide the exact p values in the legends of figures 1C, F, I, J; 2B, F, G; 3D, E, G, H, J, K; 4B, E; 5C, F, K, L, N; 6F, H; EV1 B, EV2 G, EV3 B, C, H, L; EV4 G, EV5 C, D, J, K; EV6 A, EV7 D, H.
2. Please define the scale bar for figure 1B
3. Please define the dotted borders in the legend of figure 4A, H; 5D, 6G, EV1 A.

I am returning the manuscript to you for a final round of minor revision, solely to allow you to make these modifications and upload the revised files. Once we will have received them, we should be ready to swiftly proceed with formal acceptance and production of the manuscript.

With kind regards,

Hartmut

Hartmut Vodermaier, PhD
Senior Editor, The EMBO Journal
h.vodermaier@embojournal.org

*** PLEASE NOTE: All revised manuscript are subject to initial checks for completeness and adherence to our formatting guidelines. Revisions may be returned to the authors and delayed in their editorial re-evaluation if they fail to comply to the following requirements. As a first step please read our guidelines for revised submissions:
https://link.springer.com/journal/44318/submission-guidelines#cms-Revised-submissions

1) Every manuscript requires a Data Availability section (even if only stating that no deposited datasets are included). Primary datasets or computer code produced in the current study have to be deposited in appropriate public repositories prior to resubmission, and reviewer access details provided in case that public access is not yet allowed.

2) Each figure legend must specify
- size of the scale bars that are mandatory for all micrograph panels
- the statistical test used to generate error bars and P-values
- the type error bars (e.g., S.E.M., S.D.)
- the number (n) and nature (biological or technical replicate) of independent experiments underlying each data point
- Figures may not include error bars for experiments with n<3; scatter plots showing individual data points should be used instead.

3) Revised manuscript text (including main tables, and figure legends for main and EV figures) has to be submitted as editable text file (e.g., .docx format). We encourage highlighting of changes (e.g., via text color) for the referees' reference.

4) Each main and each Expanded View (EV) figure should be uploaded as individual production-quality files (preferably in .eps, .tif, .jpg formats). For suggestions on figure preparation/layout, please refer to our Figure Preparation Guidelines:
https://media.springernature.com/original/springer-cms/rest/v1/content/27825798/data/v1

5) Point-by-point response letters should include the original referee comments in full together with your detailed responses to them (and to specific editor requests if applicable), and also be uploaded as editable (e.g., .docx) text files.

6) Please complete our Author Checklist, and make sure that information entered into the checklist is also reflected in the manuscript; the checklist will be available to readers as part of the Review Process File.

7) All authors listed as (co-)corresponding need to deposit, in their respective author profiles in our submission system, a unique ORCiD identifier linked to their name. Please see our Guide to Authors for detailed instructions.

8) Please note that supplementary information at EMBO Press has been superseded by the 'Expanded View' for inclusion of additional figures, tables, movies or datasets; with up to five EV Figures being typeset and directly accessible in the HTML version of the article.

9) To facilitate reproducibilty and cross-laboratory adoption of methodologies, please structure the Materials & Methods section as outlined in our guide to authors, including a completed Reagents and Tools Table.

10) Digital image enhancement is acceptable practice, as long as it accurately represents the original data and conforms to community standards. If a figure has been subjected to significant electronic manipulation, this must be clearly noted in the figure legend and/or the 'Materials and Methods' section. The editors reserve the right to request original versions of figures and the original images that were used to assemble the figure. Finally, we generally encourage uploading of numerical as well as gel/blot image source data.

At EMBO Press we ask authors to provide source data for the main manuscript figures. You will receive a separate email with instructions for providing source data with your revised manuscript, including how to upload and organize the files.

In the interest of ensuring the conceptual advance provided by the work, we recommend submitting a revision within 3 months (7th Sep 2026). Please discuss the revision progress ahead of this time with the editor if you require more time to complete the revisions. Use the link below to submit your revision:

Link Not Available

Referee #1:

The authors have largely incorporated my initial comments, and I am now supportive of the publication of this manuscript. One minor comment: please adjust Figure 3A to a colorblind-friendly version.

Referee #2:

With the new version, the authors have substantially revised and improved their manuscript, adding important new experiments and rewriting key sections to clarify and better substantiate their findings.
Especially the addition of new aspects in the introduction and discussion about a potential trade-off between mitotic fidelity and cortical morphogenesis, which may only become apparent under pathological conditions broadens the conceptual framework of the study.
This reviewer further agrees with the authors' arguments that the acute chemical interventions in the particular context of their study are the most appropriate experimental framework and despite their inherent limitations remain valid due to the fact that all questions are tackled by more than one intervention.

I only have some very small cosmetical additional suggestions:

- Although reflecting the cellular scale, work from the Buzz Baum lab demonstrating a correlation between cell confinement and spindle positioning should be cited also in the introduction.
- aRG should also be defined in the introduction not only in the abstract.
- Line 134-136 the author cite previous work as if it was part of the current study. This should be rephrased and adequately cited.
- Line 238 do the authors mean flattened morphology instead of flattened aspect?
- For future reference, it would massively help the reviewers if Figures would be annotated on the Figure page in the combined document (e.g. Mathiens et al., Figure EV X)

Referee #3:

With the revised version of the manuscript, the authors have addressed the majority of my initial concerns in a satisfactory manner. The revisions have improved the clarity and overall strength of the study. One point, however, remains only partially addressed - namely, the use of genetic perturbation to corroborate the findings obtained through pharmacological approaches. While this additional line of evidence would have further strengthened the conclusions, the authors provide a reasonable and well-explained justification for not including such experiments. Taken together, I believe the current version of the manuscript is significantly improved.
The manuscript can be accepted in its current version.

Response to the reviewers' comments:

For all the changes made, we have kept the "Track changes" option

Referee 2:

1. Although reflecting the cellular scale, work from the Buzz Baum lab demonstrating a correlation between cell confinement and spindle positioning should be cited also in the introduction.

As suggested by the referee, we have now included these references in the introduction part, lines 138-142.

2. aRG should also be defined in the introduction not only in the abstract.
We have defined the abbreviation in the introduction section line 114.

3. Line 134-136 the author cite previous work as if it was part of the current study. This should be rephrased and adequately cited.
We have rephrased and adequately cited our previous work, lines 165-166 and 186-187.

4. Line 238 do the authors mean flattened morphology instead of flattened aspect?
We meant « flattened morphology », we made the appropriate change line 291.

EMBO Press Author Checklist

Corresponding Author Name: Renata Basto and Veronique Marthiens	
Journal Submitted to: The EMBO journal	
Manuscript Number: EMBOJ-2025-122764	

USEFUL LINKS FOR COMPLETING THIS FORM
The EMBO Journal - Author Guidelines
EMBO Reports - Author Guidelines
Molecular Systems Biology - Author Guidelines
EMBO Molecular Medicine - Author Guidelines

Reporting Checklist for Life Science Articles (updated January
This checklist is adapted from Materials Design Analysis Reporting (MDAR) Checklist for Authors. MDAR establishes a minimum set of requirements in transparent reporting in the life sciences (see Statement of Task: 10.31222/osf.io/9sm4x). Please follow the journal's guidelines in preparing your
Please note that a copy of this checklist will be published alongside your article.

Abridged guidelines for figures
1. Data
The data shown in figures should satisfy the following conditions:
- ➞ the data were obtained and processed according to the field's best practice and are presented to reflect the results of the experiments in an accurate and unbiased manner.
- ➞ ideally, figure panels should include only measurements that are directly comparable to each other and obtained with the same assay.
- ➞ plots include clearly labeled error bars for independent experiments and sample sizes. Unless justified, error bars should not be shown for technical
- ➞ if n<5, the individual data points from each experiment should be plotted. Any statistical test employed should be justified.
- ➞ Source Data should be included to report the data underlying figures according to the guidelines set out in the authorship guidelines on Data

2. Captions
Each figure caption should contain the following information, for each panel where they are relevant:
- ➞ a specification of the experimental system investigated (eg cell line, species name).
- ➞ the assay(s) and method(s) used to carry out the reported observations and measurements.
- ➞ an explicit mention of the biological and chemical entity(ies) that are being measured.
- ➞ an explicit mention of the biological and chemical entity(ies) that are altered/varied/perturbed in a controlled manner.
- ➞ the exact sample size (n) for each experimental group/condition, given as a number, not a range;
- ➞ a description of the sample collection allowing the reader to understand whether the samples represent technical or biological replicates (including how many animals, litters, cultures, etc.).
- ➞ a statement of how many times the experiment shown was independently replicated in the laboratory.
- ➞ definitions of statistical methods and measures:
  - common tests, such as t-test (please specify whether paired vs. unpaired), simple χ2 tests, Wilcoxon and Mann-Whitney tests, can be unambiguously identified by name only, but more complex techniques should be described in the methods section;
  - are tests one-sided or two-sided?
  - are there adjustments for multiple comparisons?
  - exact statistical test results, e.g., P values = x but not P values < x;
  - definition of 'center values' as median or average;
  - definition of error bars as s.d. or s.e.m.

Please complete ALL of the questions below.
Select "Not Applicable" only when the requested information is not relevant for your study.

Materials

Newly Created Materials	Information included in the manuscript?	In which section is the information available? (Reagents and Tools Table, Materials and Methods, Figures, Data Availability Section)
New materials and reagents need to be available; do any restrictions apply?	Not Applicable	

Antibodies	Information included in the manuscript?	In which section is the information available? (Reagents and Tools Table, Materials and Methods, Figures, Data Availability Section)
For **antibodies** provide the following information:<br>- Commercial antibodies: RRID (if possible) or supplier name, catalogue number and or/clone number<br>- Non-commercial: RRID or citation	Yes	Reagents and Tools Table + Material and Methods

DNA and RNA sequences	Information included in the manuscript?	In which section is the information available? (Reagents and Tools Table, Materials and Methods, Figures, Data Availability Section)
**Short novel DNA or RNA including primers, probes**: provide the sequences.	Not Applicable	

Cell materials	Information included in the manuscript?	In which section is the information available? (Reagents and Tools Table, Materials and Methods, Figures, Data Availability Section)
**Cell lines:** Provide species information, strain. Provide accession number in repository **OR** supplier name, catalog number, clone number, and/**OR** RRID.	Not Applicable	
**Primary cultures:** Provide species, strain, sex of origin, genetic modification status.	Yes	Reagents and Tools Table + Material and Methods
Report if the cell lines were recently **authenticated** (e.g., by STR profiling) and tested for mycoplasma contamination.	Not Applicable	

Experimental animals	Information included in the manuscript?	In which section is the information available? (Reagents and Tools Table, Materials and Methods, Figures, Data Availability Section)
**Laboratory animals or Model organisms:** Provide species, strain, sex, age, genetic modification status. Provide accession number in repository **OR** supplier name, catalog number, clone number, **OR** RRID.	Yes	Reagents and Tools Table + Material and Methods
**Animal observed in or captured from the field:** Provide species, sex, and age where possible.	Not Applicable	
Please detail **housing and husbandry conditions**.	Yes	Material and Methods

Plants and microbes	Information included in the manuscript?	In which section is the information available? (Reagents and Tools Table, Materials and Methods, Figures, Data Availability Section)
**Plants:** provide species and strain, ecotype and cultivar where relevant, unique accession number if available, and source (including location for collected wild specimens).	Not Applicable	
**Microbes:** provide species and strain, unique accession number if available, and source.	Not Applicable	

Human research participants	Information included in the manuscript?	In which section is the information available? (Reagents and Tools Table, Materials and Methods, Figures, Data Availability Section)
If collected and within the bounds of privacy constraints report on age, sex and gender or ethnicity for all study participants.	Not Applicable	

Core facilities	Information included in the manuscript?	In which section is the information available? (Reagents and Tools Table, Materials and Methods, Figures, Data Availability Section)
If your work benefited from core facilities, was their service mentioned in the acknowledgments section?	Yes	Acknowledgement section

Design

Study protocol	Information included in the manuscript?	In which section is the information available? (Reagents and Tools Table, Materials and Methods, Figures, Data Availability Section)
If study protocol has been **pre-registered, provide DOI in the manuscript**. For clinical trials, provide the trial registration number **OR** cite DOI.	Not Applicable	
Report the **clinical trial registration number** (at ClinicalTrials.gov or equivalent), where applicable.	Not Applicable	

Laboratory protocol	Information included in the manuscript?	In which section is the information available? (Reagents and Tools Table, Materials and Methods, Figures, Data Availability Section)
Provide DOI OR other citation details if **external detailed step-by-step protocols** are available.	Not Applicable	

Experimental study design and statistics	Information included in the manuscript?	In which section is the information available? (Reagents and Tools Table, Materials and Methods, Figures, Data Availability Section)
Include a statement about **sample size** estimate even if no statistical methods were used.	Yes	Figure
Were any steps taken to minimize the effects of subjective bias when allocating animals/samples to treatment (e.g. **randomization procedure**)? If yes, have they been described?	Not Applicable	
Include a statement about **blinding** even if no blinding was done.	Yes	Material and Methods section
Describe **inclusion/exclusion criteria** if samples or animals were excluded from the analysis. Were the criteria pre-established?<br><br>If sample or data points were omitted from analysis, report if this was due to attrition or intentional exclusion and provide justification.	Not Applicable	
For every figure, are **statistical tests** justified as appropriate? Do the data meet the assumptions of the tests (e.g., normal distribution)? Describe any methods used to assess it. Is there an estimate of variation within each group of data? Is the variance similar between the groups that are being statistically compared?	Yes	Figure captions, source data

Sample definition and in-laboratory replication	Information included in the manuscript?	In which section is the information available? (Reagents and Tools Table, Materials and Methods, Figures, Data Availability Section)
In the figure legends: state number of times the experiment was **replicated** in laboratory.	Yes	Figure captions, source data
In the figure legends: define whether data describe **technical or biological replicates**.	Yes	Figure captions, source data

Ethics

Ethics	Information included in the manuscript?	In which section is the information available? (Reagents and Tools Table, Materials and Methods, Figures, Data Availability Section)
Studies involving **human participants**: State details of **authority granting ethics approval** (IRB or equivalent committee(s), provide reference number for approval.	Not Applicable	
Studies involving **human participants**: Include a statement confirming that **informed consent** was obtained from all subjects and that the experiments conformed to the principles set out in the WMA Declaration of Helsinki and the Department of Health and Human Services Belmont Report.	Not Applicable	
Studies involving **human participants:** For publication of **patient photos**, include a statement confirming that consent to publish was obtained.	Not Applicable	
Studies involving experimental **animals**: State details of **authority granting ethics approval** (IRB or equivalent committee(s), provide reference number for approval. Include a statement of compliance with ethical regulations.	Yes	Material and Methods section
Studies involving **specimen and field samples:** State if relevant **permits** obtained, provide details of authority approving study; if none were required, explain why.	Not Applicable	

Dual Use Research of Concern (DURC)	Information included in the manuscript?	In which section is the information available? (Reagents and Tools Table, Materials and Methods, Figures, Data Availability Section)
Could your study fall under dual use research restrictions? Please check biosecurity documents and list of **select agents and toxins** (CDC): https://www.selectagents.gov/sat/list.htm	Not Applicable	
If you used a select agent, is the security level of the lab appropriate and reported in the manuscript?	Not Applicable	
If a study is subject to dual use research of concern regulations, is the name of the **authority granting approval and reference number** for the regulatory approval provided in the manuscript?	Not Applicable	

Reporting

The MDAR framework recommends adoption of discipline-specific guidelines, established and endorsed through community initiatives. Journals have their own policy about requiring specific guidelines and recommendations to complement MDAR.

Adherence to community standards	Information included in the manuscript?	In which section is the information available? (Reagents and Tools Table, Materials and Methods, Figures, Data Availability Section)
State if relevant guidelines or checklists (e.g., **ICMJE, MIBBI, ARRIVE, PRISMA**) have been followed or provided.	Not Applicable	
For **tumor marker prognostic studies,** we recommend that you follow the **REMARK** reporting guidelines (see link list at top right). See author guidelines, under 'Reporting Guidelines'. Please confirm you have followed these guidelines.	Not Applicable	
For **phase II and III randomized controlled trials**, please refer to the **CONSORT** flow diagram (see link list at top right) and submit the CONSORT checklist (see link list at top right) with your submission. See author guidelines, under 'Reporting Guidelines'. Please confirm you have submitted this list.	Not Applicable	

Data Availability

Data availability	Information included in the manuscript?	In which section is the information available? (Reagents and Tools Table, Materials and Methods, Figures, Data Availability Section)
Have **primary datasets** been deposited according to the journal's guidelines (see 'Data Deposition' section) and the respective accession numbers provided in the Data Availability Section?	Not Applicable	
Were **human clinical and genomic datasets** deposited in a public access-controlled repository in accordance to ethical obligations to the patients and to the applicable consent agreement?	Not Applicable	
Are **computational models** that are central and integral to a study available without restrictions in a machine-readable form? Were the relevant accession numbers or links provided?	Yes	Material and Methods section
If publicly available data were reused, provide the respective **data citations in the reference list**.	Not Applicable	
